# Benchmarking Statistical Methods for Environmental Chemical Mixtures: Prediction, Interaction Detection, and an Applied Analysis of Metals, Essential Elements and Diabetes

**DOI:** 10.3390/stats9050096

**Published:** 2026-09-04

**Authors:** Aderonke Gbemi Adetunji, Emmanuel Obeng-Gyasi

**Affiliations:** 1 Department of Built Environment, North Carolina A&T State University, Greensboro, NC 27411, USA; 2 Environmental Health and Disease Laboratory, North Carolina A&T State University, Greensboro, NC 27411, USA

**Keywords:** environmental mixtures, interaction detection, simulation benchmark, Bayesian kernel machine regression, Lasso, hierarchical interactions, NHANES, diabetes

## Abstract

**Background.:**

Human populations are exposed to complex chemical mixtures, making interaction detection a central challenge in environmental epidemiology. We benchmarked methods for prediction and recovery of interaction structure.

**Methods.:**

Eight approaches—main-effects Lasso (glmnet_main), interaction Lasso (glmnet_int), hierNet, Random Forests, Bayesian Kernel Machine Regression (BKMR), quantile g-computation (qgcomp), weighted quantile sum regression (gWQS) and SuperLearner—were evaluated across eight linear/nonlinear, additive/interaction, continuous/binary data-generating processes (500 replicates each). Every method completed in all 500 replicates of all eight scenarios. Prediction was assessed on held-out test data using observed-outcome and oracle-referenced metrics; interaction detection was assessed against three known pairwise interactions among 45 candidate pairs, using both hard selection and a threshold-free ranking criterion. BKMR was evaluated at 2000 versus 25,000 MCMC iterations with multi-chain convergence diagnostics. Sensitivity analyses varied sample size, exposure correlation, signal strength, and interaction form. BKMR was also applied illustratively to six metals and prevalent diabetes in NHANES.

**Results.:**

In additive settings, observed-outcome prediction was similar across methods, but oracle-referenced continuous-outcome error differed by up to six-fold. With interactions, interaction-aware methods clearly outperformed additive-only approaches on the continuous oracle-referenced metrics: in LMI, the oracle MSE was 1.57 for hierNet and 1.87 for glmnet_int against 3.80 for glmnet_main and 4.94 for qgcomp. glmnet_int and hierNet showed comparable sensitivity; hierNet had a modestly lower mean per-replicate false discovery proportion in paired comparisons, while pooled false discovery favored hierNet in the continuous scenarios and glmnet_int in the binary ones; pooled false discovery rates were 0.79 to 0.82 in every interaction scenario, so roughly four in five selected pairs were false. In the scenarios without true interactions, the pooled false discovery rate was exactly 1. Under threshold-free ranking, BKMR was competitive with the penalized methods (pair-ranking AUC: 0.758 to 0.781 across the four interaction scenarios). BKMR’s apparent instability at 2000 iterations reflected inadequate sampling: 93% of monitored parameters had a Gelman–Rubin statistic above 1.1 and the minimum effective sample size was 7.5, whereas at 25,000 iterations the median statistic was 1.02 and the oracle MSE in LMI fell from 6.38 to 2.08. In NHANES, lead, manganese, and iron had the highest posterior inclusion probabilities, with predominantly nonlinear exposure–response functions.

**Conclusions.:**

Method choice matters most when interactions are present. Interaction-aware methods are preferable when joint effects are relevant, selected interactions require replication given the high false discovery burden, and BKMR comparisons should report sampling budgets and convergence diagnostics rather than treating a short chain as characteristic of the method.

## 1. Introduction

Human populations are exposed to complex mixtures of chemicals, pollutants, and environmental stressors rather than to single agents in isolation. The exposome framework, which encompasses the totality of environmental exposures, helps clarify how multiple simultaneous exposures, air pollution, heavy metals, pesticides, industrial chemicals, diet, and psychosocial stress, jointly shape health outcomes [1]. A persistent challenge in exposome research is detecting and characterizing interactions among the components of environmental mixtures [2,3]. Because real-world exposure involves many chemicals acting at the same time, the combined effect of a mixture need not equal the sum of its individual effects: interactions may be synergistic, with joint exposure producing greater harm than expected, or antagonistic, with one exposure attenuating the effect of another [2,3].

Statistical methods for analyzing environmental mixtures have grown substantially in recent years, each with distinct assumptions, strengths, and limitations [4,5]. Despite this progress, important challenges remain in evaluating the joint health effects of mixtures. Mixture components are often strongly correlated, dose–response relationships are frequently nonlinear and nonadditive, and the number of pollutants and their potential pairwise interactions can be large [4,5]. These effects are often small relative to the influence of demographic covariates and are therefore difficult to detect. Taken together, these features are not easily handled by standard regression models.

A range of statistical and machine-learning methods has been developed in response, each with distinct advantages and limitations [4]. Penalized regression approaches such as the Least Absolute Shrinkage and Selection Operator (LASSO), Group Lasso, and Elastic Net support variable selection but struggle when exposures are highly correlated and interactions are complex [6]. LASSO can identify influential mixture components by shrinking unimportant coefficients to zero, yet it tends to retain only one chemical from a group of correlated exposures and is best suited to linear associations [6]. Elastic Net improves prediction in high-dimensional settings by combining ridge and LASSO penalties and handles correlated predictors more effectively, although it may still yield false positives and often requires post-selection inference for valid confidence intervals [7]. Machine-learning approaches such as Random Forest regression capture nonlinear and higher-order relationships without strict distributional assumptions but are limited in their ability to identify specific interaction terms [8]. Bayesian Kernel Machine Regression (BKMR) offers a flexible nonparametric framework for modeling complex exposure–response surfaces, estimating joint mixture effects, and ranking components through posterior inclusion probabilities, although these probabilities can be sensitive to tuning, formal interaction testing is not readily available, and computational demands rise sharply with the number of exposures [9]. Purpose-built mixture methods such as Weighted Quantile Sum (WQS) regression and quantile g-computation estimate overall mixture effects and rank contributors while mitigating collinearity and overfitting, but they emphasize overall effects rather than recovery of specific pairwise interactions and often assume linear additivity or rely on information-reducing quantile transformation [10]. In contrast, the Hierarchical Interaction Network (hierNet) imposes a strong-heredity constraint, requiring both parent main effects before an interaction can be selected, thereby producing more interpretable models aligned with the heredity principle from experimental design [11].

Despite these advances, accurately detecting interactions in environmental mixtures remains difficult for several reasons [4]. Exposures are typically correlated, making it hard to separate main effects from interaction effects. The true data-generating process may be linear or nonlinear, and a method that performs well under one regime may perform poorly under another. Environmental epidemiology routinely involves both continuous and binary outcomes, so methods must be evaluated across outcome types. Finally, practical considerations such as computational efficiency and control of false discoveries become important when screening large numbers of candidate interactions [4].

Given these complexities, a critical gap remains in understanding how reliably analytic approaches recover explicit pairwise interaction structure within chemical mixtures. Recent comprehensive benchmarking by Hao et al. [12] compared many methods across data-generating scenarios for several inferential goals, including identifying important mixture components, identifying interactions among components, and risk prediction. Our study is complementary and more narrowly focused: rather than assessing interaction identification in aggregate, we evaluate explicit pair-level recovery, quantifying for the methods that enumerate pairwise terms how reliably the specific simulated interactions are identified against a known ground truth (pair-level sensitivity, specificity, FDR, and FPR), alongside held-out prediction across linear, nonlinear, continuous, and binary settings [12]. This distinction is important because the ability to identify specific synergistic or antagonistic exposure pairs is central to understanding biological mechanisms, identifying vulnerable subgroups, and informing targeted interventions. The objectives of this study were therefore to:

- Conduct a systematic, simulation-based benchmark of leading mixture methods to assess their held-out predictive performance and their ability to recover the interaction structure among correlated chemical exposures under diverse linear, nonlinear, continuous, and binary-outcome settings;
- Apply Bayesian Kernel Machine Regression to real-world data from the National Health and Nutrition Examination Survey (NHANES) to illustrate the joint association of a toxic-metal and essential-element mixture (lead, cadmium, mercury, selenium, manganese, and iron) with prevalent diabetes.

## 2. Materials and Methods

### 2.1. Simulation Framework

#### 2.1.1. Exposure Generation and Correlation Structure

We simulated a mixture of P = 10 chemical exposures for each subject, denoted X = (X_1_, X_2_, …, X_10_), of which five had nonzero main effects (q = 5). In the interaction scenarios, a sixth exposure, X6, affects the outcome only through the X5-by-X6 interaction and carries no main effect, so six exposures influence the outcome in those data-generating processes. To reflect realistic exposure correlations, we generated exposures from a multivariate normal distribution with a block correlation structure: exposures were divided into two groups of five, with within-group correlation ρ_within = 0.6 and between-group correlation ρ_between = 0.1. This mimics settings in which chemicals from the same source or pathway (e.g., traffic-related pollutants or household products) are more highly correlated than chemicals from different sources. Each exposure was standardized to mean zero and unit variance. To ensure positive definiteness of the covariance matrix, Σ, we performed an eigenvalue decomposition and added a small constant (1 × 10^−6^) to the diagonal whenever any eigenvalue fell below 1 × 10^−8^.

#### 2.1.2. Data-Generating Processes and Effect Sizes

We designed eight data-generating processes (DGPs) representing combinations of model type (linear vs. nonlinear), interaction structure (main effects only vs. interactions present), and outcome type (continuous vs. binary). For both continuous and binary outcomes, the sample size was *n* = 500 for training and *n* = 500 for testing (total N = 1000 per replicate). Each DGP was replicated 500 times independently to yield stable performance estimates.

#### Continuous-Outcome Models

##### Linear model (LM):

$Y=\beta_{0}+\Sigma \;\beta_{j}\cdot X_{j}+\varepsilon$, where j∈{1,…,5} indexes the five active exposures with nonzero main effects and $\varepsilon ∼N(0,\sigma^{2})$. We set $\beta_{0}=0$ with active coefficients β_1_ = 0.6, β_2_ = 0.8, β_3_ = 0.7, β_4_ = 0.5, and β_5_ = 0.9, and all other coefficients were zero. Within each replicate, error variance was calibrated using the systematic component across the combined training and test sample to achieve a target R^2^ = 0.20, with a moderate signal-to-noise ratio, typical of environmental epidemiology, via $\sigma^{2}=\text{Var}(\mu )\times \left(1-R^{2}\right)∕R^{2}$, where μ is the linear predictor.

##### Linear model with interactions (LMI):

$Y=\beta_{0}+\Sigma_{j}\;\beta_{j}\cdot X_{j}+\Sigma_{\left(k,l\right)}g(X_{k},X_{l})+\varepsilon$, where the sum runs over the three true pairs and g is the interaction function defined below; the interaction term is not a pure product. In addition to the five main effects, three pairwise interactions were added between (X_1_, X_2_), (X_3_, X_4_), and (X_5_, X_6_). These pairs were chosen deliberately to span both interaction structures of practical interest: (X_1_, X_2_) and (X_3_, X_4_) satisfy strong heredity, in that both members also carry a main effect, whereas (X_5_, X_6_) violates it, because X_6_ has no main effect (β_6_ = 0). The objective was to evaluate interaction-detection performance under both heredity-satisfying and heredity-violating structures rather than under strong heredity alone. The interaction function was $g(X_{k},X_{l})=0.6\times (X_{k},X_{l})+0.2\times ({X_{k}}^{2}\cdot I(X_{l}>0))$, combining a multiplicative interaction with a quadratic threshold component that is active when X_l_ > 0 and increases with the magnitude of X_k_, including large negative values of X_k_. Error variance was calibrated to R^2^ ≈ 0.20.

##### Nonlinear model (NM):

$Y=\beta_{0}+\Sigma \;f_{j}(X_{j})+\varepsilon$, with smooth nonlinear functions $f_{1}(x)=0.8x+0.5x^{2}$, $f_{2}(x)=1.0\cdot \sin(x)$, $f_{3}(x)=0.9\cdot \log(1+|x|)\cdot \mathrm{sign}(x)$, $f_{4}(x)=0.7x\cdot I(x>0)$ (threshold), and $f_{5}(x)=0.6x∕(1+0.5|x|)$ (saturating). These represent quadratic, oscillating, logarithmic, threshold, and saturating dose–response shapes. Error variance was calibrated to R^2^ ≈ 0.20.

##### Nonlinear model with interactions (NMI):

$Y=\beta_{0}+\Sigma \;f_{j}(X_{j})+\Sigma \;g(X_{k},X_{l})+\varepsilon$, combining the NM main effects with the three LMI interaction terms, the most complex scenario, in which both main effects and interactions are nonlinear.

###### Interaction-term composition and variance decomposition.

The interaction function, g, is asymmetric in its two arguments, because the threshold component is a function of X squared for the first member of the pair and an indicator for the second. Throughout, the first-listed member of each pair takes the role of k: the pairs are ordered (X1, X2), (X3, X4), and (X5, X6), so k indexes X1, X3, and X5 respectively. Decomposing the variance of the systematic component in the interaction scenarios shows that main effects contribute 64.3% of Var(μ) in LMI and 47.5% in NMI, the multiplicative product terms contribute 15.7% and 17.2%, the quadratic threshold component contributes 1.85% and 2.03%, and the remainder is covariance between components. The quadratic threshold term is therefore an order of magnitude smaller than the multiplicative interaction it accompanies, so the additive methods’ penalty in these scenarios is attributable to their inability to model the interaction rather than to unmodeled main-effect curvature. To confirm this directly we additionally ran a pure-product variant in which $g(X_{k},X_{l})=0.6\times X_{k}\times X_{l}$ with the quadratic threshold component removed (Section 2.4, with results in Section 3.6).

#### Binary-Outcome Models

For binary outcomes, we used logistic models Y ~ Bernoulli(p), with $\mathrm{logit}(p)=\alpha +k\cdot \tilde{\mu }$, where μ is the systematic component (linear or nonlinear, with or without interactions) and the standardized systematic component is $\tilde{\mu }=\left(\mu -E[\mu ]\right)∕\surd \text{Var}(\mu )$. Two parameters were calibrated: the intercept α = logit(0.50) = 0 to target an approximately balanced outcome prevalence and the scaling factor, k, such that $\surd \text{Var}(k\cdot \tilde{\mu })=1.25$, ensuring moderate effect sizes on the logit scale. The four binary DGPs were the logistic model (Logit, following LM), logistic with interactions (LogitI, following LMI), nonlinear logistic (NLogit, following NM), and nonlinear logistic with interactions (NLogitI, following NMI).

For each replicate, exposures were generated for the full sample (*n* = 1000) and split 50:50 into training and test sets. Both continuous and binary outcomes were generated for each dataset. Exposures were standardized within training and test sets using training-set parameters to avoid leakage.

### 2.2. Statistical Methods Evaluated

We evaluated eight approaches representing diverse methodological paradigms, described in Sections 2.2.1-2.2.7. Each was implemented with standard R (version 4.4.0) packages using default or recommended hyperparameters to ensure reproducibility and to reflect typical applied practice. For interaction detection, we constructed an expanded design matrix including all 45 possible pairwise interactions (C(10,2) = 45) in addition to the 10 main effects, yielding 55 candidate predictors for methods capable of explicit interaction modeling. All eight methods were evaluated for both continuous and binary outcomes, and all completed every replicate of every scenario; per-method fit counts are reported in Supplementary Tables S4 and S5.

#### 2.2.1. Lasso Regression (glmnet)

Lasso estimates regression coefficients by minimizing the residual sum of squares, or the binomial deviance for a binary outcome, plus an L1 penalty on the coefficient vector; the penalty shrinks small coefficients exactly to zero, so estimation and variable selection are performed simultaneously, and the penalty parameter, λ, controls the strength of that shrinkage. We fitted L1-penalized models using glmnet (4.1-8) in two variants: main effects only (glmnet_main; Y ~ X_1_ + … + X_10_) and with interactions (glmnet_int; the expanded model with all 45 pairwise terms). The penalty, λ, was chosen by 10-fold cross-validation minimizing MSE (continuous) or deviance (binary); interaction terms with nonzero coefficients at the optimal λ were classified as detected [13].

#### 2.2.2. Random Forests (RFs)

A Random Forest averages the predictions of many regression or classification trees, each grown on a bootstrap resample of the training data with a randomly selected subset of predictors considered at each split. Averaging across decorrelated trees reduces variance and allows the fitted model to capture nonlinearities and interactions implicitly without requiring those terms to be specified explicitly [14]. We used the randomForest package (version 4.7-1.1) with ntree = 500, representing the number of trees grown and averaged; default splitting rules and minimum node sizes; and mtry, the number of predictors sampled as candidates at each split, set to ⌊P/3⌋ = 3. Because Random Forests do not return an explicit set of selected pairwise interaction terms, RF was excluded from the hard-selection comparison; it was included in the ranking-based analysis for continuous outcomes using the Friedman H-statistic (Section 2.3.2) [14].

#### 2.2.3. Hierarchical Interaction Network (hierNet)

hierNet enforces strong heredity, permitting an interaction, X_k_:X_l_, only when both parent main effects are present [15]. We used hierNet (1.9) with hierNet.path. For continuous outcomes, λ was selected by 10-fold cross-validation (hierNet.cv), harmonized with the fold count used for glmnet so that the two enumerating methods were tuned identically. For binary outcomes, hierNet.cv in version 1.9 accepts a logistic solution path, so λ was likewise selected by 10-fold cross-validation and the two enumerating methods were tuned identically for both outcome types. We note that predict.hierNet.logistic returns a list rather than a numeric vector; the predicted probability component is extracted explicitly and verified to lie in [0, 1] before scoring, and a fit whose predictions fall outside that range is recorded as a failure rather than transformed. With strong = TRUE, pairs with $|\theta_{\text{kl}}|>1\times 10^{-8}$ were classified as detected. Because this tolerance is arbitrary, we repeated the continuous detection analysis at tolerances of 0, 1 × 10^−12^, 1 × 10^−10^, 1 × 10^−8^, and 1 × 10^−6^ (Section 2.4, with results in Section 3.6).

#### 2.2.4. Bayesian Kernel Machine Regression (BKMR)

BKMR models the exposure–response surface h(Z) as a flexible sum of Gaussian kernels, accommodating nonlinear main effects and interactions without explicit interaction terms. We used bkmr (0.2.2) with kmbayes: family = ‘gaussian’ or ‘binomial’, varsel = TRUE, which performs componentwise variable selection because no exposure grouping was supplied, and est.h = TRUE. Because kmbayes specifies no default iteration count, the budget is the analyst’s choice and we therefore treat it as an experimental factor rather than as a fixed setting. The primary benchmark uses 2000 iterations with 1000 burn-in as the short-budget condition. A budget arm re-fitted BKMR at both 2000 and 25,000 iterations (12,500 burn-in) on 50 paired replicates per scenario, and a diagnostic arm ran four independent chains from dispersed starting values on five datasets at each budget, from which we computed the Gelman–Rubin statistic and the effective sample size with coda, on unthinned post-burn-in draws, for the kernel bandwidth parameters, r1 to r10; the variance component, lambda1; and, for continuous outcomes, the residual variance. The diagnostic arm covered one continuous scenario (LMI) and one binary scenario (LogitI). Test predictions were generated using SamplePred from post-burn-in posterior samples, thinned by 10 for the 2000-iteration budget and by 25 for the 25,000-iteration budget; for binary outcomes, SamplePred was called with type = ‘response’, which returns response-scale probabilities under the probit model, constrained to [1 × 10^−8^, 1 − 1 × 10^−8^]. Under the default sampler configuration with family = ‘binomial’ and componentwise variable selection, kmbayes terminated with a non-positive-definite augmented kernel matrix in every attempted fit at *n* = 500. We therefore evaluated four sampler configurations on three datasets in each binary scenario (Supplementary Table S13b) and found that setting est.h = TRUE, which samples the exposure–response surface explicitly rather than marginalizing it out, completed in every fit. That configuration is used for all BKMR results reported here, for both outcome families, so that the sampler is identical across outcome types; the configuration actually used is recorded for every fit in the deposited output. Runtime is reported as seconds per 1000 iterations so that it is comparable across budgets [16,17].

#### 2.2.5. Quantile g-Computation (qgcomp)

qgcomp estimates the joint effect of a mixture from quantile-scaled exposures and additive weights [18]. Exposures are quantized and entered jointly in a generalized linear model; the overall mixture effect is obtained as the sum of the exposure coefficients and represents the expected change in the outcome associated with a simultaneous one-quantile increase in all mixture components. Unlike Weighted Quantile Sum regression, from which it borrows the quantization step, quantile g-computation imposes no directional constraint on the component weights. We used qgcomp (2.10.0) with qgcomp.noboot, q = 4 quantile categories, and the relevant family. Because standard qgcomp does not model pairwise interactions, we focused on its prediction performance.

#### 2.2.6. Weighted Quantile Sum Regression (gWQS)

Weighted Quantile Sum regression estimates a single mixture index as a weighted sum of quantized exposures, with the weights constrained to be non-negative and to sum to one. The weights are estimated by bootstrap resampling of the training data and are then carried into a generalized linear model in which the index enters as a single term, so the fitted mixture effect is directional by construction and the weights are interpretable as the relative contribution of each exposure to that direction. Because the constraint forces all components to act in a common direction, the estimator is efficient when that assumption holds and biased when exposures act in opposing directions. We used gWQS (3.0.5) with q = 4 quantile categories, b = 50 bootstrap samples, and validation = 0 so that weights were estimated on the full training set, sequential evaluation, and the relevant family. Test-set predictions were formed by applying the training-set quantile breakpoints and the estimated mean weights to the held-out exposures and evaluating the fitted model. gWQS does not model pairwise interactions, so it is evaluated on prediction only.

#### 2.2.7. SuperLearner

SuperLearner is a cross-validation-based ensemble that forms a convex combination of candidate learners, with weights chosen to minimize cross-validated risk. It is not itself a mixture method; it is included as a general-purpose machine-learning benchmark that adapts to whichever library member fits best. We used SuperLearner (2.0-40) with 5-fold cross-validation and a library of SL.glm, SL.mean, SL.glmnet and SL.ranger, and the relevant family. Because SL.ranger is a Random Forest, the library can represent interaction and nonlinearity implicitly even though no candidate learner contains explicit pairwise interaction terms; SuperLearner should therefore not be described as an additive-only method. The library was fixed in advance and did not vary across scenarios or replicates. SuperLearner returns no pairwise interaction parameter, so it is evaluated on prediction only.

### 2.3. Performance Metrics

Prediction and classification metrics were computed on held-out test sets (*n* = 500) not used in training. Interaction-detection metrics were evaluated against the known simulated interaction structure, and computational cost was recorded per fit. Because outcome variance differs across DGPs, prediction-error magnitudes are compared within scenarios rather than across scenarios.

#### 2.3.1. Prediction Performance

For continuous outcomes, we report the mean squared error $\text{MSE}=(1∕n\_\text{test})\cdot \Sigma {\left(Y_{i}-{\hat{Y}}_{i}\right)}^{2}$; lower is better) and the Pearson correlation between observed and predicted values (scale-invariant; higher is better). For binary outcomes, we report the area under the ROC curve (AUC), computed with pROC (1.18.5; [19]), ranging from 0.5 (chance) to 1.0 (perfect discrimination). Because the outcome is generated with a target R^2^ of 0.20 for the continuous data-generating processes, roughly 80% of continuous-outcome variance is irreducible noise by construction, and error measured against the observed outcome is dominated by that noise floor. We therefore also report oracle-referenced metrics computed against the known systematic component: for continuous outcomes, MSE and Pearson correlation against the true conditional mean, μ, and for binary outcomes the Bayes-optimal AUC obtained by ranking test observations on the true linear predictor. The oracle AUC is computable because α and k are fixed by design, and it establishes the ceiling against which the observed AUC of each method should be read.

#### 2.3.2. Interaction-Detection Performance

For scenarios with interactions (LMI, NMI, LogitI, and NLogitI), the ground truth comprised the three nonzero pairwise interactions (X_1_, X_2_), (X_3_, X_4_), and (X_5_, X_6_). Each detection-capable method (glmnet_int and hierNet) classified each of the 45 candidate pairs as detected or not. We computed the sensitivity (TP/(TP + FN)), specificity (TN/(TN + FP)), false discovery rate (FDR = FP/(TP + FP)), and false-positive rate (FPR = FP/(TN + FP) = 1 – specificity). Sensitivity is defined only for scenarios containing true interactions; with three true and 42 null interactions, it takes values 0, 1/3, 2/3, or 1. We report FDR descriptively, as the realized false discovery burden among selected pairs, rather than as evidence that any nominal level (e.g., 0.10) was controlled; because true interactions are rare relative to candidate pairs, FDR is expected to be high and selected interactions warrant replication [20].

##### Aggregation of the false discovery rate.

The per-replicate false discovery proportion is FP/(TP + FP). When a replicate selects no pairs, this quantity is 0/0; it is stored as missing and is not coded as zero. We report two summaries that differ in their treatment of this case. The mean per-replicate false discovery proportion conditions on replicates making at least one selection, and the proportion of replicates selecting nothing is reported alongside it. The pooled false discovery rate is the sum of false positives divided by the sum of all selections across all successful replicates and is the more interpretable summary because it is not distorted by over-dispersion in the number of selections per replicate. We also report the full distribution of the number of pairs selected per replicate. In the null scenarios, there are no true positives by construction, so any replicate selecting at least one pair has a false discovery proportion of exactly 1 and the pooled false discovery rate is exactly 1; the informative null-scenario quantities are the specificity, the false-positive rate, and the number of pairs selected, not the false discovery rate. Because the number of true and null pairs is fixed across replicates, the pooled false discovery rate is algebraically determined by the mean sensitivity and the mean false-positive rate; we report this identity check explicitly, and it holds to within 1 × 10^−16^ in every scenario. Fits that failed are excluded from all detection metrics and reported as a failure rate; they are never recorded as replicates that selected nothing.

##### Ranking-based detection.

Restricting detection to methods that return an explicit list of selected pairs excludes BKMR, which characterizes interaction through its fitted response surface rather than by selecting terms, and Random Forests, which return variable importance but no pairwise interaction measure. To place all four on the same footing, we additionally score detection as a ranking problem. For each replicate, every method assigns a score to all 45 candidate pairs: for glmnet_int the absolute penalized interaction coefficient, for hierNet the symmetrized absolute interaction parameter (∣θ_kl_∣ + ∣θ_lk_∣)/2, for Random Forests the Friedman H-statistic for the pair, and for BKMR a bivariate interaction contrast obtained from the fitted response surface. For a pair (Z_j_, Z_k_), that contrast is the range, across a grid of Z_j_ values, of the difference between h evaluated with Z_k_ fixed at its 75th percentile and h evaluated with Z_k_ fixed at its 25th percentile, with all remaining exposures held at their medians. The range rather than the level is used because under a purely additive surface that difference is a non-zero constant in Z_j_, so its magnitude carries no interaction information, whereas its variation does; the statistic is zero when the two exposures act additively and larger for greater departure from additivity. We then compute, against the three known true pairs, the area under the ROC curve for the ranking, the average precision, the recall among the top three ranked pairs, and the mean rank of the true pairs. Recall is measured against all three true pairs, and for each method–scenario combination with a usable pairwise score a complete 45-pair score vector is required in every replicate, so a method cannot improve its apparent performance by failing to score a difficult pair. The Friedman H-statistic could not be obtained for a classification forest with the implementation used, so Random Forests are scored for the continuous scenarios only and the corresponding binary cells are reported as unusable rather than omitted. Because these metrics use the ordering rather than a hard selection threshold, they require no additional pair-selection cutoff. The ranking analysis used 50 replicates per scenario, with BKMR fitted at 25,000 iterations.

#### 2.3.3. Computational Efficiency

Wall-clock runtime (seconds) was recorded per replicate with proc.time(), including preprocessing, fitting, tuning, prediction, and post-processing. All computations used identical hardware (Intel Xeon processor, Intel Corporation, Santa Clara, CA, USA; 32 GB RAM) under R 4.4.0 on Ubuntu Linux. Runtime is summarized as the mean across 500 replicates per method–scenario combination. For BKMR, wall-clock runtime is a linear function of the analyst-chosen iteration count rather than a property of the algorithm, so we additionally report seconds per 1000 iterations together with the iteration count required for adequate mixing, and any ratio against a non-iterative method is stated with its budget attached.

#### 2.3.4. Monte Carlo Uncertainty and Between-Method Comparison

All performance summaries are reported as the mean across replicates with the standard deviation and the Monte Carlo standard error, defined as the replicate standard deviation divided by the square root of the number of successful replicates. Because every method is applied to the same simulated datasets within a replicate, between-method comparisons are paired. For each metric, we report the mean paired difference against glmnet_int as the reference method, with its Monte Carlo standard error, a 95% interval, and a two-sided *p*-value from a paired test across replicates. The same paired structure is used for the BKMR budget comparison, where the two budgets are applied to identical datasets. Full tables of means, standard deviations, and Monte Carlo standard errors for every method, scenario, and metric are provided as Supplementary Tables S4-S10. Reporting follows the ADEMP structure and the recommendations of Morris, White, and Crowther for the design, analysis, and reporting of simulation studies [21].

### 2.4. Sensitivity Analyses

The primary benchmark fixes the sample size, the exposure correlation structure, the signal strength, and the form of the interaction term. To establish how far the reported orderings depend on that single point in design space, we repeated the benchmark under the following variations, with method-specific replicate counts: six methods were evaluated over 50 replicates per setting and BKMR and gWQS values over 15, so that no method was dropped from the generalizability assessment. The pure-product analysis used 50 replicates per scenario for all eight methods, and the tolerance analysis, which concerns hierNet alone, used 50 replicates per scenario.

#### Sample size.

Training sample and test sample size was set to *n* = 250, 500, and 1000, with the test-set size held at 500, in the two continuous and two binary interaction scenarios.

#### Exposure correlation.

The block correlation structure was varied from the primary setting (within-block 0.6, between-block 0.1) to a low-correlation setting (0.3 and 0.05) and a high-correlation setting (0.8 and 0.3).

#### Signal strength.

The target R2 was raised from 0.20 to 0.30 and 0.40 in the continuous interaction scenarios, spanning the range more typical of applied analyses than the deliberately modest primary setting.

#### Binary signal strength.

For the binary scenarios, the scaling factor, k, is not itself interpretable as an effect size, so we report Tjur’s coefficient of discrimination and McFadden’s pseudo-R2 for the generating model at the primary setting and repeat the binary benchmark across three levels of the latent-scale signal standard deviation.

#### Pure-product interaction.

The interaction function was replaced by the pure multiplicative form g(Xk, Xl) = 0.6 × Xk × Xl, removing the quadratic threshold component, so that the penalty incurred by additive methods can be attributed unambiguously to the interaction rather than to main-effect curvature.

#### Detection tolerance.

The hierNet selection tolerance was varied across 0, 1 × 10^−12^, 1 × 10^−10^, 1 × 10^−8^, and 1 × 10^−6^ to establish whether the reported detection results depend on the arbitrary threshold used to declare an interaction parameter nonzero.

### 2.5. Applied Analysis: Metal Mixture and Diabetes

#### 2.5.1. Study Population and Design

The present analysis utilized data from the 2017–2018 cycle of the National Health and Nutrition Examination Survey (NHANES). NHANES is a nationwide, population-based survey designed to assess the health, nutrition, and environmental exposures of the non-institutionalized U.S. population. The program, administered by the National Center for Health Statistics (NCHS) at the Centers for Disease Control and Prevention (CDC), collects data in two-year cycles using a complex, multistage probability sampling design to ensure national representation across demographic and geographic groups. During the 2017–2018 survey cycle, a total of 16,211 participants were selected from 30 primary sampling sites across the United States and the District of Columbia. Of those selected, 9254 completed the household interview and 8704 underwent a physical examination. Missing exposure and covariate data were addressed through multiple imputation by chained equations; the final analytic sample comprised 5898 participants. All participants provided written informed consent before enrollment. Trained personnel conducted structured interviews and standardized physical examinations, during which blood specimens were collected and analyzed in certified laboratories according to standardized NHANES protocols. All study procedures were approved by the National Center for Health Statistics (NCHS) Research Ethics Review Board. Demographic characteristics, including age, sex, and race/ethnicity, were collected during household interviews using a Computer-Assisted Personal Interviewing (CAPI) system to enhance data accuracy and minimize interviewer bias. Interviews were conducted in multiple languages according to participant preference. Whole-blood concentrations of lead, cadmium, total mercury, manganese, and selenium were measured using inductively coupled plasma mass spectrometry (ICP-MS) at the CDC’s National Center for Environmental Health, while serum iron (μg/dL) was measured in refrigerated serum samples using standardized NHANES laboratory procedures. Diabetes was defined as a composite binary outcome based on physician-diagnosed diabetes, insulin use, fasting plasma glucose ≥126 mg/dL, or glycated hemoglobin (HbA1c) ≥6.5%, consistent with the diagnostic criteria of the American Diabetes Association.

#### 2.5.2. Exposure Variables and Covariates

The exposure mixture comprised six elements: lead (Pb), mercury (Hg), cadmium (Cd), selenium (Se), manganese (Mn), and iron (Fe). Covariates were age, ethnicity, sex, body mass index, smoking status, alcohol use, household income, education, and physical activity. Missing covariate and exposure data were handled by multiple imputation by chained equations (MICE; m = 20, maxit = 20, seed = 123), using predictive mean matching for continuous variables and logistic regression for binary variables. Because BKMR does not provide a pooling routine for its posterior summaries, the imputations were propagated as follows. A separate BKMR model was fitted to each of the m = 20 completed datasets with identical priors, chain length, and burn-in. Posterior draws from the 20 chains were then combined into a single pooled posterior sample, and all reported quantities were computed from that pooled sample: posterior inclusion probabilities as the pooled proportion of draws in which an exposure entered the kernel, and exposure–response functions, single-exposure contrasts, and overall mixture effects as pooled posterior means with equal-tailed 95% intervals taken from the 2.5th and 97.5th percentiles of the pooled draws. Equal numbers of posterior draws were combined across imputations, so that the reported summaries reflect both the within-imputation posterior uncertainty and the between-imputation variability.

#### 2.5.3. Bayesian Kernel Machine Regression

BKMR was selected for the applied analysis because it provides descriptive outputs that the detection-focused methods do not, univariate and bivariate exposure–response surfaces, the overall joint mixture effect, and posterior inclusion probabilities with full Bayesian uncertainty quantification, which are well suited to characterizing how a metal mixture relates to prevalent diabetes. Prevalent diabetes was chosen as the illustrative outcome not for any statistical property but because it is a familiar, well-characterized cardiometabolic endpoint with an established literature on metal exposure; this makes the resulting exposure–response surfaces and mixture summaries easy to interpret and therefore a convenient vehicle for demonstrating these descriptive outputs. Denoting the binary response by Y; the exposures by $Z_{1},\ldots ,Z_{m}$; and covariates by X, the BKMR model is $g(\mu_{i})=h(z_{i1},\ldots ,z_{\text{im}})+\beta X_{i},i=1,\ldots ,n$, where g is the probit link, so that $\Phi^{-1}(P(Y_{i}=1))=h(z_{i1},\ldots ,z_{\text{im}})+\beta X_{i}$, applied to $\mu_{i}=E(Y_{i})=P(Y_{i}=1)$ implemented via Gaussian data augmentation, h is a flexible nonparametric function of the exposures estimated via a Gaussian kernel, and β is a vector of covariate coefficients assumed to be linearly related to the latent outcome. Here, Z denotes the toxic-metal and essential-element exposures and h(·) captures their joint exposure–response relationship. We summarized the fitted model through posterior inclusion probabilities, univariate and bivariate exposure–response functions, single-exposure effects, and the overall mixture effect.

Because the link is probit rather than logit, h(z) is a shift in the latent normal index and is not a log-odds contrast. All changes in h reported in Section 3.9 are therefore changes on the latent probit scale; they are monotonically related to the probability of prevalent diabetes but are not themselves probability differences, and we describe them as such throughout.

## 3. Results

Interaction detection was evaluated against the three true pairwise interactions specified in Section 2.1.2. Thus, the interaction scenarios contained three true and 42 null candidate pairs, whereas the scenarios without interactions contained zero true and 45 null candidate pairs. Both enumerating methods were tuned using 10-fold cross-validation. All eight methods completed all 500 replicates across the eight simulation scenarios; therefore, the reported comparisons were not affected by differential fit failure.

### 3.1. Prediction Performance for Continuous Outcomes

#### 3.1.1. Mean Squared Error

**Figure 1.**
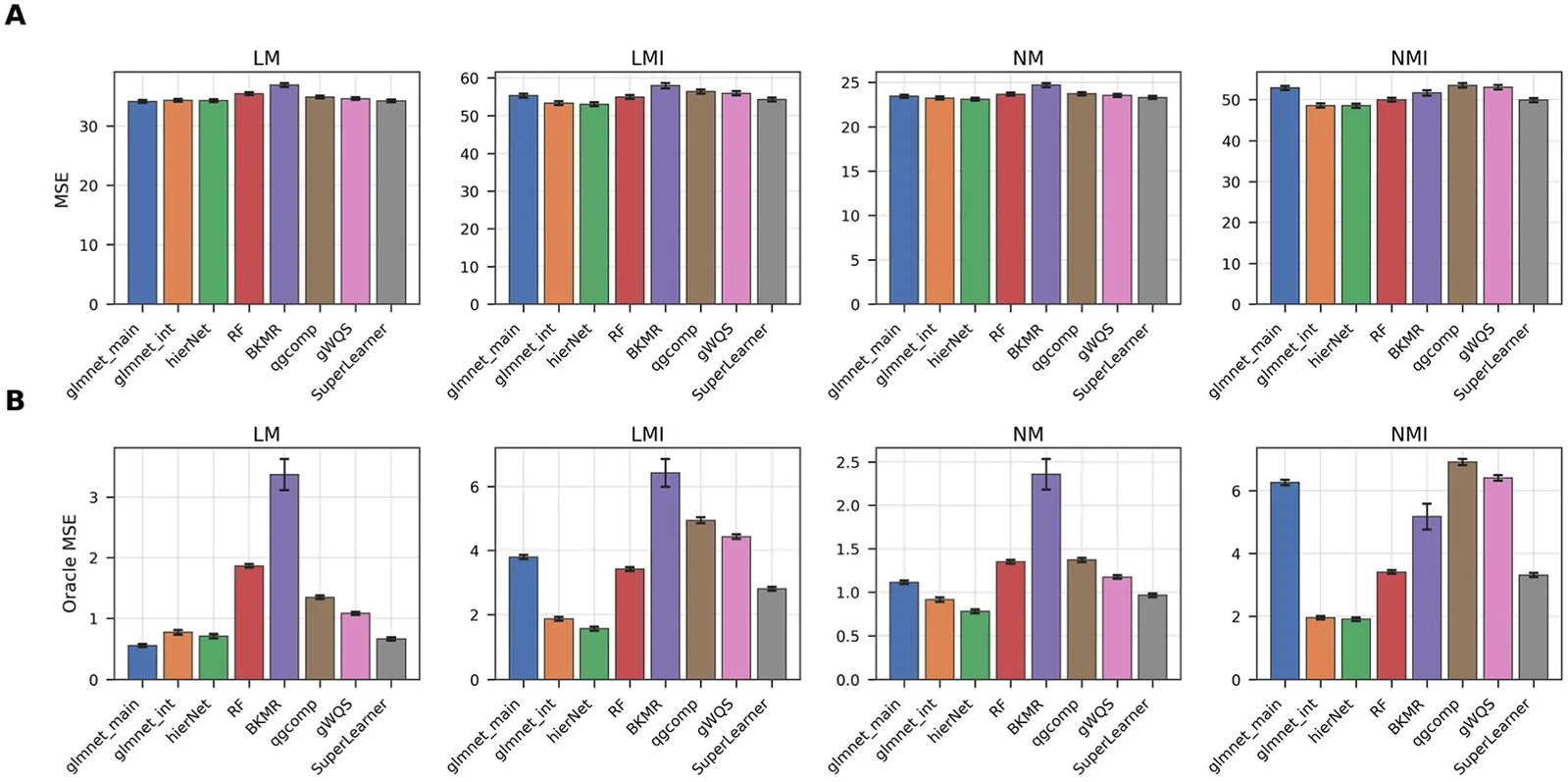
Predictive performance of the evaluated methods across four continuous-outcome scenarios. Panel (**A**) shows the mean squared error against the observed outcome and Panel (**B**) the mean squared error against the true conditional mean (oracle-referenced). Bars represent means across 500 replicates; error bars denote Monte Carlo standard errors rather than standard deviations, so that the resolution of the comparison is visible. LM = linear main effects; LMI = linear with interactions; NM = nonlinear main effects; NMI = nonlinear with interactions. Numeric values with standard deviations and Monte Carlo standard errors are given in Supplementary Table S4.

Figure 1 summarizes mean squared error across the four continuous-outcome scenarios, showing observed-outcome MSE (Figure 1A) and oracle-referenced MSE against the true conditional mean (Figure 1B).

##### Scenarios without interactions (LM and NM).

When the outcome involved only additive main effects, the methods performed comparably on the observed-outcome scale. In the LM scenario, MSE ranged from 34.06 (SD = 2.61, MCSE = 0.12) for glmnet_main to 36.85 (SD = 3.98) for BKMR, a spread of fewer than three MSE units; SuperLearner (34.16), hierNet (34.21), glmnet_int (34.27), gWQS (34.56), qgcomp (34.81) and Random Forests (35.37) fell in between. The NM scenario showed the same pattern: hierNet achieved the lowest MSE (23.07, SD = 1.95), followed by glmnet_int (23.21), SuperLearner (23.27), glmnet_main (23.42), gWQS (23.50), Random Forests (23.65), qgcomp (23.69) and BKMR (24.67). Because roughly 80% of outcome variance is irreducible noise by construction, these differences are compressed toward a common floor. The oracle-referenced metrics separate the methods far more sharply: MSE against the true conditional mean ranged from 0.56 (glmnet_main) to 3.37 (BKMR) in LM, a six-fold spread, and from 0.78 (hierNet) to 2.36 (BKMR) in NM. Thus, when exposures act through purely additive mechanisms, the choice of method has minimal consequence for observed predictive accuracy, while the ordering of estimation accuracy against the truth remains clear.

##### Scenarios with interactions (LMI and NMI).

Pairwise interactions produced wider performance gaps. (Outcome variance is larger in these scenarios, so MSE magnitudes are interpreted within scenario.) In LMI, hierNet achieved the lowest MSE (53.02, SD = 5.86, MCSE = 0.26) and glmnet_int was close behind (53.33, SD = 5.89), followed by SuperLearner (54.28), Random Forests (54.91), glmnet_main (55.27), gWQS (55.90), qgcomp (56.38) and BKMR (57.94). In NMI, the same two methods led (hierNet: 48.49, glmnet_int: 48.55), followed by SuperLearner (49.88), Random Forests (49.96), BKMR (51.66), glmnet_main (52.85), gWQS (53.03) and qgcomp (53.53). The oracle-referenced metric makes the cost of ignoring interactions explicit. In LMI, oracle MSE was 1.57 for hierNet and 1.87 for glmnet_int against 3.80 for glmnet_main, 4.43 for gWQS and 4.94 for qgcomp, so the additive-only methods incurred roughly two to three times the estimation error of the interaction-aware ones. In NMI, the gap was larger still: 1.91 and 1.95 for hierNet and glmnet_int against 6.27, 6.41 and 6.92 for glmnet_main, gWQS and qgcomp. SuperLearner occupied an intermediate position in both scenarios (2.81 and 3.31), consistent with a library that contains a flexible learner (SL.ranger) alongside additive ones but no explicit interaction terms; it should not be described as an additive-only method.

##### Paired between-method comparisons.

Because all methods were applied to the same simulated datasets, between-method differences can be computed within replicates, which removes between-dataset variation and gives intervals far narrower than the marginal standard deviations would suggest. Taking glmnet_int as the reference, the paired difference in oracle MSE in LMI was −0.30 for hierNet (95% CI: −0.35 to −0.26), +0.94 for SuperLearner (0.88 to 0.99), +1.55 for Random Forests (1.49 to 1.60), +1.92 for glmnet_main (1.86 to 1.98), +2.56 for gWQS (2.48 to 2.63), +3.06 for qgcomp (2.98 to 3.15) and +4.55 for BKMR (4.12 to 4.98), all with *p* < 0.001. In NMI, the corresponding differences were −0.05 for hierNet (−0.08 to −0.01, *p* = 0.011), +1.36 for SuperLearner, +1.45 for Random Forests, +3.22 for BKMR, +4.32 for glmnet_main, +4.46 for gWQS and +4.96 for qgcomp. hierNet is therefore reliably but only slightly better than glmnet_int, while the separation between the interaction-aware and additive-only methods is an order of magnitude larger. Full paired comparisons are given in Supplementary Table S8.

#### 3.1.2. Pearson Correlation

**Figure 2.**
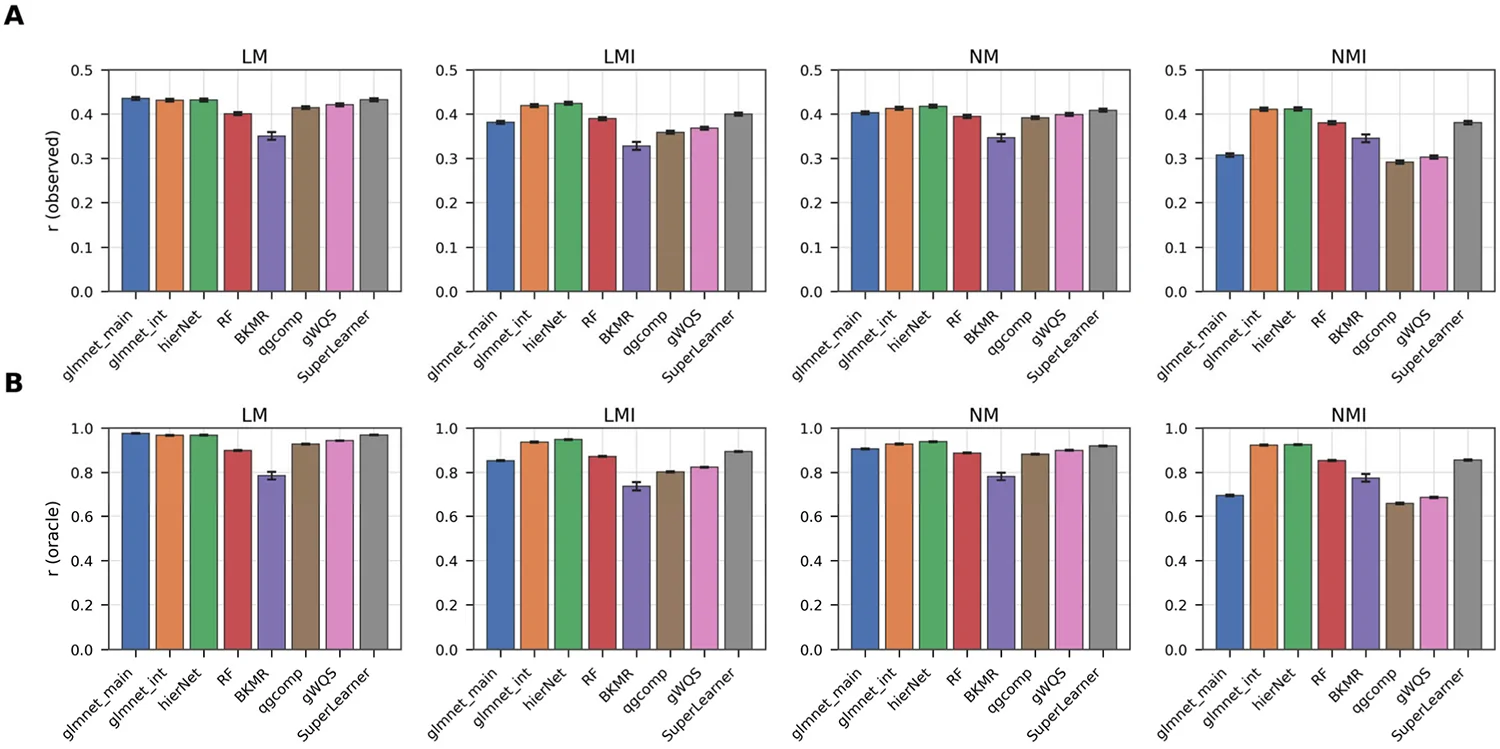
Pearson correlation for the evaluated methods across four continuous-outcome scenarios, (**A**) Pearson correlation between the observed outcome and model predictions. (**B**) Pearson correlation between the true conditional mean (oracle) and model predictions. Values represent means across 500 replicates with Monte Carlo standard errors as error bars. LM = linear main effects; LMI = linear with interactions; NM = nonlinear main effects; NMI = nonlinear with interactions. These are descriptive summaries; paired between-method differences with Monte Carlo intervals are given in Supplementary Table S8.

Pearson correlation results across the four continuous-outcome scenarios are summarized in Figure 2, with correlations against the observed outcome shown in Figure 2A and oracle-referenced correlations against the true conditional mean shown in Figure 2B.

##### Scenarios without interactions.

In LM, observed correlations ranged narrowly from 0.351 (BKMR) to 0.435 (glmnet_main), with SuperLearner (0.433), hierNet (0.432), glmnet_int (0.431), gWQS (0.421), qgcomp (0.415) and Random Forests (0.401) in between. In NM, the ordering was hierNet (0.418), glmnet_int (0.413), SuperLearner (0.409), glmnet_main (0.403), gWQS (0.399), Random Forests (0.395), qgcomp (0.392) and BKMR (0.347). The ceiling imposed by the noise level compresses these values; correlation against the true conditional mean separates the methods much more clearly, from 0.976 (glmnet_main) to 0.785 (BKMR) in LM and from 0.939 (hierNet) to 0.781 (BKMR) in NM.

##### Scenarios with interactions.

Interactions produced clear divergence. In LMI, hierNet (0.425) and glmnet_int (0.419) led, followed by SuperLearner (0.400), Random Forests (0.390), glmnet_main (0.381), gWQS (0.368), qgcomp (0.359) and BKMR (0.328). Against the true conditional mean, the same ordering holds with much greater separation: 0.948 and 0.937 for hierNet and glmnet_int, 0.894 for SuperLearner, 0.872 for Random Forests, 0.853 for glmnet_main, 0.823 for gWQS, 0.802 for qgcomp and 0.736 for BKMR at the 2000-iteration budget. NMI showed the same structure, with oracle correlations of 0.925 (hierNet), 0.924 (glmnet_int), 0.856 (SuperLearner), 0.854 (Random Forests), 0.775 (BKMR), 0.694 (glmnet_main), 0.685 (gWQS) and 0.658 (qgcomp). The additive-only methods lose roughly a quarter of the attainable correlation when interactions are present, whereas the interaction-aware methods lose almost none.

### 3.2. Interaction Detection for Continuous Outcomes

**Figure 3.**
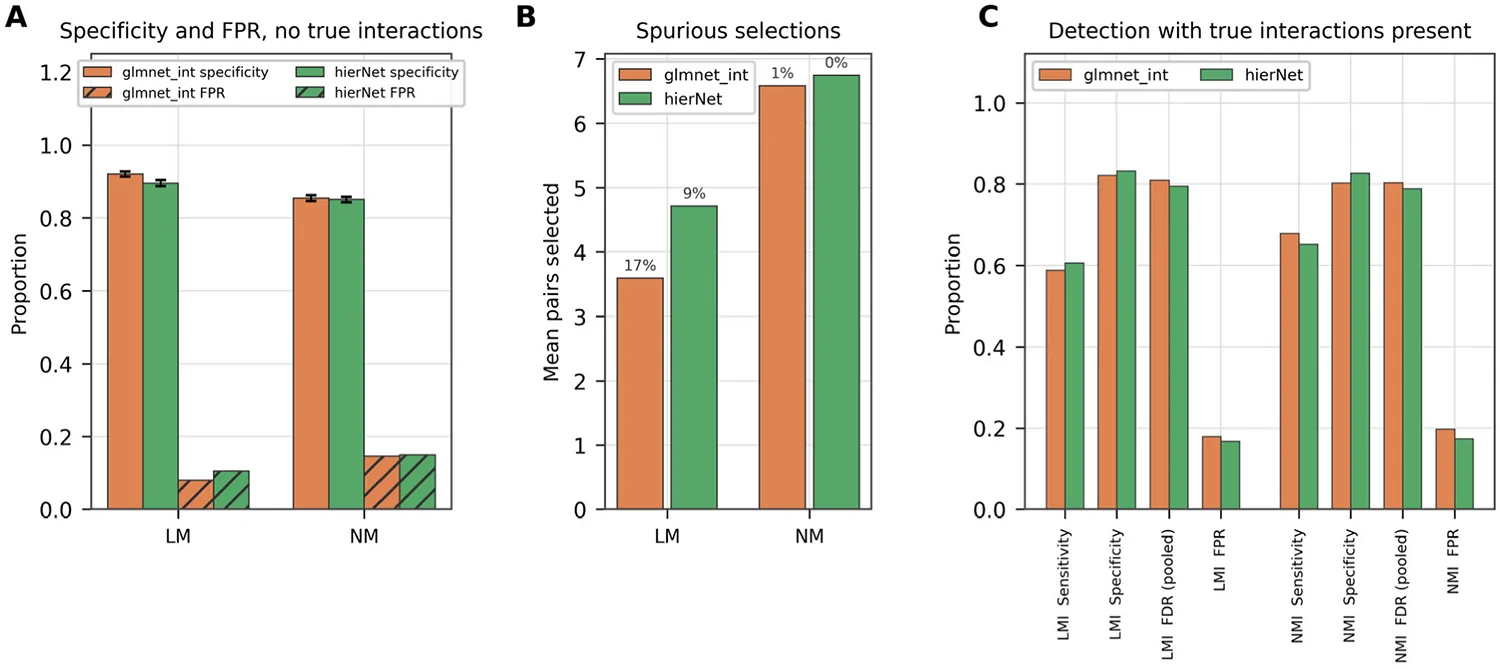
Interaction-detection performance of glmnet_int and hierNet across four continuous-outcome scenarios, with error bars showing Monte Carlo standard errors. Panel (**A**) shows specificity and the false-positive rate in the null scenarios (LM, NM) and Panel (**B**) the mean number of pairs selected in those scenarios, annotated with the percentage of replicates selecting no pair at all; sensitivity is undefined and the false discovery rate is exactly 1 by construction whenever any pair is selected, so neither is plotted. Panel (**C**) shows sensitivity, specificity, the pooled false discovery rate, and the false-positive rate in the interaction scenarios (LMI, NMI). A lower false-positive rate and a higher specificity indicate better false-positive control; higher sensitivity indicates better recovery of true interactions.

Only glmnet_int and hierNet return an explicit list of selected pairs and are therefore evaluated on sensitivity, specificity, false discovery rate and false-positive rate (Figure 3). Ranking-based detection, which additionally covers BKMR and Random Forests, is reported in Section 3.8. Both enumerating methods completed in all 500 replicates in every scenario, for both outcome types.

#### Scenarios without true interactions (LM and NM).

With no true interactions present, sensitivity is undefined, and, as set out in Section 2.3.2, the pooled false discovery rate is exactly 1 whenever any pair is selected. The informative summaries are therefore specificity, the false-positive rate, the number of pairs selected, and the proportion of replicates selecting nothing. In LM, glmnet_int achieved specificity 0.920 (SD = 0.081) and hierNet 0.895 (SD = 0.095), corresponding to false-positive rates of 0.080 and 0.105 and to a mean of 3.59 and 4.71 spuriously selected pairs per replicate; 17.2% of glmnet_int replicates and 8.8% of hierNet replicates selected no pair at all. In NM, both methods selected considerably more, with specificities of 0.854 and 0.850, false-positive rates of 0.146 and 0.150, means of 6.58 and 6.74 pairs, and almost no replicates selecting nothing (1.0% and 0.2%). Nonlinear main effects therefore induce substantially more spurious interaction selection than linear ones, which is a practically important failure mode: an analyst who has not modeled a nonlinear main effect may recover it as a spurious interaction.

#### Scenarios with true interactions (LMI and NMI).

The two methods perform comparably. In LMI, glmnet_int recovered 58.9% of true interactions (sensitivity: 0.589, SD = 0.289, MCSE = 0.013) and hierNet 60.6% (SD = 0.273, MCSE = 0.012), with specificities of 0.821 and 0.833 and false-positive rates of 0.179 and 0.167. In NMI, glmnet_int recovered 67.8% and hierNet 65.1%, with specificities of 0.802 and 0.826. The paired sensitivity difference (hierNet minus glmnet_int) was 0.017 in LMI (95% CI: −0.001 to 0.036, *p* = 0.069) and −0.027 in NMI (−0.044 to −0.009, *p* = 0.003). Neither method holds a sensitivity advantage: the two differ by less than three percentage points in either direction, and the direction of the difference is not consistent across scenarios. Where they differ reliably is in the false discovery burden, and there the advantage belongs to hierNet: the paired difference in per-replicate false discovery proportion was −0.019 in LMI (−0.030 to −0.008, *p* = 0.0007) and −0.021 in NMI (−0.030 to −0.012, *p* < 0.001), consistent with its selecting fewer pairs overall (8.85 versus 9.28 in LMI, 9.24 versus 10.34 in NMI). For continuous outcomes, the heredity constraint therefore buys a modest reduction in false discoveries at no measurable cost in sensitivity, including in a design where one of the three true interactions, (X5, X6), violates strong heredity by construction.

#### False discovery burden.

The realized false discovery burden is high for both methods. Pooled across replicates, the false discovery rate was 0.810 for glmnet_int and 0.795 for hierNet in LMI, and 0.803 and 0.789 in NMI; the corresponding means of the per-replicate false discovery proportion, conditioning on replicates that selected at least one pair, were 0.786, 0.767, 0.784 and 0.763. The two conventions differ by only 0.02 to 0.03, so the choice between them does not materially change the conclusion: with three true pairs among 45 candidates, roughly four in five selected pairs are false. No replicate in either interaction scenario failed to select at least one pair. As required by the algebraic identity between the pooled false discovery rate, mean sensitivity and mean false-positive rate, the verification gap was at most 1.1 × 10^−16^ in every scenario (Supplementary Table S6c). This burden is a property of the detection problem at this signal level rather than of either method, and it is the single most consequential practical finding of the benchmark: an interaction recovered by either method in a single dataset of this size should be treated as a hypothesis requiring replication, not as an established effect.

### 3.3. Classification Performance for Binary Outcomes

#### Scenarios without interactions (Logit and NLogit).

**Figure 4.**
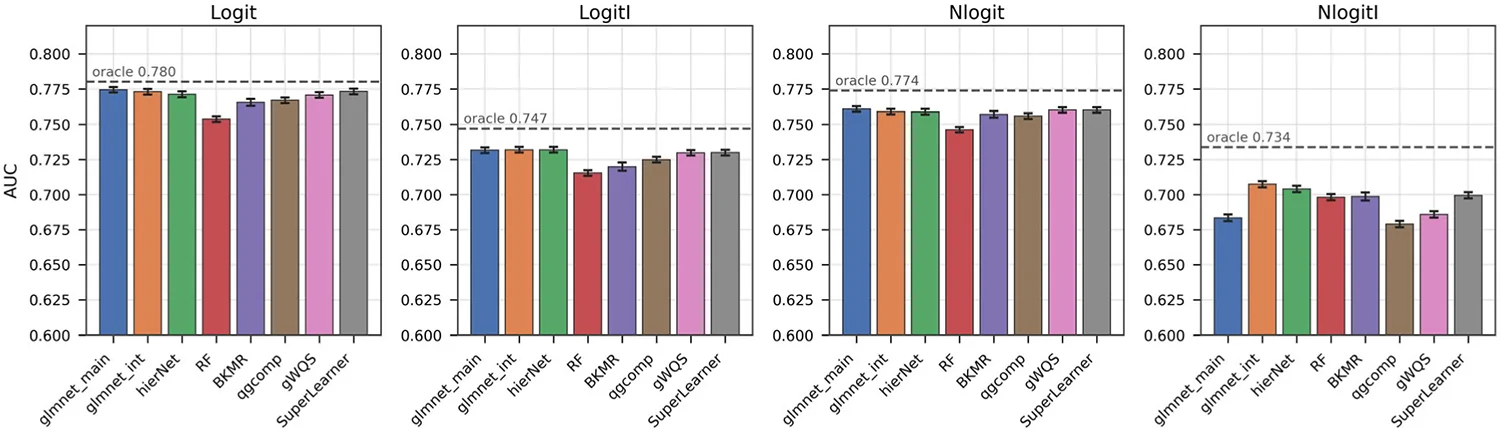
Area under the ROC curve results for the evaluated methods across four binary-outcome scenarios. Values represent mean AUCs across 500 replicates; the dashed horizontal line in each panel marks the Bayes-optimal AUC computed from the true generating probabilities (0.780, 0.747, 0.774 and 0.734 for Logit, LogitI, NLogit and NLogitI). All eight methods are shown; BKMR was fitted with est.h = TRUE, as described in Section 2.2.4. Logit and NLogit = logistic main effects (linear and nonlinear); LogitI and NLogitI = logistic with interactions.

All methods achieved strong, comparable AUCs (Figure 4). In Logit, glmnet_main was highest (0.774, SD = 0.022, MCSE = 0.001), followed by SuperLearner (0.773), glmnet_int (0.773), hierNet (0.771), gWQS (0.771), qgcomp (0.767), BKMR (0.765) and Random Forests (0.754). In NLogit, the ordering was similar (glmnet_main: 0.761, SuperLearner: 0.760, gWQS: 0.760, glmnet_int: 0.759, hierNet: 0.759, BKMR: 0.757, qgcomp: 0.756, Random Forests: 0.746). The oracle-referenced ceiling puts these values in context: the Bayes-optimal AUC obtainable from the true linear predictor is 0.780 in Logit and 0.774 in NLogit. The best-performing method is therefore within 0.006 and 0.013 of the theoretical maximum, while the weakest, Random Forests, falls 0.026 and 0.028 short of it. Little room remains for any method to distinguish itself in these additive binary scenarios, so the near-equivalence is largely a property of the design and should not be read as evidence that method choice is immaterial in settings with a stronger signal. For reference, the generating models correspond to a Tjur coefficient of discrimination of 0.235 and a McFadden pseudo-R^2^ of 0.187 in Logit, and 0.230 and 0.181 in NLogit, at a prevalence of 0.48 to 0.50.

#### Scenarios with interactions (LogitI and NLogitI).

Interactions reduced discrimination for all methods and separated them modestly. In LogitI, glmnet_int and hierNet were indistinguishable (0.7319 and 0.7319; paired difference: −0.00003, 95% CI: −0.0008 to 0.0008, *p* = 0.97), with glmnet_main (0.732), SuperLearner (0.730), gWQS (0.730), qgcomp (0.725), BKMR (0.720) and Random Forests (0.715) following, against an oracle ceiling of 0.747. In NLogitI, the separation was larger and in the expected direction: glmnet_int: 0.707, hierNet: 0.704, SuperLearner: 0.700, BKMR: 0.699, Random Forests: 0.698, gWQS: 0.686, glmnet_main: 0.684 and qgcomp: 0.679, against an oracle ceiling of 0.734. The paired deficits relative to glmnet_int in NLogitI were −0.024 for glmnet_main, −0.028 for qgcomp and −0.022 for gWQS, all with intervals excluding zero, whereas hierNet, SuperLearner, BKMR and Random Forests were within 0.009 of the reference. The generating models correspond to a Tjur coefficient of 0.197 and a McFadden pseudo-R^2^ of 0.157 in LogitI, and 0.184 and 0.147 in NLogitI. The additive penalty is thus present but smaller on the AUC scale than on the continuous prediction scale, because a binary outcome carries less information per observation and because AUC is a rank statistic that is relatively insensitive to the portion of the response surface the additive models misrepresent.

#### Sampler configuration for binomial BKMR.

Under the default kmbayes configuration with family = ‘binomial’ and componentwise variable selection, the sampler terminated with a non-positive-definite augmented kernel matrix in every attempted fit at *n* = 500. A diagnostic grid over four sampler configurations, three datasets per scenario and all four binary scenarios (Supplementary Table S13b) identified est.h = TRUE, which samples the exposure–response surface explicitly rather than marginalizing it out, as the configuration under which every fit completed. All BKMR results reported here use that configuration for both outcome families, so the sampler is identical across outcome types. The failure is therefore a property of the default sampling scheme in bkmr 0.2.2 for this design rather than an obstacle to fitting BKMR to binary outcomes, and it is reported because it is a practical trap for applied users fitting binomial BKMR to mixtures of this dimension.

### 3.4. Interaction Detection for Binary Outcomes

**Figure 5.**
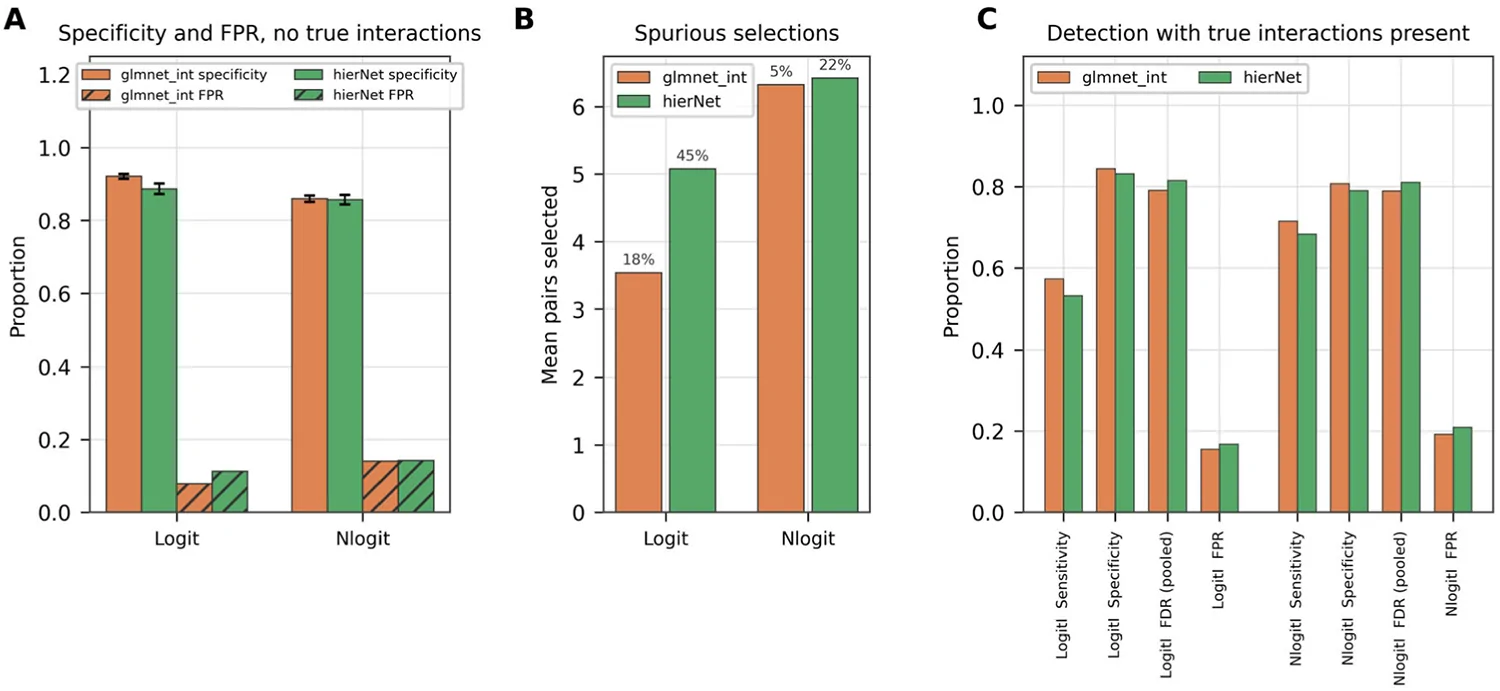
Interaction-detection performance of glmnet_int and hierNet across four binary-outcome scenarios, with error bars showing Monte Carlo standard errors. Panel (**A**) shows specificity and the false-positive rate in the null scenarios (Logit, NLogit) and Panel (**B**) the mean number of pairs selected in those scenarios, annotated with the percentage of replicates selecting no pair at all. Panel (**C**) shows sensitivity, specificity, the pooled false discovery rate, and the false-positive rate in the interaction scenarios (LogitI, NLogitI). Both methods are tuned by 10-fold cross-validation on the logistic solution path. As in Figure 3, sensitivity and the false discovery rate are omitted for the null scenarios, where the former is undefined and the latter is exactly 1 by construction. Logit and NLogit = logistic main-effects scenarios with no true interactions; LogitI and NLogitI = logistic scenarios with interactions.

For binary outcomes, explicit detection is reported for glmnet_int and hierNet, as for continuous outcomes (Figure 5). In hierNet 1.9, hierNet.cv accepts a logistic solution path, so λ is chosen by 10-fold cross-validation for binary as well as continuous outcomes, and the two enumerating methods are tuned identically throughout. Ranking-based detection for binary outcomes is reported in Section 3.8.

#### Scenarios without true interactions (Logit and NLogit).

As in the continuous case, sensitivity is undefined under the null, and the pooled false discovery rate is exactly 1 whenever any pair is selected. In Logit, glmnet_int achieved a specificity of 0.921 (SD = 0.076), with a false-positive rate of 0.079, a mean of 3.54 selected pairs per replicate, and 18.0% of replicates selecting nothing; hierNet achieved a specificity of 0.887, with a false-positive rate of 0.113, a mean of 5.08 pairs, and 45.2% of replicates selecting nothing. In NLogit, specificity fell to 0.860 for glmnet_int and 0.857 for hierNet, with false-positive rates of 0.140 and 0.143, means of 6.32 and 6.42 selected pairs, and 5.0% and 21.6% of replicates selecting nothing. hierNet’s selection behavior under the binary null is markedly more dispersed than glmnet_int’s: it selects nothing far more often, but selects more pairs when it selects at all.

#### Scenarios with true interactions (LogitI and NLogitI).

In LogitI, glmnet_int recovered 57.3% of true interactions (sensitivity: 0.573, SD = 0.279, MCSE = 0.013) and hierNet 53.2% (SD = 0.324, MCSE = 0.015), with specificities of 0.844 and 0.832, false-positive rates of 0.156 and 0.168, means of 8.26 and 8.65 pairs selected, and pooled false discovery rates of 0.792 and 0.816. In NLogitI, sensitivity rose to 0.715 for glmnet_int (SD = 0.260) and 0.683 for hierNet (SD = 0.278), with specificities of 0.808 and 0.790, means of 10.21 and 10.86 pairs, and pooled false discovery rates of 0.790 and 0.811. The paired sensitivity difference favored glmnet_int in both scenarios (−0.041, 95% CI: −0.067 to −0.016, *p* = 0.002 in LogitI; −0.032, −0.055 to −0.009, *p* = 0.006 in NLogitI), reversing the direction seen for continuous outcomes, while the paired difference in per-replicate false discovery proportion continued to favor hierNet (−0.028 and −0.027, both *p* < 0.005). Sensitivity for binary outcomes was modestly lower than for continuous outcomes in the linear scenario (0.573 versus 0.589 for glmnet_int) and higher in the nonlinear one (0.715 versus 0.678), and the false discovery burden was essentially identical across outcome types at roughly 0.79 to 0.82 pooled in every interaction scenario.

### 3.5. Computational Performance

**Figure 6.**
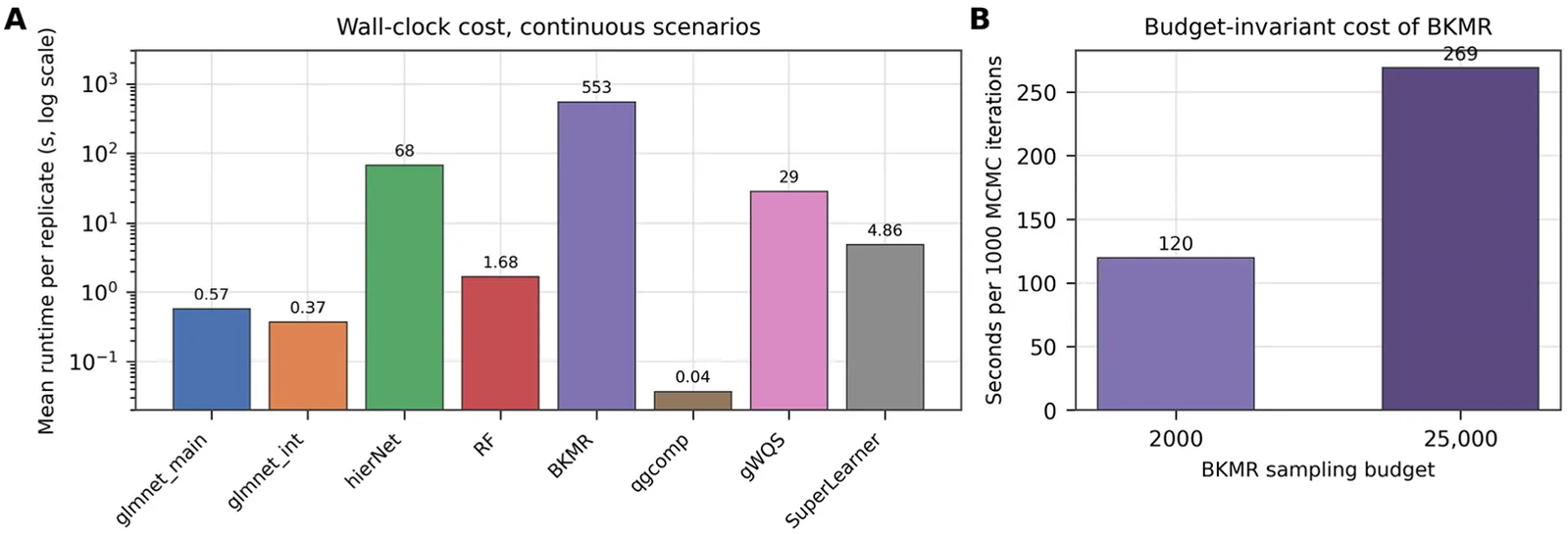
Computational cost of the evaluated methods across 500 replicates. Panel (**A**) shows mean wall-clock runtime per replicate in seconds on a logarithmic scale, summarized across the four continuous-outcome scenarios so that every method contributes to every scenario; binary-outcome runtimes are given in Supplementary Table S5. Runtimes in Panel (**A**) were recorded with all eight methods running concurrently across 64 workers. Panel (**B**) shows, for BKMR alone, mean seconds per 1000 MCMC iterations at the 2000- and 25,000-iteration budgets in the paired budget experiment of Section 3.7, which was executed as a separate run under lighter contention; this per-iteration cost is the budget-invariant summary, and the total runtime of a BKMR analysis is this quantity multiplied by the iteration count the analyst selects.

Mean runtime per replicate varied by four orders of magnitude. The values below are means across the four continuous scenarios, matching Figure 6; binary-outcome runtimes are given in Supplementary Table S5. qgcomp was fastest at 0.04 s, followed by glmnet_int at 0.37 s and glmnet_main at 0.57 s, making the penalized methods highly practical at scale. Random Forests were intermediate at 1.68 s and SuperLearner at 4.86 s. gWQS required 28.5 s, reflecting its bootstrap resampling, and hierNet 67.6 s, reflecting 10-fold cross-validation over the full solution path; for binary outcomes, hierNet rose to 242 s because the logistic solution path is more expensive to cross-validate. BKMR required 553 s per replicate for continuous outcomes and 689 s for binary outcomes at the 2000-iteration budget used in the primary benchmark. These wall-clock figures were obtained with all eight methods running concurrently across 64 workers, whereas the paired budget experiment of Section 3.7 ran BKMR alone under lighter contention and recorded 236 to 245 s per replicate at 2000 iterations (118 to 123 s per 1000) against 6265 to 7034 s at 25,000 iterations (251 to 281 s per 1000). Absolute wall-clock times are therefore not comparable between the two runs. Because runtime for an MCMC method is a linear function of an analyst-chosen iteration count rather than a property of the algorithm, any ratio between BKMR and a non-iterative method is a statement about the budget chosen: at the 25,000 iterations at which diagnostics became acceptable, BKMR is roughly four orders of magnitude slower than glmnet_int, and at 2000 iterations roughly three, but neither figure is a property of the algorithm on its own. What is a property of the algorithm is the cost per iteration together with the number of iterations needed to reach acceptable diagnostics, which in this design was on the order of 25,000. The measured computational cost of the instrumented stages is given in Supplementary Table S18.

### 3.6. Sensitivity Analyses of the Simulation Design

#### Signal strength.

Raising the target R^2^ from 0.20 to 0.30 and 0.40 generally improved absolute performance, most clearly for the interaction-capable methods, although several additive-only methods were non-monotonic across the three levels (for example, gWQS in LMI at 4.34, 4.06 and 4.14, and qgcomp in NMI at 7.27, 6.39 and 6.47) and exact ranks shifted among the middle of the field. The qualitative conclusion was preserved: hierNet and glmnet_int remained the strongest performers at every level and the disadvantage of the additive-only models persisted. In LMI, oracle MSE fell from 1.86 to 0.84 for glmnet_int and from 1.41 to 0.73 for hierNet, while the additive-only methods improved far less (glmnet_main: 3.80 to 3.33, qgcomp: 5.03 to 4.22, gWQS: 4.34 to 4.14). The relative penalty for ignoring interactions therefore grows as signal strength increases, because a stronger interaction signal is a larger part of what an additive model cannot represent. BKMR improved most in relative terms (5.14 to 1.08 in LMI), consistent with a flexible method that is limited by sampling noise rather than by model form. Because the binary data-generating process is calibrated through the latent-scale standard deviation of the linear predictor rather than through an error variance, the target R^2^ does not govern the binary scenarios; binary signal strength is varied separately in Section 2.4 and reported on the Tjur and McFadden scales in Supplementary Table S2b, with method-specific AUC results reported in Supplementary Table S16b, where the AUC in LogitI rises from 0.649 to 0.792 for glmnet_int across the three signal levels.

#### Sample size.

Varying the training sample size across 250, 500 and 1000 preserved the separation between the interaction-aware and the additive-only methods on the continuous oracle-referenced metrics while narrowing the absolute gaps, although individual ranks varied between settings. On binary AUC the differences were modest and in some settings absent: at *n* = 250 in LogitI, glmnet_main reached 0.721 against 0.718 for glmnet_int and 0.719 for hierNet. In LMI, oracle MSE for hierNet fell from 2.67 to 0.88 and for glmnet_int from 3.12 to 0.98, while qgcomp improved only from 6.17 to 4.19 and gWQS from 5.12 to 4.00: additional data cannot repair a misspecified mean structure, so the additive-only methods converge to a bias floor while the interaction-aware methods continue to improve. SuperLearner improved steadily (3.78 to 2.06), tracking the interaction-aware methods at a distance. Three method-by-scenario combinations were non-monotonic in sample size: BKMR in LMI (4.51, 3.43, 8.29) and NMI (3.77, 3.77, 5.39), and gWQS in NMI (6.67, 7.07, 6.28). The BKMR pattern is expected at a fixed 2000-iteration budget: a larger dataset requires more iterations to explore the same posterior, so holding the budget fixed while increasing *n* degrades rather than improves the fit. This is a further illustration of the budget effect documented in Section 3.7 rather than a property of the method. All eight methods were evaluated at every level, with six methods evaluated at 50 replicates and BKMR and gWQS at 15, so that no method was excluded from the generalizability assessment.

#### Exposure correlation.

Moving from the primary block structure (0.6 within, 0.1 between) to a low-correlation setting (0.3, 0.05) and a high-correlation setting (0.8, 0.3) changed absolute performance considerably but preserved the ranking of the interaction-aware methods. In LMI, oracle MSE for hierNet was 1.28 under low correlation, 1.61 at the primary setting and 1.65 under high correlation, and for glmnet_int the corresponding values were 1.49, 1.92 and 1.97. The additive-only methods degraded far more steeply under high correlation (glmnet_main: 2.26 to 5.62, qgcomp: 2.82 to 7.64, gWQS: 2.55 to 6.84), because collinearity among exposures makes an omitted interaction increasingly difficult to absorb into main-effect coefficients. The practical implication is that the advantage of interaction-aware modeling is largest precisely in the correlated mixtures that motivate this literature.

#### Pure-product interaction.

Replacing the interaction function with the pure multiplicative form g(Xk, Xl) = 0.6·Xk·Xl, removing the quadratic threshold component, left the substantive conclusions intact. In LMI_pure, oracle MSE was 1.35 for hierNet and 1.48 for glmnet_int against 2.77 for glmnet_main, 3.27 for gWQS and 3.59 for qgcomp, closely mirroring the primary specification. Detection sensitivity was 0.620 for hierNet and 0.613 for glmnet_int in LMI_pure against 0.606 and 0.589 in LMI, and pooled false discovery rates were 0.808 and 0.793 against 0.795 and 0.810. In the binary pure-product scenarios, the same pattern held, with glmnet_int retaining a small sensitivity advantage (0.567 versus 0.493 in LogitI_pure, 0.747 versus 0.667 in NLogitI_pure). The pooled false discovery ordering between the two enumerating methods did shift in two of the four scenarios: hierNet holds the lower pooled rate in LMI but glmnet_int does in LMI_pure, and the reverse occurs between LogitI and LogitI_pure. Neither method is uniformly superior under either specification, so the primary conclusion that there is no uniformly superior enumerating method does not depend on the composite interaction form, and hierNet is neither advantaged nor disadvantaged overall by the quadratic threshold term.

#### Detection tolerance.

Varying the tolerance used to declare a hierNet interaction parameter nonzero across exact zero and nonzero values from 1 × 10^−12^ to 1 × 10^−6^ changed nothing of substance. In LMI, the sensitivity was 0.653 at every tolerance and the mean number of pairs selected moved from 8.32 to 8.18 across nonzero tolerances from 1 × 10^−12^ to 1 × 10^−6^ and exact zero; in NMI, sensitivity moved from 0.673 to 0.667, and the mean from 9.98 to 9.86. This is as expected: the L1 penalty in hierNet sets interaction parameters to exact zero, so the cut-off is a floating-point tolerance for numerical zero rather than a statistical selection threshold, and the statistical tuning parameter is λ, chosen by cross-validation. Full results are in Supplementary Table S3.

### 3.7. BKMR Sampling Budget and Convergence

#### Convergence diagnostics.

**Figure 7.**
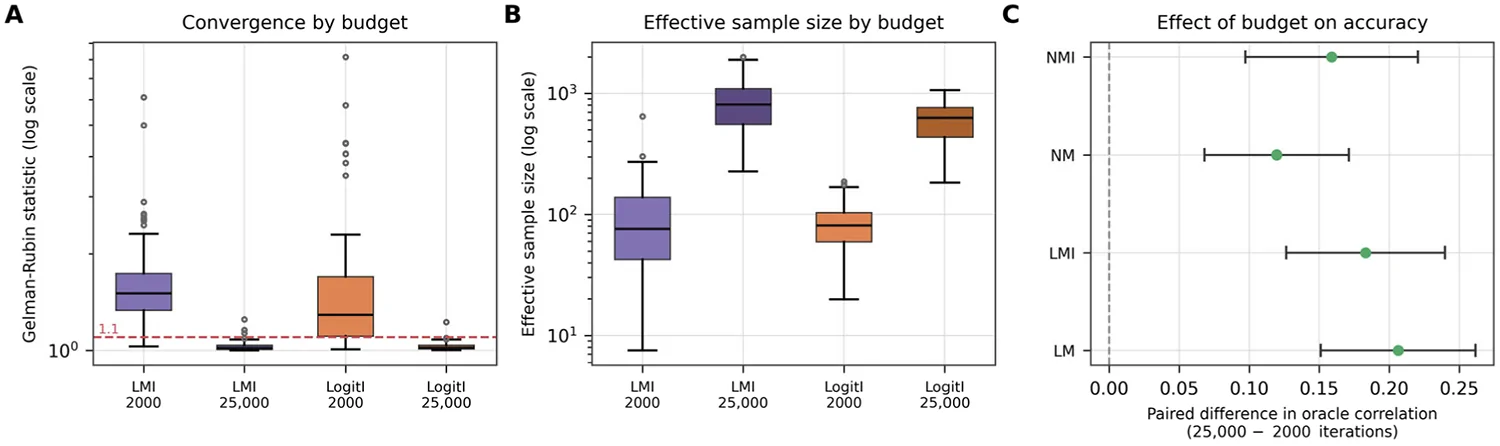
BKMR convergence and sampling budget. Panel (**A**) shows the distribution of the Gelman–Rubin statistic across the monitored parameters and five datasets at the 2000- and 25,000-iteration budgets, with a reference line at 1.1. Panel (**B**) shows the effective sample size on a logarithmic scale at the two budgets. Panel (**C**) shows paired within-replicate differences in oracle-referenced correlation between the two budgets for each continuous scenario, with 95% Monte Carlo intervals. Diagnostics were computed with coda from four independent chains started from different random initializations, on unthinned post-burn-in draws, for the continuous scenario LMI and the binary scenario LogitI.

Four independent chains were run from different random initializations on five datasets in the LMI scenario and five in the LogitI scenario at each budget, and the Gelman–Rubin statistic and effective sample size were computed with coda on the unthinned post-burn-in draws for the ten kernel bandwidth parameters; the variance component; and, for continuous outcomes, the residual variance (Figure 7A,B). At 2000 iterations the chains were not converged by any conventional standard: in LMI, 93.3% of the 60 monitored parameter-by-dataset combinations had a statistic above 1.1, the median was 1.50, the maximum 6.10, and effective sample sizes ranged from 7.5 to a median of 75.8. LogitI was similar, with 76.4% above 1.1, a median of 1.29, a maximum of 8.15 and a minimum effective sample size of 19.8. At 25,000 iterations the same diagnostics were acceptable: in LMI, the median statistic was 1.02 with a maximum of 1.25, only 5.0% exceeded 1.1, and the median effective sample size was 809 with a minimum of 227; in LogitI, the median was 1.02, the maximum 1.22, 1.8% exceeded 1.1, and the median effective sample size was 629 with a minimum of 183. Prediction results obtained at the shorter budget therefore reflect non-convergence rather than method performance.

#### Budget sensitivity.

BKMR was refitted at both budgets on 50 paired replicates per scenario, with the two budgets applied to identical datasets so that the comparison is paired within replicates (Figure 7C). Every metric improved at the longer budget. Mean oracle MSE fell by 3.11 units in LM (95% CI: 2.28 to 3.94), 4.30 in LMI (2.95 to 5.66), 1.22 in NM (0.69 to 1.76) and 3.36 in NMI (2.01 to 4.70), all with *p* < 3 × 10^−5^. Oracle-referenced correlation rose by 0.206, 0.183, 0.120 and 0.159 respectively, moving from a range of 0.741 to 0.833 at the short budget to 0.924 to 0.963 at the long one. In the binary scenarios, AUC rose by 0.007 to 0.015, with all four intervals excluding zero (*p* = 0.0002 to 0.013), a smaller absolute change because AUC is bounded and the additive information in a binary outcome is limited. In LMI, oracle MSE fell from 6.38 at 2000 iterations to 2.08 at 25,000, which places BKMR within the range of SuperLearner and Random Forests rather than last among the eight methods.

#### Interpretation.

Taken together, these results establish that the higher MSE, lower correlation and markedly greater replicate-to-replicate variability exhibited by BKMR in the primary benchmark are consequences of the sampling budget and not properties of the method. At a defensible budget, BKMR’s oracle correlation in LMI (0.924) is close to that of glmnet_int (0.937) and hierNet (0.948), a small fraction of the gap implied by the short-budget results. BKMR should therefore not be described as showing less stable prediction without reference to its sampling budget, and comparisons in the applied literature that report BKMR alongside penalized methods should state the iteration count and the convergence diagnostics achieved. The practical point is narrow and useful: a budget of 2000 iterations does not explore the posterior in a design of this size, and results obtained at such a budget should not be interpreted as characterizing the method. The corollary for cost is equally practical, since reaching an acceptable budget in this design required roughly 25 times the computation.

### 3.8. Ranking-Based Interaction Detection

#### Ranking performance.

**Figure 8.**
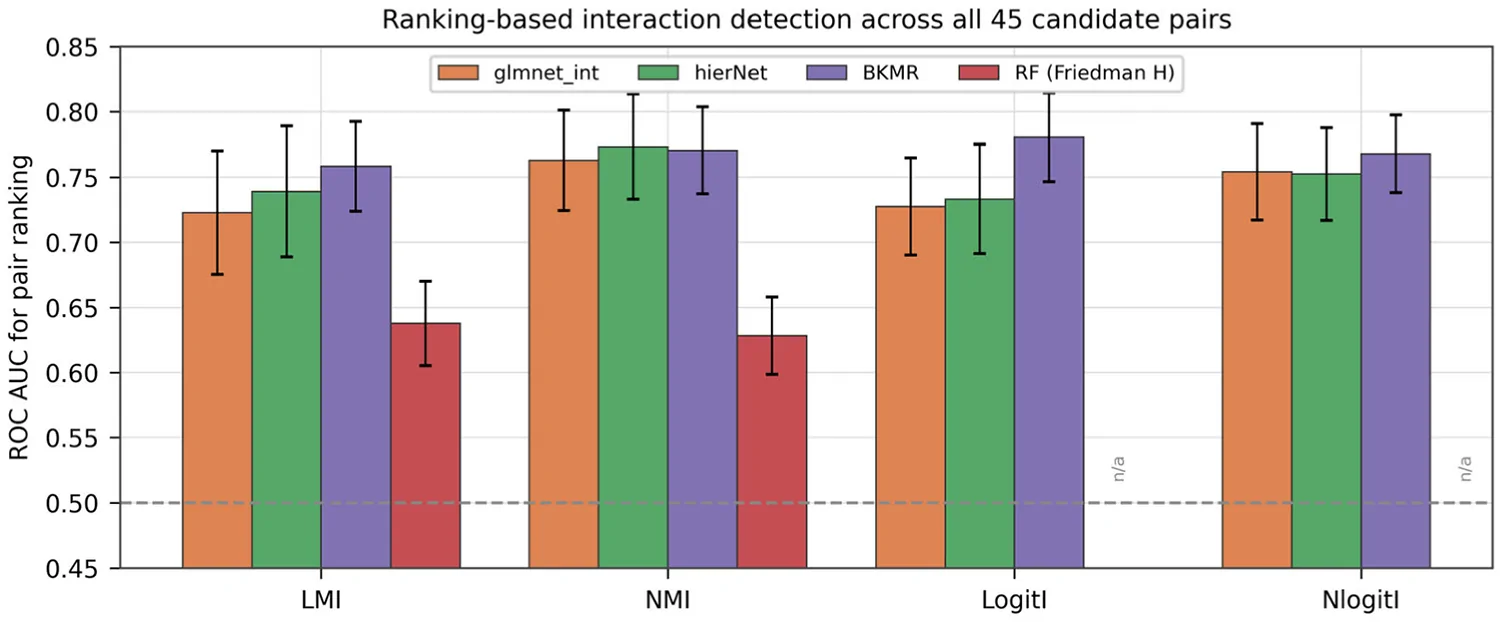
Ranking-based interaction detection. Area under the ROC curve results for the ranking of all 45 candidate pairs against the three true pairs in the four interaction scenarios. glmnet_int, hierNet, and BKMR fitted at 25,000 iterations, and Random Forests scored by the Friedman H-statistic are shown for LMI and NMI; the Random Forest statistic was not obtainable for the binary scenarios, so LogitI and NLogitI show the remaining three methods. Bars show means across 50 replicates with Monte Carlo standard errors, and the dashed line at 0.5 marks chance ranking.

Scoring all 45 candidate pairs by rank rather than by hard selection allows BKMR and Random Forests to be evaluated on interaction detection against the same ground truth used for the enumerating methods, using 50 replicates per scenario with BKMR fitted at 25,000 iterations (Figure 8). Under this scoring, BKMR was competitive with both penalized methods across all four interaction scenarios. In LMI, the area under the ROC curve for the pair ranking was 0.758 (MCSE 0.018) for BKMR, 0.739 (0.026) for hierNet and 0.723 (0.024) for glmnet_int; in NMI, it was 0.773 (0.021) for hierNet, 0.771 (0.017) for BKMR and 0.763 (0.020) for glmnet_int. For binary outcomes, BKMR achieved 0.781 in LogitI and 0.768 in NLogitI against 0.728 and 0.754 for glmnet_int and 0.733 and 0.753 for hierNet. On average precision, the penalized methods retained a modest advantage (LMI: 0.432 for hierNet, 0.410 for glmnet_int, 0.354 for BKMR), and recall among the top three ranked pairs was 0.347, 0.327 and 0.280 respectively, with the mean rank of a true pair between 10.8 and 12.3 out of 45 for all three methods. Random Forest interaction scores, computed as Friedman H-statistics, ranked substantially worse than those of the other three methods (ROC AUC: 0.638 in LMI and 0.628 in NMI, top-three recall 0.107 in both, mean true-pair rank: 20.2 to 22.4) and could not be obtained for the binary scenarios because the classification predictor returned no pairwise statistic; those cells are reported as unusable in Supplementary Table S7 rather than omitted.

#### Interpretation.

The practical implication is that excluding BKMR from detection comparisons, as is standard because it performs no hard selection, understates its capability. BKMR does not return a selected set of pairs, so it cannot be scored on sensitivity or false discovery rate, but the bivariate interaction summaries that the applied literature already uses as informal interaction diagnostics do carry information about which pairs are truly interacting, and by a ranking criterion that information is competitive with what the enumerating methods provide. The contrast with Random Forests is instructive: a general-purpose interaction statistic applied to a flexible black-box learner recovers the true pairs far less well than either a structured penalized model or a kernel machine designed for exposure mixtures. Equally, the absolute values put the whole detection exercise in perspective: an area under the curve of 0.72 to 0.78 and a mean true-pair rank near 11 out of 45 indicate that these methods perform well above chance but are far from reliable pair identification at this signal level.

#### Random Forests.

Random Forests do not return a pairwise interaction score natively, so they are scored here using the model-agnostic Friedman H-statistic. This places them on the same ranking footing as the other methods for continuous outcomes but not for binary ones, where the implementation returned no pairwise statistic for a classification forest. Extending model-agnostic interaction statistics to binary mixture outcomes is a natural direction for further work.

### 3.9. Applied Results: Metal Mixture and Diabetes

To illustrate how a toxic-metal and essential-element mixture relates to prevalent diabetes, a BKMR framework was applied to jointly model the six elements and the binary diabetes outcome.

#### 3.9.1. Posterior Inclusion Probabilities

Table 1 reports the posterior inclusion probability (PIP) for each element, the estimated probability that the element contributes meaningfully to variation in the outcome, with values near 1 indicating high importance. Lead, manganese, and iron each had a posterior inclusion probability of 1.000, the highest in the mixture under the fitted variable-selection model, followed by selenium (0.876). A posterior inclusion probability describes the posterior evidence that an exposure enters the kernel conditional on the other components and the covariates; it is not a measure of effect size, and high values should not be read as establishing that these elements dominate the exposure–response surface. Cadmium showed moderate importance (0.645), and mercury the lowest inclusion probability (0.260), indicating a more limited independent contribution once the full mixture is considered.

Formally, writing δ_m_ for the binary indicator that exposure, m, is included in the kernel, the posterior inclusion probability is PIP_m_ = Pr(δ_m_ = 1 ∣ data), estimated as the proportion of retained posterior draws in which δ_m_ = 1. All credible intervals reported for the applied model are equal-tailed 95% intervals, computed as the 2.5th and 97.5th percentiles of the retained posterior draws of the quantity concerned.

#### 3.9.2. Univariate Exposure–Response Functions

**Figure 9.**
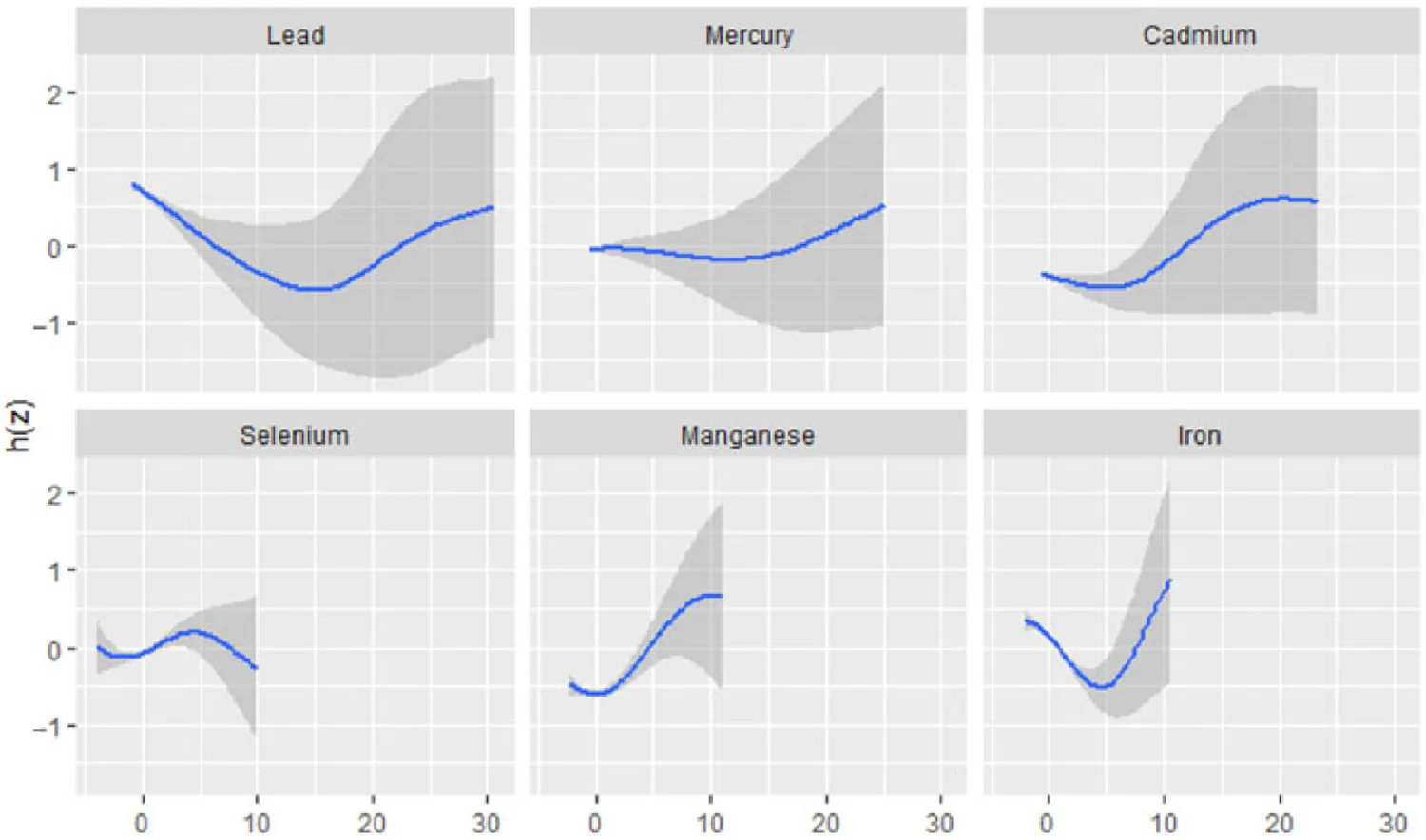
Estimated univariate exposure–response functions with 95% credible intervals (grey shaded bands) from the BKMR model, showing the association between each individual exposure and prevalent diabetes while all other exposures are held at their medians. Adjusted for age, ethnicity, sex, BMI, smoking, alcohol use, income, education, and physical activity.

Holding all other exposures at their medians, the individual exposure–response functions were predominantly nonlinear (Figure 9). Cadmium showed a positive, S-shaped association, with h(z) relatively flat at lower concentrations and rising across the mid-to-upper range. Manganese showed a positive, increasing association, rising from low to moderate concentrations before plateauing. Lead and iron each displayed U-shaped, non-monotonic relationships: h(z) declined from lower concentrations to a trough near the center of the observed range and then rose at higher concentrations. Mercury was essentially flat with a slight positive trend at the upper end, and selenium was near-flat across its comparatively narrow range, rising slightly at low-to-moderate levels before a gentle decline. Credible intervals widened toward the extremes of each exposure, where data were sparse.

#### 3.9.3. Bivariate Surfaces and Interaction

**Figure 10.**
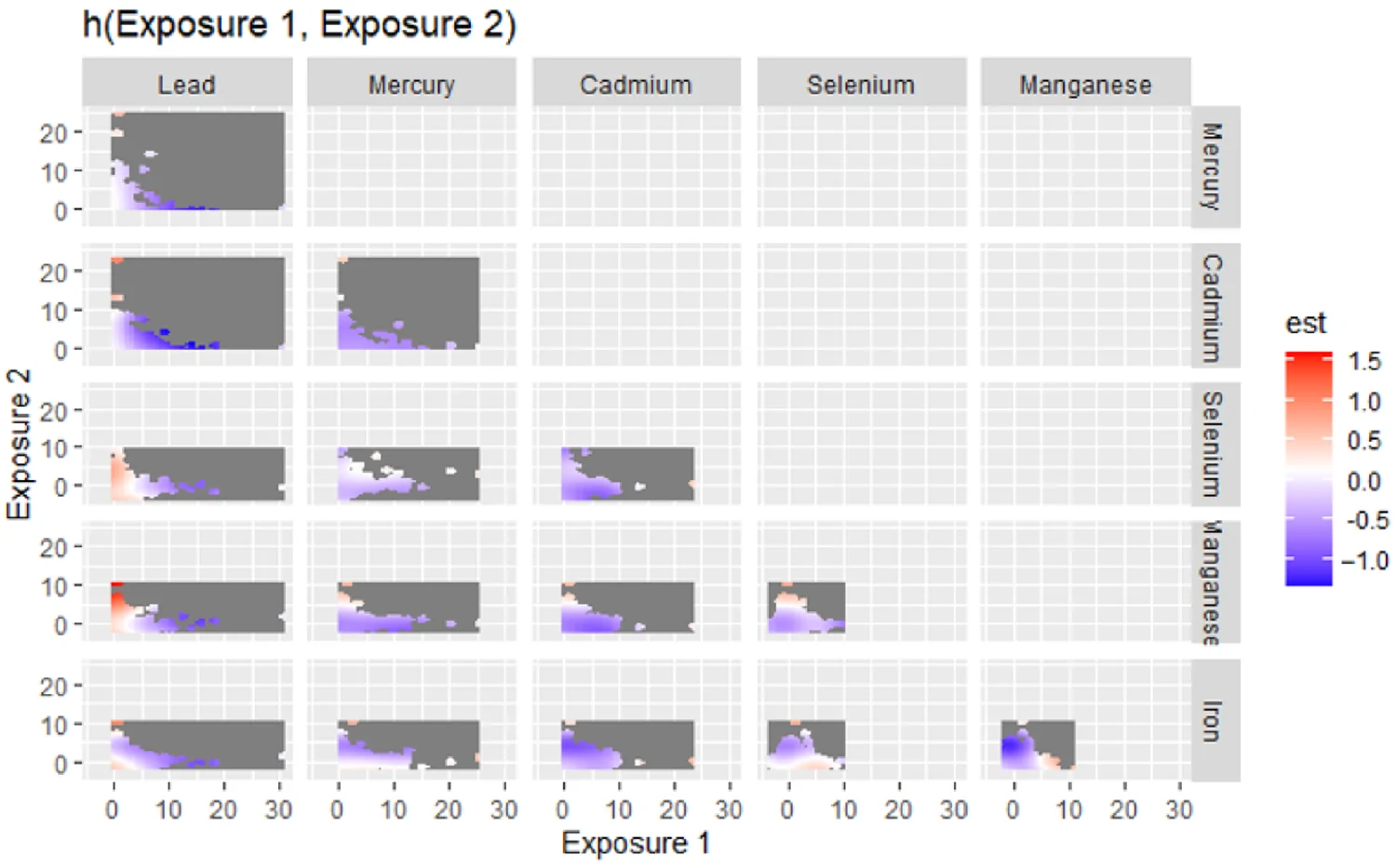
Estimated bivariate exposure–response surfaces showing the joint association of each element pair with prevalent diabetes while all other exposures are held at their medians. Color represents h(z) on the latent probit scale, from blue (lower estimated h(z)) to red (higher estimated h(z)); Exposure 1 varies along the horizontal axis and Exposure 2 along the vertical axis. Grey regions mark combinations of the two exposures that lie outside the observed joint range of the data, where the surface is not estimated; each pair is shown once, so the upper triangle of the grid is empty by design. Adjusted for age, ethnicity, sex, BMI, smoking, alcohol use, income, education, and physical activity.

**Figure 11.**
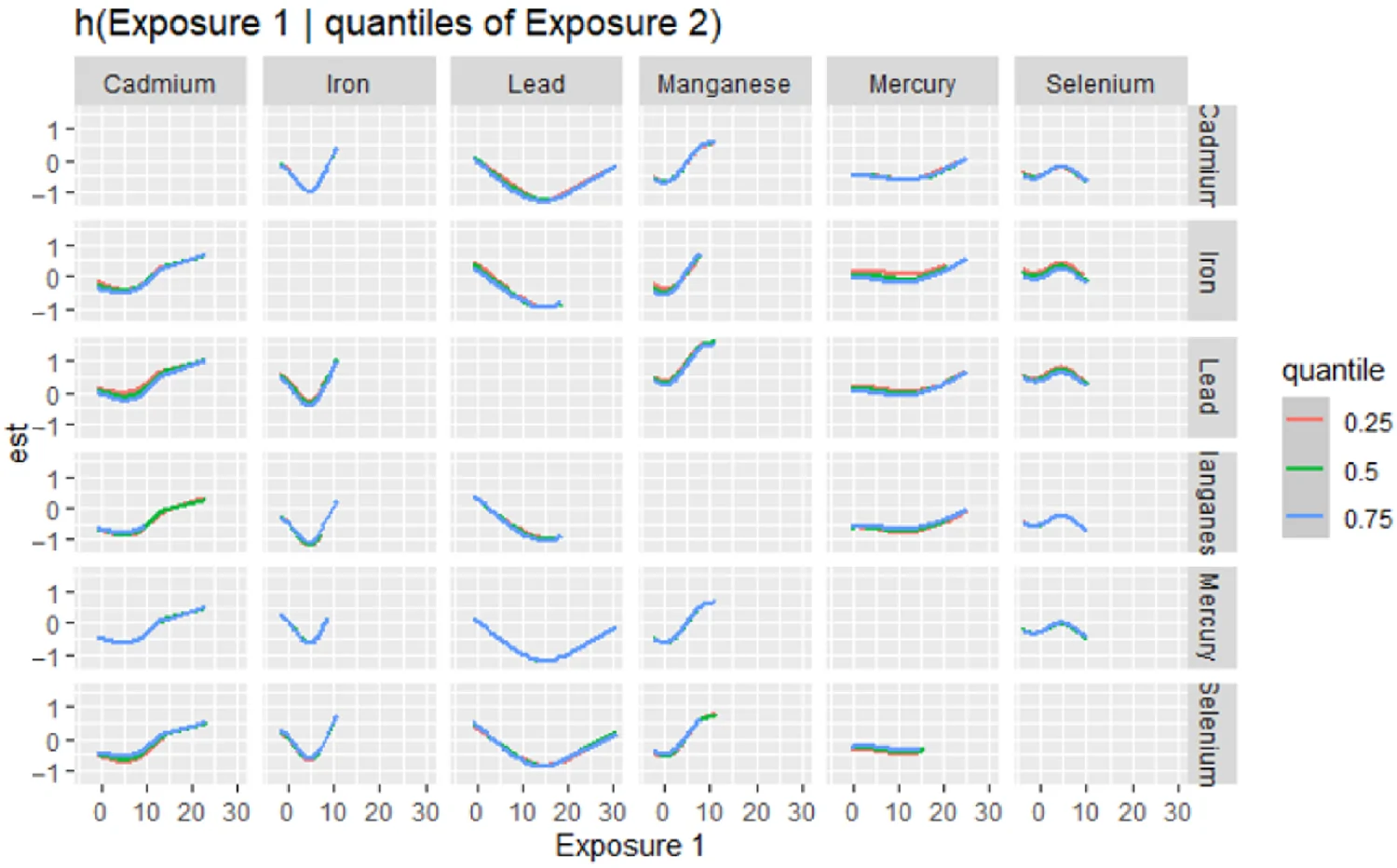
Bivariate exposure–response functions for each element (Exposure 1, x-axis) with a second element fixed at its 25th (red), 50th (green), and 75th (blue) percentiles. Closely overlapping curves indicate minimal effect modification. Adjusted for age, ethnicity, sex, BMI, smoking, alcohol use, income, education, and physical activity.

The bivariate exposure–response surfaces (Figure 10) were generally smooth, with the steepest gradients localized to the lead–manganese region of the joint surface; most pairs transitioned gradually, indicating limited evidence of strong pairwise interaction. This was reinforced by the cross-sectional plots in Figure 11, which show the exposure–response function for one element while a second element is fixed at its 25th, 50th, and 75th percentiles. For every pair, the three quantile curves were closely superimposed, indicating that the effect of one element changed little with the level of another, that is, little effect modification among the mixture components. The element-specific shapes in Figure 11 reproduce those in Figure 9: cadmium and manganese increasing, lead and iron U-shaped, and mercury and selenium near-flat.

#### 3.9.4. Single-Exposure Effects

**Figure 12.**
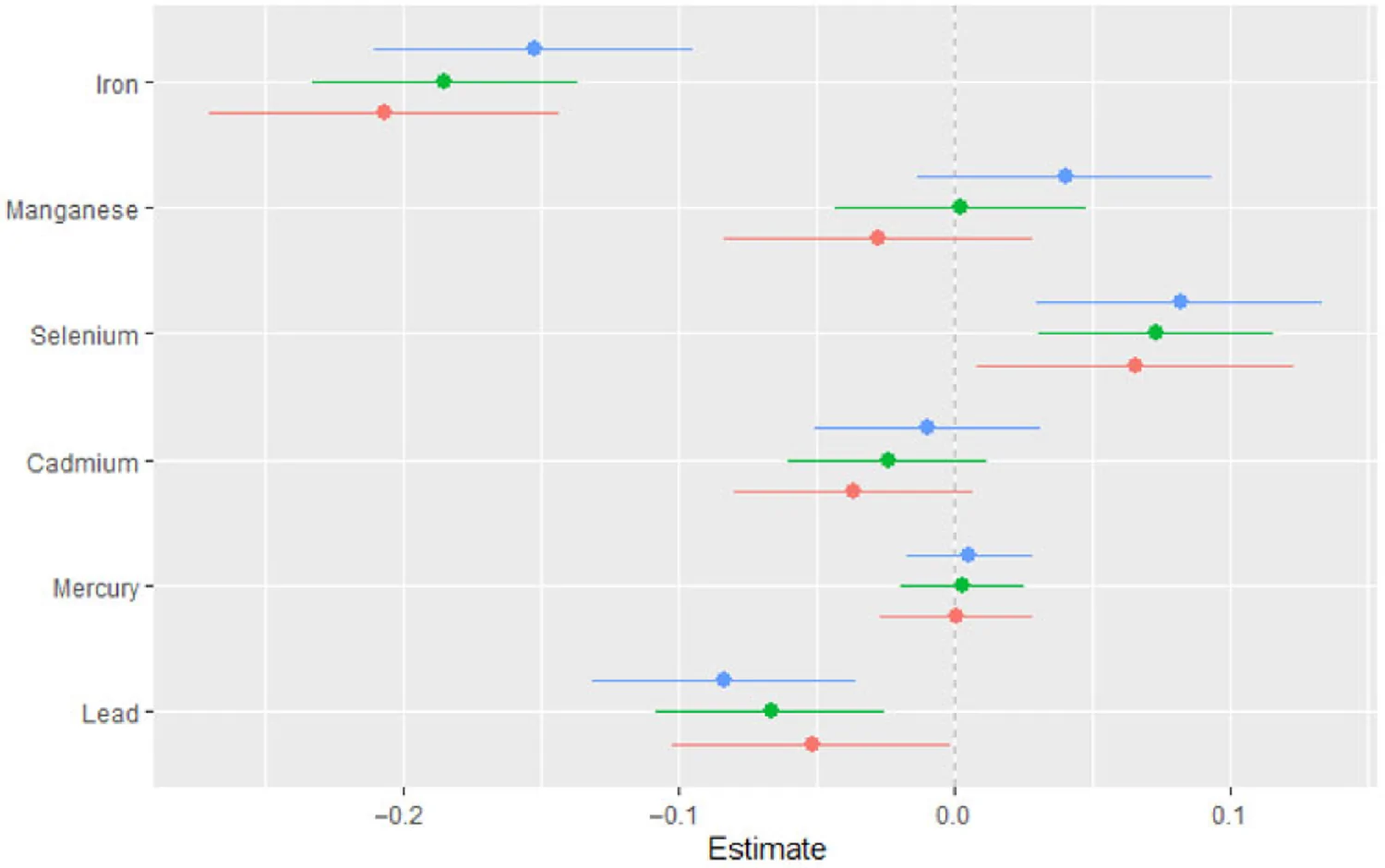
Estimated single-exposure effects with 95% credible intervals: the change in h associated with increasing each element from its 25th to its 75th percentile, with all other elements fixed at their 25th (red), 50th (green), and 75th (blue) percentiles. Adjusted for age, ethnicity, sex, BMI, smoking, alcohol use, income, education, and physical activity.

**Figure 13.**
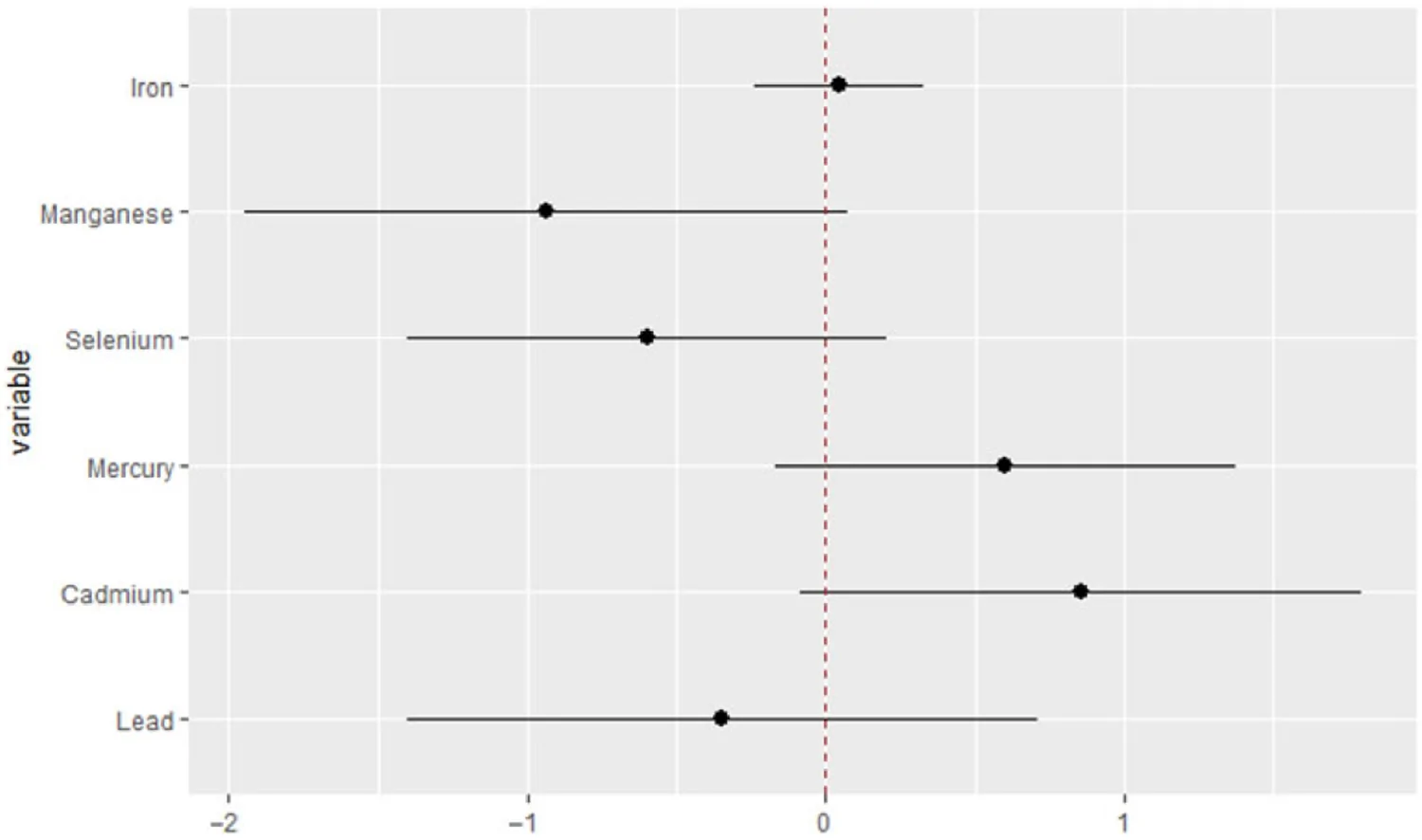
Estimated single-exposure effects with 95% credible intervals: the change in h associated with increasing each element from its 25th to its 75th percentile, with all other elements fixed at their medians. The dashed vertical line marks the null. Adjusted for age, ethnicity, sex, BMI, smoking, alcohol use, income, education, and physical activity.

Figure 12 shows the change in h associated with increasing each element from its 25th to its 75th percentile while the remaining elements are fixed at their 25th, 50th, or 75th percentiles. Iron showed a consistent inverse association whose credible interval excluded the null across all three reference levels, and lead showed a similar, smaller inverse association. Selenium showed a positive association in this reference specification. The remaining elements, manganese, cadmium, and mercury, had point estimates near the null with credible intervals spanning zero. When the same single-exposure effects were computed with all other elements fixed at their medians (Figure 13), credible intervals were wide and included the null for every element; point estimates were positive for cadmium and mercury and inverse for manganese, selenium, and lead, but none excluded zero. Because lead and iron are U-shaped with troughs near the center of the observed exposure distribution, a 25th-to-75th contrast traverses the descending limb of these curves, which accounts for their inverse single-exposure estimates even though the upper tail of each curve rises. Overall, iron was the only element with a robustly non-null independent association; the remaining single-exposure estimates should be read in light of their wide uncertainty, their sensitivity to how co-exposures are fixed, and the non-monotonic exposure–response shapes.

#### 3.9.5. Overall Mixture Effect

**Figure 14.**
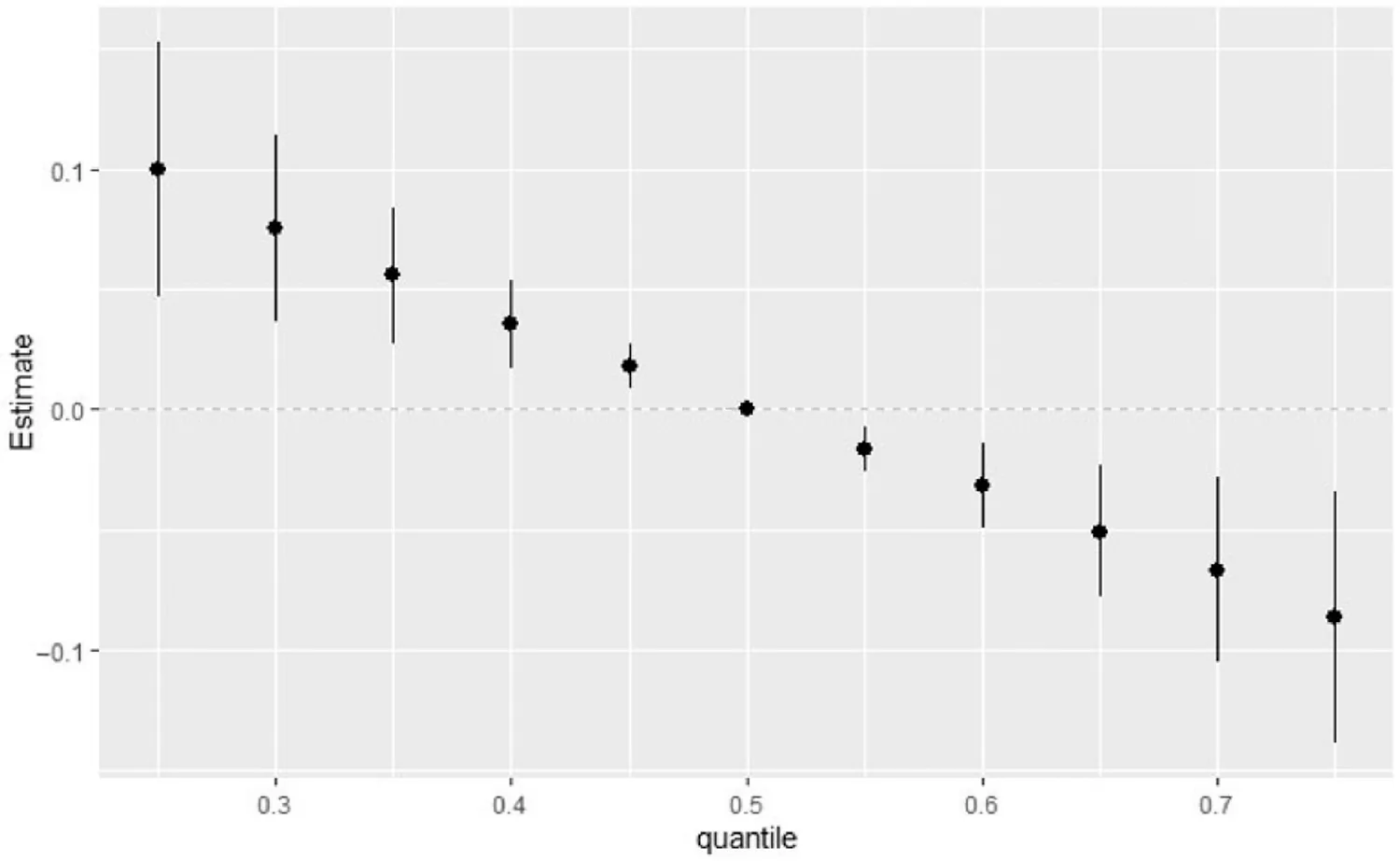
Overall estimated association of the combined metal mixture with prevalent diabetes, comparing h(z) with all elements at a given percentile against h(z) with all elements at the median. Adjusted for age, ethnicity, sex, BMI, smoking, alcohol use, income, education, and physical activity.

Setting all six elements jointly to a common percentile and comparing with the median (Figure 14), the overall mixture–response showed a monotonic inverse trend. At joint-exposure levels below the median, the mixture was associated with a small positive change in h on the latent probit scale, and at levels above the median the association became progressively negative, with credible intervals excluding the null at the lowest and highest quantiles. In other words, increasing the joint metal mixture from low to high over the observed range was associated with a steady decline in h and therefore in the modeled probability of prevalent diabetes, although the plotted quantity is the latent-scale contrast rather than a probability difference. This net inverse trend is consistent with the operative (descending) portions of the U-shaped lead and iron curves and the inverse manganese contribution outweighing the positive cadmium and mercury contributions when all elements increase together.

## 4. Discussion

Using a unified simulation framework, we benchmarked eight analytic approaches on their ability to predict outcomes and recover interaction structure across linear, nonlinear, continuous, and binary settings, and we illustrated BKMR on a six-metal mixture and diabetes in NHANES. Our results reinforce the central message of Hao et al. (2025): no single mixture method is uniformly best, and performance depends on the scientific target and the underlying exposure–response structure [12]. In our additive scenarios, the methods were nearly indistinguishable on MSE, correlation, and AUC, which is consistent with Hao et al.’s observation that simpler, general-purpose approaches can perform as well as or better than purpose-built mixture methods when the underlying relationship is not dominated by complex interactions. The oracle-referenced metrics qualify this in an important way. In the binary additive scenarios, the best method sat within 0.006 to 0.013 of the Bayes-optimal AUC, so the observed equivalence is a ceiling effect rather than evidence that the methods are interchangeable; in the continuous additive scenarios, by contrast, MSE against the true conditional mean spread six-fold across methods even though MSE against the observed outcome spread by less than 10%. A benchmark that reports only observed-outcome error at a modest signal-to-noise ratio will therefore systematically understate how differently these methods estimate the underlying surface [12].

When interactions are present, however, the choice becomes consequential: methods that model interactions explicitly or flexibly, whether through enumerated pairwise interactions (Lasso and hierNet), through recursive partitioning as in Random Forests or through a flexible kernel response surface as in BKMR, substantially outperformed additive-only approaches (glmnet_main, qgcomp and gWQS) on the continuous oracle-referenced prediction metrics. Differences in binary discrimination were substantially smaller, and in LogitI glmnet_int, hierNet and glmnet_main were indistinguishable. SuperLearner, whose library pairs additive learners with a Random Forest but contains no explicit interaction terms, sat consistently between the two groups and should not be classed as additive-only, most clearly on oracle-referenced metrics for continuous outcomes. Thus, our findings extend the broader method-selection guidance of Hao et al. by showing, at the level of specific simulated pairs, that an additive model can preserve reasonable performance when the truth is additive but can lose a substantial predictive signal when the health effect depends on interaction structure [12].

For explicit interaction detection, glmnet_int and hierNet perform comparably. Tuned by 10-fold cross-validation for both outcome types, they differ in sensitivity by no more than four percentage points, and the sign of the difference is not consistent across scenarios (hierNet higher in LMI and glmnet_int higher in NMI, LogitI and NLogitI). The difference that is consistent is in false discovery: hierNet incurs a lower mean per-replicate false discovery proportion in the paired comparison by roughly two to three percentage points in every interaction scenario and for both outcome types. The pooled false discovery rate, which we take as the more interpretable summary, points the same way for continuous outcomes (0.795 versus 0.810 in LMI, 0.789 versus 0.803 in NMI) but the other way for binary outcomes (0.816 versus 0.792 in LogitI, 0.811 versus 0.790 in NLogitI), because hierNet selects nothing far more often under a binary outcome and selects more pairs when it does select. We therefore describe the advantage as conditional on the reporting convention and the outcome type rather than as uniform. This pattern is consistent with the design of hierNet described by Bien et al. [15]. hierNet enforces hierarchical sparsity, meaning that an interaction can be selected only when its parent main effects are also included. Although this constraint can produce a smaller and more interpretable set of candidate interactions, it also imposes a substantive assumption about the underlying data-generating process. By including a true interaction in which one of the two exposures had no independent main effect, our simulation tested a setting in which that heredity assumption did not hold. Aggregate sensitivity did not differ appreciably between the two methods, so our results do not establish that the heredity-violating pair drove a difference in recovery; what they show is that the structural restriction did not translate into a material overall penalty in this design. The practical implication follows directly. Because the two enumerating methods perform comparably, the choice between them should rest on whether the strong-heredity assumption is scientifically plausible in the application at hand, not on an expected sensitivity advantage. Strong heredity can structurally disadvantage recovery of an interaction whose parent carries no main effect, because the parent must enter the model first, and glmnet_int is not subject to that restriction. Our aggregate sensitivity results do not isolate the contribution of the single heredity-violating pair and should not be read as demonstrating that mechanism empirically; the point is structural, and pair-specific recovery probabilities would be needed to quantify it. Where heredity is scientifically plausible, hierNet’s structural constraint may aid interpretability; its mean per-replicate false discovery proportion was modestly lower throughout, while the pooled false discovery rate and the size of the selected set depended on outcome type, with hierNet selecting fewer pairs than glmnet_int for continuous outcomes and more for binary ones. All contrasts reported here are paired across replicates with Monte Carlo intervals, so they are significance-tested rather than descriptive; the caveat that now matters is the opposite one, namely, that with 500 replicates the study resolves differences much smaller than those that would change practice, and the intervals should be read before the ordering. The dominant feature of the detection results is not the difference between the methods, but what they share: a pooled false discovery rate near 0.80 in every interaction scenario. With three true pairs among 45 candidates, roughly four in five selected pairs are false. This was observed for both methods in the continuous interaction scenarios and for glmnet_int in the binary scenarios, and it persisted under the pure-product sensitivity analysis. Selected interactions require replication before they are treated as findings.

BKMR results obtained with a short chain characterize a sampling budget, not a method. At 2000 iterations, the chains were unambiguously non-convergent, with 93% of monitored parameters in the continuous scenario and 76% in the binary scenario above a Gelman–Rubin statistic of 1.1, and minimum effective sample sizes of 7.5 and 19.8 respectively. At 25,000 iterations the diagnostics were substantially improved and broadly acceptable, although 5.0% and 1.8% of monitored quantities remained above 1.1, with maxima of 1.25 and 1.22, so we describe that budget as adequate for this design rather than as a general recommendation. Every prediction metric improved significantly at the longer budget, and the replicate-to-replicate variability that would otherwise read as instability fell by roughly an order of magnitude. The sample-size analysis makes the mechanism plain: at a fixed budget, BKMR’s performance degrades as *n* grows, which is the signature of an inadequate budget rather than of a method that scales badly. The runtime comparison requires the same qualification. For an MCMC method, wall-clock time is a linear function of an analyst-chosen iteration count, so a statement that BKMR is several orders of magnitude slower than a penalized method is a statement about the budget the analyst selected. Reported per 1000 iterations within the paired budget experiment, BKMR’s cost was 118 to 123 s at the short budget and 251 to 281 s at the long one for the continuous scenarios, and 133 to 188 and 499 to 614 respectively for the binary scenarios, the increase reflecting the explicit sampling of the exposure–response surface, and the meaningful quantity is that cost multiplied by the roughly 25,000 iterations this design requires for acceptable diagnostics. That is a real and substantial computational burden, and it is the practical cost of obtaining BKMR’s flexibility and posterior uncertainty quantification [16,17]. Our BKMR findings should also be interpreted considering what the method was originally designed to do. Bobb et al. introduced BKMR as a flexible kernel-based framework for estimating a multivariable exposure–response surface, accommodating nonlinearities and interactions, and identifying influential mixture components through hierarchical variable selection [16]. The later bkmr software paper (version 0.2.2, the version used in the present study) emphasized interpretable summaries such as posterior inclusion probabilities, univariate and bivariate exposure–response functions, single-exposure contrasts, and overall mixture effects, including an extension for binary outcomes [17]. These strengths were evident in our applied analysis, where BKMR produced a coherent set of descriptive outputs that could not be obtained directly from the explicit interaction-selection models. Our results therefore support the use of BKMR when the goal is to characterize the shape and joint behavior of a mixture, but they also show why adequate chain length, convergence assessment, and sensitivity analyses are essential before using it as a predictive benchmark.

Two further points follow. First, the ranking analysis shows that BKMR’s interaction detection is understated by its usual exclusion from detection comparisons: scored on the ordering of all 45 candidate pairs, it was competitive with both penalized methods in every interaction scenario and slightly ahead of them in three of four. The bivariate summaries that applied users already inspect informally do carry recoverable information about which pairs interact. Second, fitting BKMR to binary outcomes required attention to the sampler. Under the default kmbayes configuration with componentwise variable selection, every binomial fit at *n* = 500 terminated with a non-positive-definite augmented kernel matrix; setting est.h = TRUE, which samples the exposure–response surface explicitly rather than marginalizing it out, resolved the failure completely and is the configuration used throughout. This is a practical trap rather than a limitation of the method, but it is one that applied users fitting binomial BKMR to mixtures of this dimension are likely to encounter, and it is reported here because the diagnostic path to it is not obvious from the package documentation.

In the applied analysis, lead, manganese, and iron emerged as the most influential mixture components by posterior inclusion probability, the exposure–response functions were predominantly nonlinear, and the overall mixture showed a monotonic inverse association with prevalent diabetes over the observed range. The bivariate and quantile-stratified surfaces gave little indication of strong pairwise interaction among the elements. These applied results are intended to illustrate the descriptive outputs that BKMR provides rather than to support causal claims; the nonlinear and non-monotonic shapes, particularly for lead and iron, underscore why single-number summaries can differ in sign from the full exposure–response curve and should be interpreted jointly. The inverse overall direction, higher joint metal exposure associated with lower modeled diabetes probability, runs counter to the positive metal–diabetes associations commonly reported and most plausibly reflects the cross-sectional, unweighted design rather than a protective effect: reverse causation (e.g., altered iron handling and dietary change in established diabetes) and residual confounding, with the inverse iron-biomarker contribution driving much of the trend, are the likely explanations. These findings also illustrate the types of summaries for which BKMR was developed. Posterior inclusion probabilities describe the relative importance of each component conditional on the other elements in the mixture, while the univariate, bivariate, and overall-mixture functions reveal features that would be obscured by a single linear coefficient [16,17].

Several limitations should be acknowledged. First, although the primary benchmark fixes the exposure correlation, the signal level, the sample size, the prevalence, the number of exposures, and the number of true interactions, we varied the first four of these plus the form of the interaction term in the sensitivity analyses of Sections 2.4 and 3.6, with all eight methods evaluated at every level, and the broad qualitative pattern was stable across these variations, although exact rankings among individual methods shifted in some settings. The number of exposures, the prevalence, and the number of true interactions remain fixed, and the rankings may not generalize to designs that differ in those respects. Second, all methods were run with default or recommended settings to reflect typical practice, which disadvantages methods that benefit from tuning. For BKMR, we addressed this directly by treating the sampling budget as an experimental factor, but the point applies more broadly, and no method here was tuned to its ceiling. Third, one of the three simulated interactions involved an exposure (X_6_) without a corresponding main effect, which structurally disadvantages strong-heredity methods such as hierNet, since the parent must also enter the model. This heredity-violating pair was included intentionally, alongside two heredity-satisfying pairs, so that methods were evaluated under both interaction structures rather than under strong heredity alone; it is a deliberate feature of the benchmark rather than an incidental misspecification. It did not translate into materially lower overall detection for hierNet, but our aggregate sensitivity results do not isolate the contribution of that single pair, and a per-pair decomposition of recovery would be needed to quantify it. Fourth, because outcome variance differs across data-generating processes, MSE magnitudes are comparable only within scenarios. Fifth, the Random Forest interaction statistic could not be computed for binary outcomes with the implementation used, so the ranking comparison covers four methods for continuous outcomes and three for binary ones. Sixth, the applied analysis is intended solely as a methodological illustration of BKMR’s descriptive outputs and not as a nationally representative inference: it is cross-sectional, unweighted, and does not incorporate the NHANES complex survey design.

In summary, our findings agree with prior comparison work that method selection in environmental mixtures should be driven by the scientific question and the complexity of the underlying exposure–response relationship. When exposures act additively, method choice has little consequence for prediction measured against the observed outcome, although estimation of the underlying conditional mean still differs substantially across methods; when interactions are present, interaction-aware methods become important and additive-only methods can be misleading, with the advantage most evident in nonlinear interaction scenarios and on oracle-referenced continuous metrics. SuperLearner, whose library includes a flexible learner but no explicit interaction terms, occupied a consistent intermediate position and should not be grouped with the additive-only methods. On binary discrimination, the additive penalty was present but modest, and in LogitI, glmnet_int, hierNet and glmnet_main were indistinguishable. The comparison between glmnet_int and hierNet shows that the two perform comparably once tuned identically by 10-fold cross-validation and scored against the design’s true pair set, with hierNet slightly ahead on sensitivity for continuous outcomes and glmnet_int slightly ahead for binary ones, and hierNet holding a modestly lower mean per-replicate false discovery proportion in paired comparisons throughout, although pooled false discovery favors hierNet only in the continuous scenarios and favors glmnet_int in the binary ones; the choice between them therefore turns on whether the strong-heredity assumption is scientifically plausible rather than on an expected difference in detection rate. Both leave roughly four in five selected pairs false at this signal level. BKMR remains especially valuable for its flexible, uncertainty-aware characterization of nonlinear and joint exposure–response relationships, and its apparent weaknesses in an under-budgeted benchmark are artifacts of that budget; the requirement this imposes is that any comparison involving BKMR must state its iteration count and report convergence diagnostics, without which the comparison is uninterpretable.

## 5. Conclusions

Method selection for environmental mixture analysis should be guided by the scientific objective and expected exposure–response structure. Additive methods performed similarly when effects were additive, but interaction-capable methods performed better when interactions were present, a contrast that oracle-referenced metrics show to be considerably larger than observed-outcome error suggests. The interaction Lasso and hierNet performed comparably on detection once they were tuned identically and scored against the specified pair set, with the relative false discovery advantage depending on the aggregation convention and the outcome type: hierNet had the lower mean per-replicate false discovery proportion throughout, while the pooled false discovery rate favored hierNet for continuous outcomes and glmnet_int for binary ones; both left roughly four in five selected pairs false, so selected interactions require replication rather than direct interpretation. BKMR was useful for describing nonlinear and joint mixture effects and, scored on the ranking of candidate pairs, was competitive with the penalized methods on pair-ranking ROC AUC; its poorer prediction and stability with a short chain reflected an inadequate sampling budget rather than the method, and comparisons involving it are interpretable only when the iteration count and convergence diagnostics are reported. These findings provide practical guidance for selecting mixture methods for prediction, interaction detection, and exposure–response characterization.

## Supplementary Material

Supplementary

**Supplementary Materials:** The following supporting information can be downloaded at https://www.mdpi.com/article/10.3390/stats9050096/s1: Table S1: Variance decomposition of the systematic component in the interaction scenarios; Table S2: Signal strength of the binary data-generating processes; Table S2b: Signal strength across the three latent-scale signal levels; Table S3: hierNet detection tolerance sensitivity; Table S4: Prediction performance for continuous outcomes, mean, SD and MCSE; Table S5: Classification performance for binary outcomes and runtime, mean, SD and MCSE; Table S5b: Runtime for continuous-outcome scenarios; Table S6: Interaction detection, mean, SD and MCSE; Table S6b: Distribution of the number of interaction pairs selected per replicate; Table S6c: Verification of the false discovery rate computation; Table S7: Ranking-based interaction detection; Table S8: Paired between-method differences in prediction, continuous outcomes; Table S9: Paired between-method differences in classification, binary outcomes; Table S10: Paired between-method differences in interaction detection; Table S11: BKMR sampling budget, performance at 2000 and 25,000 MCMC iterations; Table S12: Paired within-replicate effect of the BKMR sampling budget; Table S13: BKMR multi-chain convergence diagnostics; Table S13b: BKMR sampler configuration for the binomial family; Table S14: Sensitivity to sample size; Table S15: Sensitivity to the exposure correlation structure; Table S16: Sensitivity to signal strength; Table S16b: Classification performance across binary signal-strength levels; Table S17: Pure-product interaction variant, detection; Table S17b: Prediction under the pure-product variant; Table S18: Computational cost of the study, measured from the completed runs. BKMR multi-chain trace plots for every dataset and budget are provided as separate image files.

## Figures and Tables

**Table 1.** Posterior inclusion probabilities (PIPs) for the toxic metals and essential elements in relation to prevalent diabetes.

Exposure	PIP
Lead	1.0000
Mercury	0.2596
Cadmium	0.6448
Selenium	0.8764
Manganese	1.0000
Iron	1.0000

## Data Availability

The NHANES dataset is publicly available online, accessible at https://wwwn.cdc.gov/nchs/nhanes/continuousnhanes/overview.aspx?BeginYear=2017 (accessed on 22 June 2026). The R code used for the simulation study is available on GitLab at https://gitlab.com/agadetunji/simulation (accessed on 1 September 2026).
