# Supplementary material for: Benchmarking Statistical Methods for Environmental Chemical Mixtures: Prediction, Interaction Detection, and an Applied Analysis of Metals, Essential Elements and Diabetes
Source: Stats (Basel). Author manuscript; Available in PMC 2026 Sep 18. (PMC13580283; doi:10.3390/stats9050096)
Supplement: Supplementary file 1 [file NIHMS2209559-supplement-Supplementary.pdf]

Supplementary Materials

Benchmarking Statistical Methods for Environmental Chemical Mixtures: Prediction, Interaction Detection, and an Applied Analysis of Metals, Essential Elements and Diabetes

All simulation results are based on 500 replicates per scenario unless stated otherwise. MCSE denotes the Monte Carlo standard error of the mean across replicates. Interaction detection is scored against the three true pairs (X1,X2), (X3,X4) and (X5,X6) specified in Section 2.1.2, giving 3 true and 42 null candidate pairs in the interaction scenarios and 0 true and 45 null candidate pairs in the scenarios without interactions.

Table S1. Variance decomposition of the systematic component in the interaction scenarios

Scenario	Var(total)	Var(main)	Var(product)	Var(quadratic threshold)	Covariance residual	Prop. main	Prop. product	Prop. quadratic	Prop. covariance
LMI	13.1003	8.4243	2.0572	0.2427	2.3761	0.6431	0.1570	0.0185	0.1814
NMI	11.9341	5.6629	2.0572	0.2427	3.9714	0.4745	0.1724	0.0203	0.3328

Monte Carlo decomposition over 50,000 draws. The systematic component is the sum of a main-effect term, a pure product term 0.6·Xk·Xl summed over the three true pairs, and a quadratic threshold term 0.2·Xk²·I(Xl>0) summed over the same pairs; the residual is the covariance among these components, which is non-zero because the exposures are correlated. The quadratic threshold term contributes under 2.1% of systematic variance in both scenarios, so the interaction signal is dominated by the product term. True pairs: 1:2,3:4,5:6. 1:2 and 3:4 satisfy strong heredity; 5:6 violates it (X6 has no main effect).

Table S2. Signal strength of the binary data-generating processes

Scenario	Replicates	Tjur's D (mean)	SD	MCSE	McFadden R² (mean)	SD	MCSE	Prevalence
Logit	500	0.2354	0.0187	0.0008	0.1872	0.0297	0.0013	0.5009
LogitI	500	0.1972	0.0174	0.0008	0.1571	0.0254	0.0011	0.4760
Nlogit	500	0.2298	0.0188	0.0008	0.1813	0.0293	0.0013	0.4838
NlogitI	500	0.1838	0.0174	0.0008	0.1468	0.0244	0.0011	0.4717

Computed from the true generating probabilities on the held-out test set, so these are oracle values and represent the maximum attainable discrimination. Tjur's coefficient of discrimination is the difference in mean fitted probability between cases and non-cases; McFadden's pseudo-R² is 1 − logL(model)/logL(null). These quantities are reported in response to the request for an interpretable measure of binary signal strength, which the latent-scale scaling factor k does not provide.

Table S2b. Signal strength across the three latent-scale signal levels

Scenario	Target SD of η	Replicates	Tjur's D	MCSE	McFadden R²	MCSE	Prevalence
LogitI	0.75	50	0.0949	0.0017	0.0712	0.0026	0.4930
LogitI	1.25	50	0.1983	0.0026	0.1583	0.0039	0.4754
LogitI	1.75	50	0.2943	0.0029	0.2416	0.0042	0.4576
NlogitI	0.75	50	0.0912	0.0016	0.0701	0.0025	0.4906
NlogitI	1.25	50	0.1846	0.0027	0.1477	0.0039	0.4740
NlogitI	1.75	50	0.2808	0.0028	0.2317	0.0036	0.4537

The latent-scale scaling factor k is set so that the linear predictor has the target standard deviation shown. Because k is not itself interpretable as an effect size, the corresponding oracle Tjur and McFadden values are given to place the three signal levels on an interpretable scale.

Table S3. hierNet detection tolerance sensitivity

Scenario	Tolerance	Replicates	Sensitivity	FDR (mean FDP)	FPR	Mean pairs selected
LMI	0	50	0.6533	0.7392	0.1514	8.32
LMI	1e-12	50	0.6533	0.7392	0.1514	8.32
LMI	1e-10	50	0.6533	0.7392	0.1514	8.32
LMI	1e-8	50	0.6533	0.7385	0.1510	8.30
LMI	1e-6	50	0.6533	0.7361	0.1481	8.18
NMI	0	50	0.6733	0.7699	0.1895	9.98
NMI	1e-12	50	0.6733	0.7699	0.1895	9.98
NMI	1e-10	50	0.6733	0.7699	0.1895	9.98
NMI	1e-8	50	0.6733	0.7699	0.1895	9.98
NMI	1e-6	50	0.6667	0.7688	0.1871	9.86
LogitI	0	50	0.5400	0.7495	0.1686	8.70
LogitI	1e-12	50	0.5400	0.7495	0.1686	8.70
LogitI	1e-10	50	0.5400	0.7388	0.1681	8.68
LogitI	1e-8	50	0.5400	0.7388	0.1681	8.68
LogitI	1e-6	50	0.5400	0.7380	0.1671	8.64
NlogitI	0	50	0.6733	0.7332	0.1857	9.82
NlogitI	1e-12	50	0.6733	0.7332	0.1857	9.82
NlogitI	1e-10	50	0.6733	0.7332	0.1857	9.82
NlogitI	1e-8	50	0.6733	0.7332	0.1857	9.82
NlogitI	1e-6	50	0.6667	0.7348	0.1852	9.78

The 1e-8 cut-off used in the main analysis is a floating-point tolerance for numerical zero, not a statistical selection threshold: the L1 penalty in hierNet sets interaction parameters to exact zero, and the statistical tuning parameter is lambda, chosen by 10-fold cross-validation. Across exact zero and nonzero tolerances from 1e-12 to 1e-6 the mean number of selected pairs moves by at most 0.14 and sensitivity is unchanged to three decimal places in three of four scenarios, so no conclusion depends on the tolerance. The false discovery column is the mean of the per-replicate false discovery proportion and therefore conditions on replicates that selected at least one pair; it is not the pooled false discovery rate reported in Table S6, and the two differ by 0.02 to 0.08 as documented in Table S6c. Based on 50 replicates per scenario.

Table S4. Prediction performance for continuous outcomes: mean, SD and MCSE

Scenario	Method	Fits	MSE	SD	MCSE	Oracle MSE	SD	MCSE	r (observed)	r (oracle)	MCSE
LM	glmnet_main	500	34.063	2.614	0.1169	0.559	0.294	0.0131	0.4354	0.9762	0.0007
LM	glmnet_int	500	34.270	2.635	0.1179	0.772	0.404	0.0181	0.4315	0.9675	0.0010
LM	hierNet	500	34.214	2.645	0.1183	0.711	0.398	0.0178	0.4319	0.9684	0.0010
LM	rf	500	35.368	2.687	0.1202	1.863	0.341	0.0153	0.4011	0.8993	0.0010
LM	BKMR	500	36.845	3.978	0.1779	3.372	2.939	0.1314	0.3506	0.7849	0.0091
LM	qgcomp	500	34.813	2.655	0.1187	1.347	0.350	0.0156	0.4147	0.9279	0.0009
LM	gWQS	500	34.565	2.625	0.1174	1.082	0.279	0.0125	0.4212	0.9433	0.0006
LM	SuperLearner	500	34.162	2.629	0.1176	0.668	0.327	0.0146	0.4326	0.9692	0.0008
LMI	glmnet_main	500	55.274	6.085	0.2721	3.797	0.745	0.0333	0.3814	0.8529	0.0010
LMI	glmnet_int	500	53.327	5.894	0.2636	1.872	0.710	0.0318	0.4192	0.9368	0.0011
LMI	hierNet	500	53.021	5.857	0.2619	1.568	0.711	0.0318	0.4245	0.9485	0.0010
LMI	rf	500	54.906	6.106	0.2731	3.419	0.701	0.0313	0.3898	0.8723	0.0011
LMI	BKMR	500	57.941	8.163	0.3651	6.422	4.933	0.2206	0.3280	0.7357	0.0095
LMI	qgcomp	500	56.383	6.355	0.2842	4.937	1.017	0.0455	0.3590	0.8022	0.0014
LMI	gWQS	500	55.896	6.289	0.2812	4.429	0.874	0.0391	0.3682	0.8235	0.0010
LMI	SuperLearner	500	54.280	6.027	0.2695	2.812	0.691	0.0309	0.4000	0.8944	0.0010
NM	glmnet_main	500	23.416	1.974	0.0883	1.112	0.238	0.0107	0.4031	0.9065	0.0009
NM	glmnet_int	500	23.207	1.950	0.0872	0.913	0.292	0.0131	0.4131	0.9285	0.0011
NM	hierNet	500	23.075	1.951	0.0872	0.780	0.263	0.0118	0.4178	0.9389	0.0010

NM	rf	500	23.650	2.000	0.0894	1.348	0.252	0.0113	0.3949	0.8878	0.0010
NM	BKMR	500	24.670	2.764	0.1236	2.359	2.012	0.0900	0.3466	0.7814	0.0088
NM	qgcomp	500	23.691	2.039	0.0912	1.370	0.281	0.0125	0.3918	0.8823	0.0010
NM	gWQS	500	23.503	2.011	0.0899	1.174	0.236	0.0105	0.3991	0.8999	0.0008
NM	SuperLearner	500	23.269	1.965	0.0879	0.964	0.244	0.0109	0.4088	0.9195	0.0009
NMI	glmnet_main	500	52.855	6.044	0.2703	6.268	0.962	0.0430	0.3075	0.6938	0.0019
NMI	glmnet_int	500	48.549	5.602	0.2505	1.953	0.640	0.0286	0.4109	0.9235	0.0011
NMI	hierNet	500	48.491	5.589	0.2500	1.905	0.657	0.0294	0.4116	0.9254	0.0011
NMI	rf	500	49.960	5.774	0.2582	3.407	0.648	0.0290	0.3806	0.8544	0.0012
NMI	BKMR	500	51.657	7.411	0.3314	5.171	4.748	0.2123	0.3453	0.7746	0.0094
NMI	qgcomp	500	53.530	6.255	0.2797	6.917	1.102	0.0493	0.2914	0.6583	0.0017
NMI	gWQS	500	53.028	6.165	0.2757	6.411	0.998	0.0446	0.3031	0.6852	0.0014
NMI	SuperLearner	500	49.876	5.752	0.2572	3.311	0.731	0.0327	0.3808	0.8557	0.0013

MSE is computed against the observed test outcome and oracle MSE against the true conditional mean, which removes irreducible noise and separates the methods far more sharply. Correlations are Pearson correlations of predictions with the observed outcome and with the true conditional mean respectively. All eight methods completed in all 500 replicates of all four scenarios.

Table S5. Classification performance for binary outcomes and runtime: mean, SD and MCSE

Scenario	Method	Fits	AUC	SD	MCSE	Oracle AUC	Deficit vs oracle	Runtime (s)	SD
Logit	glmnet_main	500	0.7744	0.0221	0.0010	0.7801	0.0057	0.43	0.24
Logit	glmnet_int	500	0.7730	0.0225	0.0010	0.7801	0.0071	0.49	0.17
Logit	hierNet	500	0.7711	0.0232	0.0010	0.7801	0.0090	236.21	65.25
Logit	rf	500	0.7538	0.0229	0.0010	0.7801	0.0264	0.96	0.36
Logit	BKMR	500	0.7654	0.0286	0.0013	0.7801	0.0148	686.43	216.98
Logit	qgcomp	500	0.7669	0.0227	0.0010	0.7801	0.0132	0.04	0.06
Logit	gWQS	500	0.7707	0.0222	0.0010	0.7801	0.0095	27.70	15.93
Logit	SuperLearner	500	0.7731	0.0222	0.0010	0.7801	0.0070	4.59	1.60
LogitI	glmnet_main	500	0.7316	0.0223	0.0010	0.7470	0.0154	0.40	0.14
LogitI	glmnet_int	500	0.7319	0.0231	0.0010	0.7470	0.0151	0.81	0.40
LogitI	hierNet	500	0.7319	0.0233	0.0010	0.7470	0.0151	238.60	18.26
LogitI	rf	500	0.7154	0.0236	0.0011	0.7470	0.0316	0.63	0.30
LogitI	BKMR	500	0.7199	0.0332	0.0015	0.7470	0.0271	647.49	118.91
LogitI	qgcomp	500	0.7248	0.0228	0.0010	0.7470	0.0222	0.03	0.03
LogitI	gWQS	500	0.7298	0.0219	0.0010	0.7470	0.0172	24.04	5.17
LogitI	SuperLearner	500	0.7299	0.0224	0.0010	0.7470	0.0171	4.54	1.09
Nlogit	glmnet_main	500	0.7609	0.0222	0.0010	0.7737	0.0129	0.39	0.26
Nlogit	glmnet_int	500	0.7589	0.0223	0.0010	0.7737	0.0148	0.54	0.18
Nlogit	hierNet	500	0.7588	0.0231	0.0010	0.7737	0.0149	235.74	18.23
Nlogit	rf	500	0.7462	0.0224	0.0010	0.7737	0.0276	0.38	0.08
Nlogit	BKMR	500	0.7571	0.0256	0.0011	0.7737	0.0166	684.86	111.45
Nlogit	qgcomp	500	0.7558	0.0224	0.0010	0.7737	0.0179	0.03	0.03
Nlogit	gWQS	500	0.7600	0.0221	0.0010	0.7737	0.0137	25.30	5.47
Nlogit	SuperLearner	500	0.7601	0.0222	0.0010	0.7737	0.0137	4.38	1.02
NlogitI	glmnet_main	500	0.6835	0.0266	0.0012	0.7338	0.0503	0.38	0.16
NlogitI	glmnet_int	500	0.7074	0.0253	0.0011	0.7338	0.0264	0.57	0.18
NlogitI	hierNet	500	0.7040	0.0260	0.0012	0.7338	0.0298	258.48	21.28
NlogitI	rf	500	0.6982	0.0251	0.0011	0.7338	0.0356	0.39	0.09
NlogitI	BKMR	500	0.6987	0.0321	0.0014	0.7338	0.0350	735.76	141.36
NlogitI	qgcomp	500	0.6791	0.0267	0.0012	0.7338	0.0547	0.03	0.04
NlogitI	gWQS	500	0.6859	0.0257	0.0012	0.7338	0.0479	25.01	5.61
NlogitI	SuperLearner	500	0.6995	0.0250	0.0011	0.7338	0.0343	4.50	1.09

The oracle AUC is the Bayes-optimal value obtainable from the true generating probabilities and is identical for all methods within a scenario. The deficit column is the gap between the oracle and each method. BKMR runtimes are for the 2,000-iteration primary budget under the est.h = TRUE sampler configuration described in Section 2.2.4.

Table S5b. Runtime for continuous-outcome scenarios

Scenario	Method	Runtime (s)	SD	MCSE	Seconds per 1,000 iterations
LM	glmnet_main	0.862	0.685	0.0307	
LM	glmnet_int	0.558	0.473	0.0212	
LM	hierNet	63.512	13.343	0.5967	
LM	rf	1.609	1.097	0.0491	
LM	BKMR	639.199	138.483	6.1932	319.6
LM	qgcomp	0.036	0.032	0.0014	
LM	gWQS	29.152	8.226	0.3679	
LM	SuperLearner	5.263	1.976	0.0884	
LMI	glmnet_main	0.481	0.554	0.0248	
LMI	glmnet_int	0.292	0.174	0.0078	
LMI	hierNet	68.602	8.267	0.3697	
LMI	rf	1.685	0.590	0.0264	
LMI	BKMR	500.139	97.972	4.3815	250.1
LMI	qgcomp	0.036	0.042	0.0019	
LMI	gWQS	27.734	6.111	0.2733	
LMI	SuperLearner	4.438	1.260	0.0564	
NM	glmnet_main	0.361	0.126	0.0057	
NM	glmnet_int	0.256	0.106	0.0047	
NM	hierNet	62.709	6.772	0.3029	
NM	rf	1.636	0.478	0.0214	
NM	BKMR	508.238	110.222	4.9293	254.1
NM	qgcomp	0.035	0.034	0.0015	
NM	gWQS	27.914	5.998	0.2682	
NM	SuperLearner	4.511	1.636	0.0732	
NMI	glmnet_main	0.580	0.390	0.0174	
NMI	glmnet_int	0.363	0.228	0.0102	
NMI	hierNet	75.493	12.643	0.5654	
NMI	rf	1.793	0.666	0.0298	
NMI	BKMR	563.090	111.749	4.9976	281.5
NMI	qgcomp	0.041	0.057	0.0026	
NMI	gWQS	29.264	7.556	0.3379	
NMI	SuperLearner	5.230	2.164	0.0968	

Wall-clock seconds per replicate, including preprocessing, tuning, fitting and prediction, on 64 cores of a shared HPC node with one thread per worker. Seconds per 1,000 iterations is defined only for BKMR, the sole iterative method, and is the appropriate basis for comparison because total runtime for an MCMC method is a linear function of an analyst-chosen budget rather than a property of the algorithm.

Table S6, part 1. Interaction detection, recovery of true pairs: mean, SD and MCSE

Scenario	Outcome	Method	Fits	True pairs	Sensitivity	SD	MCSE	Specificity	SD	MCSE
LM	continuous	hierNet	500	0				0.8953	0.0947	0.0042
LM	continuous	glmnet_int	500	0				0.9201	0.0808	0.0036

LMI	continuous	hierNet	500	3	0.6060	0.2729	0.0122	0.8326	0.0897	0.0040
LMI	continuous	glmnet_int	500	3	0.5887	0.2892	0.0129	0.8210	0.0954	0.0043
NM	continuous	hierNet	500	0				0.8502	0.0858	0.0038
NM	continuous	glmnet_int	500	0				0.8537	0.0929	0.0042
NMI	continuous	hierNet	500	3	0.6513	0.2550	0.0114	0.8265	0.0894	0.0040
NMI	continuous	glmnet_int	500	3	0.6780	0.2569	0.0115	0.8023	0.0941	0.0042
Logit	binary	hierNet	500	0				0.8872	0.1598	0.0071
Logit	binary	glmnet_int	500	0				0.9213	0.0760	0.0034
LogitI	binary	hierNet	500	3	0.5320	0.3250	0.0145	0.8320	0.1538	0.0069
LogitI	binary	glmnet_int	500	3	0.5733	0.2787	0.0125	0.8442	0.0870	0.0039
Nlogit	binary	hierNet	500	0				0.8574	0.1481	0.0066
Nlogit	binary	glmnet_int	500	0				0.8596	0.0980	0.0044
NlogitI	binary	hierNet	500	3	0.6833	0.2783	0.0124	0.7903	0.1685	0.0075
NlogitI	binary	glmnet_int	500	3	0.7153	0.2603	0.0116	0.8079	0.0856	0.0038

Sensitivity is undefined in the scenarios without true interactions and is left blank. Specificity is the proportion of null candidate pairs correctly left unselected. Every quantity is reported as the mean across replicates, the replicate-to-replicate standard deviation, and the Monte Carlo standard error of the mean.

Table S6, part 2. Interaction detection, false discoveries and selected-set size: mean, SD and MCSE

Scenario	Outcome	Method	FDR (mean FDP)	SD	MCSE	FDR (pooled)	FPR	SD	MCSE	Pairs selected	SD	Prop. selecting none
LM	continuous	hierNet	1.0000	0.0000	0.0000	1.0000	0.1047	0.0947	0.0042	4.71	4.26	0.088
LM	continuous	glmnet_int	1.0000	0.0000	0.0000	1.0000	0.0799	0.0808	0.0036	3.59	3.64	0.172
LMI	continuous	hierNet	0.7670	0.1361	0.0061	0.7946	0.1674	0.0897	0.0040	8.85	3.85	0.000
LMI	continuous	glmnet_int	0.7860	0.1360	0.0061	0.8098	0.1790	0.0954	0.0043	9.28	4.20	0.000
NM	continuous	hierNet	1.0000	0.0000	0.0000	1.0000	0.1498	0.0858	0.0038	6.74	3.86	0.002
NM	continuous	glmnet_int	1.0000	0.0000	0.0000	1.0000	0.1463	0.0929	0.0042	6.58	4.18	0.010
NMI	continuous	hierNet	0.7628	0.1181	0.0053	0.7886	0.1735	0.0894	0.0040	9.24	3.89	0.000
NMI	continuous	glmnet_int	0.7835	0.1020	0.0046	0.8032	0.1977	0.0941	0.0042	10.34	4.11	0.000
Logit	binary	hierNet	1.0000	0.0000	0.0000	1.0000	0.1128	0.1598	0.0071	5.08	7.19	0.452
Logit	binary	glmnet_int	1.0000	0.0000	0.0000	1.0000	0.0787	0.0760	0.0034	3.54	3.42	0.180
LogitI	binary	hierNet	0.7366	0.2171	0.0102	0.8155	0.1680	0.1538	0.0069	8.65	6.99	0.086
LogitI	binary	glmnet_int	0.7636	0.1441	0.0065	0.7918	0.1558	0.0870	0.0039	8.26	3.90	0.004
Nlogit	binary	hierNet	1.0000	0.0000	0.0000	1.0000	0.1426	0.1481	0.0066	6.42	6.67	0.216
Nlogit	binary	glmnet_int	1.0000	0.0000	0.0000	1.0000	0.1404	0.0980	0.0044	6.32	4.41	0.050
NlogitI	binary	hierNet	0.7417	0.1731	0.0078	0.8112	0.2097	0.1685	0.0075	10.86	7.46	0.008
NlogitI	binary	glmnet_int	0.7681	0.1180	0.0053	0.7899	0.1921	0.0856	0.0038	10.21	3.73	0.000

The pooled false discovery rate is total FP divided by total selections across replicates. Because it is an aggregate ratio over the whole simulation rather than a per-replicate quantity, a replicate-level standard deviation is not applicable to it and none is reported. The mean FDP is the mean of the per-replicate proportion FP/(TP+FP), computed only over replicates that selected at least one pair, with the 0/0 case treated as undefined rather than as zero; its SD and MCSE are computed over that same subset. In the scenarios without true interactions the pooled FDR is exactly 1 by construction, since no true positive can exist, and the per-replicate FDP is identically 1 wherever it is defined, so its SD is zero.

Table S6b. Distribution of the number of interaction pairs selected per replicate

Scenario	Outcome	Method	0	1	2	3	4	5	6	7	8	9	10	11+
LM	continuous	glmnet_int	0.172	0.172	0.158	0.112	0.074	0.088	0.050	0.050	0.024	0.030	0.012	0.058
LM	continuous	hierNet	0.088	0.152	0.158	0.116	0.078	0.084	0.058	0.054	0.040	0.044	0.028	0.100
LMI	continuous	glmnet_int	0.000	0.002	0.006	0.006	0.056	0.088	0.104	0.122	0.112	0.100	0.088	0.316
LMI	continuous	hierNet	0.000	0.000	0.008	0.024	0.034	0.106	0.124	0.106	0.122	0.122	0.094	0.260
NM	continuous	hierNet	0.002	0.030	0.068	0.122	0.096	0.114	0.112	0.108	0.074	0.064	0.042	0.168
NM	continuous	glmnet_int	0.010	0.048	0.074	0.102	0.130	0.126	0.088	0.082	0.094	0.052	0.034	0.160

NMI	continuous	hierNet	0.000	0.000	0.006	0.020	0.036	0.086	0.098	0.088	0.142	0.128	0.112	0.284
NMI	continuous	glmnet_int	0.000	0.000	0.000	0.004	0.016	0.050	0.070	0.092	0.114	0.156	0.112	0.386
Logit	binary	hierNet	0.452	0.068	0.054	0.038	0.038	0.036	0.024	0.032	0.018	0.008	0.022	0.210
Logit	binary	glmnet_int	0.180	0.142	0.158	0.122	0.110	0.076	0.042	0.036	0.032	0.034	0.024	0.044
LogitI	binary	hierNet	0.086	0.042	0.060	0.072	0.060	0.076	0.072	0.052	0.054	0.056	0.046	0.324
LogitI	binary	glmnet_int	0.004	0.008	0.016	0.046	0.082	0.096	0.108	0.088	0.132	0.102	0.094	0.224
Nlogit	binary	glmnet_int	0.050	0.050	0.090	0.086	0.112	0.112	0.096	0.080	0.080	0.046	0.050	0.148
Nlogit	binary	hierNet	0.216	0.078	0.078	0.078	0.058	0.058	0.044	0.048	0.042	0.028	0.034	0.238
NlogitI	binary	glmnet_int	0.000	0.000	0.004	0.004	0.020	0.042	0.050	0.088	0.148	0.106	0.144	0.394
NlogitI	binary	hierNet	0.008	0.012	0.042	0.072	0.066	0.054	0.094	0.074	0.044	0.036	0.056	0.442

Proportion of the 500 replicates selecting each number of interaction pairs. This distribution is reported so that the pooled and mean-per-replicate false discovery summaries can be interpreted, and in particular so that the proportion of replicates for which the false discovery proportion is undefined is visible. Column "11+" aggregates all counts above 10.

Table S6c. Verification of the false discovery rate computation

Scenario	Outcome	Method	True pairs	Null pairs	Mean sensitivity	Mean FPR	FDR implied by rates	FDR pooled from counts	Identity gap	Mean replicate FDP	Pooled – mean FDP
LM	continuous	glmnet_int	0	45		0.0799	1.00000	1.00000	0.00e+00	1.00000	0.00000
LM	continuous	hierNet	0	45		0.1047	1.00000	1.00000	0.00e+00	1.00000	0.00000
LMI	continuous	glmnet_int	3	42	0.5887	0.1790	0.80978	0.80978	0.00e+00	0.78595	0.02383
LMI	continuous	hierNet	3	42	0.6060	0.1674	0.79458	0.79458	0.00e+00	0.76697	0.02760
NM	continuous	glmnet_int	0	45		0.1463	1.00000	1.00000	0.00e+00	1.00000	0.00000
NM	continuous	hierNet	0	45		0.1498	1.00000	1.00000	0.00e+00	1.00000	0.00000
NMI	continuous	glmnet_int	3	42	0.6780	0.1977	0.80321	0.80321	1.11e-16	0.78353	0.01969
NMI	continuous	hierNet	3	42	0.6513	0.1735	0.78857	0.78857	0.00e+00	0.76282	0.02575
Logit	binary	hierNet	0	45		0.1128	1.00000	1.00000	0.00e+00	1.00000	0.00000
Logit	binary	glmnet_int	0	45		0.0787	1.00000	1.00000	0.00e+00	1.00000	0.00000
LogitI	binary	hierNet	3	42	0.5320	0.1680	0.81553	0.81553	0.00e+00	0.73664	0.07889
LogitI	binary	glmnet_int	3	42	0.5733	0.1558	0.79182	0.79182	1.11e-16	0.76365	0.02817
Nlogit	binary	hierNet	0	45		0.1426	1.00000	1.00000	0.00e+00	1.00000	0.00000
Nlogit	binary	glmnet_int	0	45		0.1404	1.00000	1.00000	0.00e+00	1.00000	0.00000
NlogitI	binary	hierNet	3	42	0.6833	0.2097	0.81116	0.81116	0.00e+00	0.74165	0.06951
NlogitI	binary	glmnet_int	3	42	0.7153	0.1921	0.78990	0.78990	1.11e-16	0.76814	0.02176

Because the number of true and null candidate pairs is fixed across replicates, mean sensitivity × number of true pairs equals mean TP and mean FPR × number of null pairs equals mean FP. The pooled FDR is therefore algebraically identical to the FDR implied by the mean rates, and the identity gap is zero to machine precision in every cell; a non-zero value would indicate a bookkeeping error. The final column reports the substantive difference between the two reporting conventions, which is 0.02 to 0.08 and does not alter any conclusion.

Table S7. Ranking-based interaction detection

Scenario	Outcome	Method	Replicates	ROC AUC (pairs)	MCSE	Average precision	MCSE	Top-3 recall	MCSE	Mean rank of true pair	Pairs scored	Unusable replicates
LMI	continuous	glmnet_int	50	0.7227	0.0242	0.4104	0.0354	0.3267	0.0375	12.00	45.0	0.00
LMI	continuous	hierNet	50	0.7391	0.0256	0.4318	0.0398	0.3467	0.0425	11.91	45.0	0.00
LMI	continuous	BKMR	50	0.7583	0.0175	0.3541	0.0240	0.2800	0.0291	12.01	45.0	0.00
LMI	continuous	RF	50	0.6378	0.0165	0.1607	0.0226	0.1067	0.0293	22.41	45.0	0.00
NMI	continuous	glmnet_int	50	0.7628	0.0196	0.3892	0.0325	0.3133	0.0386	11.54	45.0	0.00
NMI	continuous	hierNet	50	0.7733	0.0205	0.4027	0.0317	0.3400	0.0387	11.17	45.0	0.00
NMI	continuous	BKMR	50	0.7705	0.0169	0.3582	0.0206	0.3133	0.0259	11.47	45.0	0.00
NMI	continuous	RF	50	0.6284	0.0151	0.1794	0.0212	0.1067	0.0260	20.18	45.0	0.00

LogitI	binary	glmnet_int	50	0.7275	0.0190	0.4030	0.0279	0.2867	0.0316	10.77	45.0	0.00
LogitI	binary	hierNet	50	0.7333	0.0214	0.4212	0.0296	0.3400	0.0308	11.11	45.0	0.00
LogitI	binary	BKMR	50	0.7805	0.0173	0.3809	0.0210	0.2867	0.0286	11.13	45.0	0.00
LogitI	binary	RF	50								0.0	1.00
NlogitI	binary	glmnet_int	50	0.7540	0.0188	0.4047	0.0283	0.3533	0.0307	11.61	45.0	0.00
NlogitI	binary	hierNet	50	0.7525	0.0181	0.4031	0.0241	0.3667	0.0305	12.31	45.0	0.00
NlogitI	binary	BKMR	50	0.7679	0.0151	0.3634	0.0184	0.3133	0.0241	11.72	45.0	0.00
NlogitI	binary	RF	50								0.0	1.00

All 45 candidate pairs are scored in every replicate and recall is measured against all three true pairs, so a method is not rewarded for failing to score a difficult pair. Random Forest interaction scores are Friedman H-statistics computed with iml; these could not be obtained for the binary scenarios because the classification predictor returned no pairwise statistic, and those cells are reported as unusable rather than omitted. Mean rank is out of 45; a value of 23 would correspond to random ordering. Based on 50 replicates per scenario with BKMR fitted at 25,000 iterations.

Table S8. Paired between-method differences in prediction, continuous outcomes

Scenario	Metric	Method	Reference	Pairs	Mean difference	MCSE	95% CI lower	95% CI upper	p
LM	MSE	glmnet_main	glmnet_int	500	-0.2074	0.0189	-0.2446	-0.1703	3.04e-25
LM	MSE	hierNet	glmnet_int	500	-0.0565	0.0143	-0.0845	-0.0285	8.51e-05
LM	MSE	rf	glmnet_int	500	1.0978	0.0340	1.0311	1.1646	1.99e-124
LM	MSE	BKMR	glmnet_int	500	2.5750	0.1322	2.3153	2.8347	2.73e-63
LM	MSE	qgcomp	glmnet_int	500	0.5433	0.0313	0.4818	0.6048	3.69e-53
LM	MSE	gWQS	glmnet_int	500	0.2948	0.0299	0.2360	0.3536	4.97e-21
LM	MSE	SuperLearner	glmnet_int	500	-0.1085	0.0216	-0.1508	-0.0661	6.76e-07
LM	MSE_oracle	glmnet_main	glmnet_int	500	-0.2124	0.0138	-0.2395	-0.1854	3.72e-44
LM	MSE_oracle	hierNet	glmnet_int	500	-0.0603	0.0117	-0.0833	-0.0373	3.84e-07
LM	MSE_oracle	rf	glmnet_int	500	1.0912	0.0166	1.0586	1.1238	5.43e-248
LM	MSE_oracle	BKMR	glmnet_int	500	2.6000	0.1294	2.3457	2.8543	3.30e-66
LM	MSE_oracle	qgcomp	glmnet_int	500	0.5748	0.0195	0.5366	0.6130	1.20e-111
LM	MSE_oracle	gWQS	glmnet_int	500	0.3106	0.0189	0.2734	0.3477	7.23e-49
LM	MSE_oracle	SuperLearner	glmnet_int	500	-0.1039	0.0151	-0.1336	-0.0741	1.97e-11
LM	Corr_oracle	glmnet_main	glmnet_int	500	0.0087	0.0008	0.0070	0.0104	9.64e-23
LM	Corr_oracle	hierNet	glmnet_int	500	0.0009	0.0006	-0.0004	0.0022	1.72e-01
LM	Corr_oracle	rf	glmnet_int	500	-0.0682	0.0010	-0.0702	-0.0662	3.64e-252
LM	Corr_oracle	BKMR	glmnet_int	500	-0.1826	0.0091	-0.2005	-0.1648	1.74e-66
LM	Corr_oracle	qgcomp	glmnet_int	500	-0.0396	0.0011	-0.0417	-0.0375	1.09e-145
LM	Corr_oracle	gWQS	glmnet_int	500	-0.0242	0.0010	-0.0261	-0.0222	5.45e-86
LM	Corr_oracle	SuperLearner	glmnet_int	500	0.0017	0.0009	-0.0001	0.0034	6.00e-02
LMI	MSE	glmnet_main	glmnet_int	500	1.9469	0.0554	1.8381	2.0557	4.19e-137
LMI	MSE	hierNet	glmnet_int	500	-0.3062	0.0292	-0.3635	-0.2488	2.22e-23
LMI	MSE	rf	glmnet_int	500	1.5789	0.0528	1.4751	1.6827	2.84e-113
LMI	MSE	BKMR	glmnet_int	500	4.6140	0.2370	4.1484	5.0797	3.18e-63
LMI	MSE	qgcomp	glmnet_int	500	3.0562	0.0702	2.9183	3.1942	5.20e-172
LMI	MSE	gWQS	glmnet_int	500	2.5687	0.0657	2.4396	2.6977	4.50e-154
LMI	MSE	SuperLearner	glmnet_int	500	0.9532	0.0451	0.8647	1.0418	2.59e-71
LMI	MSE_oracle	glmnet_main	glmnet_int	500	1.9246	0.0307	1.8643	1.9849	1.06e-238
LMI	MSE_oracle	hierNet	glmnet_int	500	-0.3044	0.0214	-0.3464	-0.2625	6.25e-39
LMI	MSE_oracle	rf	glmnet_int	500	1.5468	0.0295	1.4888	1.6049	8.06e-205
LMI	MSE_oracle	BKMR	glmnet_int	500	4.5497	0.2178	4.1218	4.9775	4.13e-70
LMI	MSE_oracle	qgcomp	glmnet_int	500	3.0647	0.0443	2.9776	3.1517	7.17e-258
LMI	MSE_oracle	gWQS	glmnet_int	500	2.5564	0.0399	2.4780	2.6348	5.69e-243
LMI	MSE_oracle	SuperLearner	glmnet_int	500	0.9393	0.0280	0.8842	0.9943	6.76e-130
LMI	Corr_oracle	glmnet_main	glmnet_int	500	-0.0839	0.0011	-0.0861	-0.0818	1.30e-276
LMI	Corr_oracle	hierNet	glmnet_int	500	0.0116	0.0008	0.0101	0.0131	1.68e-43
LMI	Corr_oracle	rf	glmnet_int	500	-0.0645	0.0012	-0.0668	-0.0622	3.34e-216
LMI	Corr_oracle	BKMR	glmnet_int	500	-0.2011	0.0095	-0.2197	-0.1825	8.34e-72
LMI	Corr_oracle	qgcomp	glmnet_int	500	-0.1346	0.0015	-0.1376	-0.1316	1.49e-305
LMI	Corr_oracle	gWQS	glmnet_int	500	-0.1134	0.0013	-0.1160	-0.1108	3.03e-299

LMI	Corr_oracle	SuperLearner	glmnet_int	500	-0.0424	0.0011	-0.0445	-0.0403	1.02e-155
NM	MSE	glmnet_main	glmnet_int	500	0.2095	0.0178	0.1747	0.2444	1.55e-28
NM	MSE	hierNet	glmnet_int	500	-0.1321	0.0113	-0.1543	-0.1099	4.24e-28
NM	MSE	rf	glmnet_int	500	0.4433	0.0229	0.3983	0.4883	1.07e-62
NM	MSE	BKMR	glmnet_int	500	1.4632	0.0930	1.2805	1.6458	1.32e-45
NM	MSE	qgcomp	glmnet_int	500	0.4838	0.0249	0.4349	0.5327	4.30e-63
NM	MSE	gWQS	glmnet_int	500	0.2964	0.0230	0.2512	0.3417	5.42e-33
NM	MSE	SuperLearner	glmnet_int	500	0.0619	0.0176	0.0274	0.0964	4.66e-04
NM	MSE_oracle	glmnet_main	glmnet_int	500	0.1996	0.0103	0.1794	0.2197	3.23e-63
NM	MSE_oracle	hierNet	glmnet_int	500	-0.1325	0.0082	-0.1487	-0.1164	3.17e-47
NM	MSE_oracle	rf	glmnet_int	500	0.4354	0.0116	0.4126	0.4583	3.85e-147
NM	MSE_oracle	BKMR	glmnet_int	500	1.4463	0.0888	1.2718	1.6208	3.97e-48
NM	MSE_oracle	qgcomp	glmnet_int	500	0.4574	0.0135	0.4308	0.4840	3.62e-131
NM	MSE_oracle	gWQS	glmnet_int	500	0.2607	0.0125	0.2361	0.2853	7.87e-70
NM	MSE_oracle	SuperLearner	glmnet_int	500	0.0514	0.0110	0.0299	0.0730	3.54e-06
NM	Corr_oracle	glmnet_main	glmnet_int	500	-0.0220	0.0009	-0.0239	-0.0202	4.18e-83
NM	Corr_oracle	hierNet	glmnet_int	500	0.0104	0.0007	0.0090	0.0117	2.93e-42
NM	Corr_oracle	rf	glmnet_int	500	-0.0407	0.0011	-0.0428	-0.0385	1.66e-146
NM	Corr_oracle	BKMR	glmnet_int	500	-0.1471	0.0089	-0.1645	-0.1297	1.25e-49
NM	Corr_oracle	qgcomp	glmnet_int	500	-0.0462	0.0012	-0.0485	-0.0438	2.33e-153
NM	Corr_oracle	gWQS	glmnet_int	500	-0.0286	0.0011	-0.0308	-0.0264	4.49e-93
NM	Corr_oracle	SuperLearner	glmnet_int	500	-0.0090	0.0010	-0.0110	-0.0070	1.48e-17
NMI	MSE	glmnet_main	glmnet_int	500	4.3055	0.0706	4.1669	4.4441	1.48e-233
NMI	MSE	hierNet	glmnet_int	500	-0.0583	0.0263	-0.1099	-0.0067	2.69e-02
NMI	MSE	rf	glmnet_int	500	1.4102	0.0539	1.3044	1.5160	9.44e-96
NMI	MSE	BKMR	glmnet_int	500	3.1073	0.2118	2.6913	3.5234	8.72e-41
NMI	MSE	qgcomp	glmnet_int	500	4.9801	0.0807	4.8215	5.1386	9.44e-236
NMI	MSE	gWQS	glmnet_int	500	4.4783	0.0767	4.3275	4.6290	4.24e-225
NMI	MSE	SuperLearner	glmnet_int	500	1.3262	0.0519	1.2242	1.4281	9.49e-93
NMI	MSE_oracle	glmnet_main	glmnet_int	500	4.3150	0.0422	4.2321	4.3980	0.00e+00
NMI	MSE_oracle	hierNet	glmnet_int	500	-0.0476	0.0187	-0.0843	-0.0110	1.10e-02
NMI	MSE_oracle	rf	glmnet_int	500	1.4539	0.0301	1.3948	1.5130	1.97e-190
NMI	MSE_oracle	BKMR	glmnet_int	500	3.2181	0.2094	2.8067	3.6294	6.29e-44
NMI	MSE_oracle	qgcomp	glmnet_int	500	4.9639	0.0492	4.8673	5.0606	0.00e+00
NMI	MSE_oracle	gWQS	glmnet_int	500	4.4580	0.0452	4.3691	4.5468	0.00e+00
NMI	MSE_oracle	SuperLearner	glmnet_int	500	1.3582	0.0320	1.2952	1.4211	1.57e-167
NMI	Corr_oracle	glmnet_main	glmnet_int	500	-0.2297	0.0018	-0.2333	-0.2261	0.00e+00
NMI	Corr_oracle	hierNet	glmnet_int	500	0.0019	0.0007	0.0005	0.0034	1.03e-02
NMI	Corr_oracle	rf	glmnet_int	500	-0.0691	0.0012	-0.0714	-0.0668	6.69e-226
NMI	Corr_oracle	BKMR	glmnet_int	500	-0.1489	0.0093	-0.1672	-0.1305	1.68e-46
NMI	Corr_oracle	qgcomp	glmnet_int	500	-0.2652	0.0018	-0.2688	-0.2616	0.00e+00
NMI	Corr_oracle	gWQS	glmnet_int	500	-0.2383	0.0016	-0.2415	-0.2352	0.00e+00
NMI	Corr_oracle	SuperLearner	glmnet_int	500	-0.0678	0.0013	-0.0704	-0.0652	2.86e-201

Differences are method minus glmnet_int computed within replicate, so the comparison is paired on identical simulated datasets and the Monte Carlo error of the difference is much smaller than that of either mean. Negative values favour the listed method. Intervals are 95% t-intervals on the paired differences across 500 replicates.

Table S9. Paired between-method differences in classification, binary outcomes

Scenario	Metric	Method	Reference	Pairs	Mean difference	MCSE	95% CI lower	95% CI upper	p
Logit	AUC	glmnet_main	glmnet_int	500	0.0014	0.0002	0.0010	0.0017	7.78e-13
Logit	AUC	hierNet	glmnet_int	500	-0.0019	0.0003	-0.0026	-0.0012	2.30e-08
Logit	AUC	rf	glmnet_int	500	-0.0193	0.0004	-0.0201	-0.0184	1.65e-176
Logit	AUC	BKMR	glmnet_int	500	-0.0076	0.0008	-0.0092	-0.0061	4.26e-20
Logit	AUC	qgcomp	glmnet_int	500	-0.0061	0.0004	-0.0068	-0.0054	3.41e-51
Logit	AUC	gWQS	glmnet_int	500	-0.0024	0.0003	-0.0030	-0.0017	5.67e-13
Logit	AUC	SuperLearner	glmnet_int	500	0.0001	0.0002	-0.0003	0.0006	5.78e-01

LogitI	AUC	glmnet_main	glmnet_int	500	-0.0003	0.0004	-0.0011	0.0005	4.55e-01
LogitI	AUC	hierNet	glmnet_int	500	-0.0000	0.0004	-0.0008	0.0008	9.69e-01
LogitI	AUC	rf	glmnet_int	500	-0.0165	0.0006	-0.0178	-0.0153	5.53e-96
LogitI	AUC	BKMR	glmnet_int	500	-0.0120	0.0012	-0.0143	-0.0097	6.01e-22
LogitI	AUC	qgcomp	glmnet_int	500	-0.0071	0.0005	-0.0081	-0.0061	3.81e-36
LogitI	AUC	gWQS	glmnet_int	500	-0.0021	0.0005	-0.0031	-0.0011	2.91e-05
LogitI	AUC	SuperLearner	glmnet_int	500	-0.0020	0.0004	-0.0029	-0.0012	3.07e-06
Nlogit	AUC	glmnet_main	glmnet_int	500	0.0019	0.0003	0.0014	0.0025	5.24e-11
Nlogit	AUC	hierNet	glmnet_int	500	-0.0001	0.0004	-0.0008	0.0006	7.76e-01
Nlogit	AUC	rf	glmnet_int	500	-0.0128	0.0006	-0.0139	-0.0117	4.77e-79
Nlogit	AUC	BKMR	glmnet_int	500	-0.0019	0.0007	-0.0032	-0.0005	6.35e-03
Nlogit	AUC	qgcomp	glmnet_int	500	-0.0031	0.0004	-0.0039	-0.0023	1.26e-12
Nlogit	AUC	gWQS	glmnet_int	500	0.0011	0.0004	0.0003	0.0019	6.58e-03
Nlogit	AUC	SuperLearner	glmnet_int	500	0.0011	0.0003	0.0005	0.0018	7.38e-04
NlogitI	AUC	glmnet_main	glmnet_int	500	-0.0239	0.0008	-0.0255	-0.0223	1.52e-108
NlogitI	AUC	hierNet	glmnet_int	500	-0.0034	0.0005	-0.0045	-0.0024	4.29e-10
NlogitI	AUC	rf	glmnet_int	500	-0.0092	0.0008	-0.0107	-0.0077	8.17e-30
NlogitI	AUC	BKMR	glmnet_int	500	-0.0087	0.0011	-0.0109	-0.0065	5.32e-14
NlogitI	AUC	qgcomp	glmnet_int	500	-0.0284	0.0009	-0.0301	-0.0266	1.11e-121
NlogitI	AUC	gWQS	glmnet_int	500	-0.0215	0.0009	-0.0232	-0.0198	4.64e-90
NlogitI	AUC	SuperLearner	glmnet_int	500	-0.0079	0.0007	-0.0093	-0.0066	1.64e-27

Differences are method minus glmnet_int in AUC, computed within replicate. Positive values favour the listed method.

Table S10. Paired between-method differences in interaction detection

Scenario	Outcome	Metric	Method	Reference	Pairs	Mean difference	MCSE	95% CI lower	95% CI upper	p
LM	continuous	FDR	hierNet	glmnet_int	387	0.0000	0.0000			
LM	continuous	FPR	hierNet	glmnet_int	500	0.0248	0.0042	0.0166	0.0331	5.68e-09
LM	continuous	N_selected	hierNet	glmnet_int	500	1.1180	0.1886	0.7475	1.4885	5.68e-09
LMI	continuous	Sensitivity	hierNet	glmnet_int	500	0.0173	0.0095	-0.0013	0.0360	6.87e-02
LMI	continuous	FDR	hierNet	glmnet_int	500	-0.0190	0.0056	-0.0299	-0.0080	7.26e-04
LMI	continuous	FPR	hierNet	glmnet_int	500	-0.0116	0.0043	-0.0201	-0.0030	7.99e-03
LMI	continuous	N_selected	hierNet	glmnet_int	500	-0.4340	0.1919	-0.8110	-0.0570	2.41e-02
NM	continuous	FPR	hierNet	glmnet_int	500	0.0036	0.0044	-0.0051	0.0122	4.20e-01
NM	continuous	N_selected	hierNet	glmnet_int	500	0.1600	0.1981	-0.2292	0.5492	4.20e-01
NM	continuous	FDR	hierNet	glmnet_int	495	0.0000	0.0000			
NMI	continuous	Sensitivity	hierNet	glmnet_int	500	-0.0267	0.0090	-0.0443	-0.0090	3.10e-03
NMI	continuous	N_selected	hierNet	glmnet_int	500	-1.0940	0.1785	-1.4447	-0.7433	1.79e-09
NMI	continuous	FPR	hierNet	glmnet_int	500	-0.0241	0.0040	-0.0321	-0.0162	4.54e-09
NMI	continuous	FDR	hierNet	glmnet_int	500	-0.0207	0.0045	-0.0295	-0.0119	5.36e-06
Logit	binary	FPR	hierNet	glmnet_int	500	0.0341	0.0073	0.0198	0.0484	3.45e-06
Logit	binary	FDR	hierNet	glmnet_int	232	0.0000	0.0000			
Logit	binary	N_selected	hierNet	glmnet_int	500	1.5360	0.3272	0.8932	2.1788	3.45e-06
LogitI	binary	Sensitivity	hierNet	glmnet_int	500	-0.0413	0.0131	-0.0670	-0.0156	1.68e-03
LogitI	binary	FDR	hierNet	glmnet_int	457	-0.0283	0.0098	-0.0476	-0.0089	4.27e-03
LogitI	binary	FPR	hierNet	glmnet_int	500	0.0122	0.0072	-0.0019	0.0264	9.05e-02
LogitI	binary	N_selected	hierNet	glmnet_int	500	0.3900	0.3251	-0.2487	1.0287	2.31e-01
Nlogit	binary	FDR	hierNet	glmnet_int	381	0.0000	0.0000			
Nlogit	binary	FPR	hierNet	glmnet_int	500	0.0022	0.0074	-0.0123	0.0167	7.68e-01
Nlogit	binary	N_selected	hierNet	glmnet_int	500	0.0980	0.3319	-0.5541	0.7501	7.68e-01
NlogitI	binary	N_selected	hierNet	glmnet_int	500	0.6420	0.3315	-0.0093	1.2933	5.34e-02
NlogitI	binary	Sensitivity	hierNet	glmnet_int	500	-0.0320	0.0115	-0.0546	-0.0094	5.63e-03
NlogitI	binary	FDR	hierNet	glmnet_int	496	-0.0268	0.0077	-0.0420	-0.0116	5.65e-04
NlogitI	binary	FPR	hierNet	glmnet_int	500	0.0176	0.0075	0.0029	0.0323	1.94e-02

Differences are hierNet minus glmnet_int computed within replicate. For the false discovery proportion the number of paired replicates is smaller than 500 in the scenarios without true interactions because the proportion is undefined whenever a replicate selects no pair; in those scenarios every defined value equals 1 for both methods, so the paired difference is exactly zero and no interval is estimable.

Table S11. BKMR sampling budget: performance at 2,000 and 25,000 MCMC iterations

Scenario	Outcome	Iterations	Fits	Oracle MSE	SD	r (oracle)	SD	AUC	SD	Runtime (s)	Seconds per 1,000 iterations
LM	continuous	25000	50	0.731	0.259	0.9628	0.0129			6265.3	250.6
LM	continuous	2000	50	3.841	2.977	0.7565	0.1956			236.0	118.0
LMI	continuous	25000	50	2.081	0.712	0.9243	0.0242			6924.5	277.0
LMI	continuous	2000	50	6.382	4.845	0.7412	0.2014			236.1	118.0
NM	continuous	25000	50	0.610	0.231	0.9525	0.0173			7034.5	281.4
NM	continuous	2000	50	1.835	1.892	0.8329	0.1801			242.3	121.1
NMI	continuous	25000	50	1.935	0.849	0.9257	0.0301			6662.4	266.5
NMI	continuous	2000	50	5.291	4.751	0.7668	0.2160			245.1	122.6
Logit	binary	25000	50					0.7758	0.0173	12472.0	498.9
Logit	binary	2000	50					0.7690	0.0221	265.8	132.9
LogitI	binary	25000	50					0.7391	0.0195	15357.8	614.3
LogitI	binary	2000	50					0.7288	0.0313	376.6	188.3
Nlogit	binary	25000	50					0.7558	0.0241	13121.4	524.9
Nlogit	binary	2000	50					0.7465	0.0287	305.6	152.8
NlogitI	binary	25000	50					0.7034	0.0224	13430.7	537.2
NlogitI	binary	2000	50					0.6880	0.0355	286.5	143.3

Fifty paired replicates per scenario, with both budgets applied to the same simulated datasets. The replicate-to-replicate standard deviation of oracle correlation is reported alongside the mean because the reduction in variability at the longer budget is as large as the change in the mean.

Table S12. Paired within-replicate effect of the BKMR sampling budget

Scenario	Outcome	Metric	Replicates	Mean (25,000 – 2,000)	SD	MCSE	95% CI lower	95% CI upper	p
LM	continuous	MSE	50	-3.1802	3.2407	0.4583	-4.1012	-2.2592	8.24e-09
LM	continuous	MSE_oracle	50	-3.1095	2.9148	0.4122	-3.9378	-2.2811	9.60e-10
LM	continuous	Corr_observed	50	0.0942	0.0939	0.0133	0.0675	0.1209	4.85e-09
LM	continuous	Corr_oracle	50	0.2063	0.1945	0.0275	0.1511	0.2616	1.12e-09
LMI	continuous	MSE	50	-4.4806	5.3718	0.7597	-6.0072	-2.9539	3.36e-07
LMI	continuous	MSE_oracle	50	-4.3007	4.7662	0.6740	-5.6553	-2.9462	6.05e-08
LMI	continuous	Corr_observed	50	0.0840	0.0985	0.0139	0.0560	0.1120	2.08e-07
LMI	continuous	Corr_oracle	50	0.1831	0.1993	0.0282	0.1265	0.2398	3.98e-08
NM	continuous	MSE	50	-1.1481	1.7721	0.2506	-1.6517	-0.6444	3.20e-05
NM	continuous	MSE_oracle	50	-1.2248	1.8663	0.2639	-1.7552	-0.6944	2.62e-05
NM	continuous	Corr_observed	50	0.0500	0.0759	0.0107	0.0285	0.0716	2.45e-05
NM	continuous	Corr_oracle	50	0.1196	0.1813	0.0256	0.0681	0.1711	2.41e-05
NMI	continuous	MSE	50	-3.4995	5.0630	0.7160	-4.9384	-2.0606	1.14e-05
NMI	continuous	MSE_oracle	50	-3.3560	4.7340	0.6695	-4.7014	-2.0106	7.40e-06
NMI	continuous	Corr_observed	50	0.0748	0.1057	0.0150	0.0447	0.1048	7.74e-06
NMI	continuous	Corr_oracle	50	0.1589	0.2168	0.0307	0.0972	0.2205	4.15e-06
Logit	binary	AUC	50	0.0068	0.0171	0.0024	0.0019	0.0117	7.16e-03
LogitI	binary	AUC	50	0.0103	0.0282	0.0040	0.0023	0.0183	1.28e-02
Nlogit	binary	AUC	50	0.0093	0.0202	0.0029	0.0035	0.0150	2.12e-03
NlogitI	binary	AUC	50	0.0154	0.0273	0.0039	0.0077	0.0232	2.12e-04

Negative values for MSE and oracle MSE and positive values for correlation and AUC indicate improvement at the longer budget. Because each replicate contributes a matched pair of fits to the same dataset, these intervals isolate the budget effect from between-dataset variation.

Table S13. BKMR multi-chain convergence diagnostics

Outcome	Scenario	Iterations	Chains	Monitored parameters	Median $\hat{R}$	Max $\hat{R}$	Prop. $\hat{R}$ > 1.1	Min ESS	Median ESS	Max ESS
binary	LogitI	2000	4	55	1.288	8.151	0.764	19.8	81.1	186.6
binary	LogitI	25000	4	55	1.020	1.224	0.018	183.3	628.9	1060.9
continuous	LMI	2000	4	60	1.503	6.100	0.933	7.5	75.8	645.0
continuous	LMI	25000	4	60	1.017	1.249	0.050	227.2	809.4	2000.0

Four independent chains from different random initializations on each of five datasets, monitoring the ten kernel bandwidth parameters r1 to r10, the variance component lambda1, and, for continuous outcomes, the residual variance. The Gelman-Rubin statistic and effective sample size are computed with coda on the unthinned post-burn-in draws. The monitored-parameter count is the number of parameter-by-dataset combinations. Trace plots for every dataset and budget are provided as separate image files.

Table S13b. BKMR sampler configuration for the binomial family

kmbayes configuration	Proportion of fits completing
varsel=TRUE, est.h=TRUE	1.00
varsel=FALSE	0.00
varsel=TRUE, default priors	0.00
varsel=TRUE, r.prior=invunif	0.00

Diagnostic grid over four sampler configurations, three simulated datasets per binary scenario and all four binary scenarios, at a short diagnostic budget. Under the default configuration, kmbayes with family = "binomial" terminated with a non-positive-definite augmented kernel matrix in every fit. Setting est.h = TRUE, which samples the exposure-response surface explicitly rather than marginalizing it out, completed in every fit. This configuration is used for all BKMR results reported in the manuscript, for both outcome families, so that the sampler is identical across outcome types. Forty chain-level failures under the default configuration were logged during the convergence arm and are retained in the deposited output.

Table S14. Sensitivity to sample size

Scenario	Method	Metric	250	500	1000
LMI	glmnet_main	Oracle MSE	4.364	3.683	3.283
LMI	glmnet_int	Oracle MSE	3.120	1.641	0.975
LMI	hierNet	Oracle MSE	2.668	1.389	0.884
LMI	rf	Oracle MSE	4.289	3.304	2.602
LMI	BKMR	Oracle MSE	4.505	3.428	8.290
LMI	qgcomp	Oracle MSE	6.167	4.857	4.192
LMI	gWQS	Oracle MSE	5.115	4.040	4.000
LMI	SuperLearner	Oracle MSE	3.779	2.688	2.060
NMI	glmnet_main	Oracle MSE	7.028	6.511	6.025
NMI	glmnet_int	Oracle MSE	3.522	1.925	1.209
NMI	hierNet	Oracle MSE	3.048	1.889	1.279
NMI	rf	Oracle MSE	4.457	3.462	2.624
NMI	BKMR	Oracle MSE	3.769	3.771	5.394
NMI	qgcomp	Oracle MSE	8.177	7.173	6.392
NMI	gWQS	Oracle MSE	6.667	7.067	6.280
NMI	SuperLearner	Oracle MSE	4.530	3.345	2.510

Scenario	Method	Metric	250	500	1000

LogitI	glmnet_main	AUC	0.7205	0.7227	0.7377
LogitI	glmnet_int	AUC	0.7178	0.7246	0.7388
LogitI	hierNet	AUC	0.7187	0.7238	0.7396
LogitI	rf	AUC	0.7059	0.7091	0.7198
LogitI	BKMR	AUC	0.6975	0.7160	0.7156
LogitI	qgcomp	AUC	0.7112	0.7144	0.7327
LogitI	gWQS	AUC	0.7078	0.7202	0.7331
LogitI	SuperLearner	AUC	0.7156	0.7215	0.7351
NlogitI	glmnet_main	AUC	0.6679	0.6809	0.6822
NlogitI	glmnet_int	AUC	0.6823	0.7010	0.7099
NlogitI	hierNet	AUC	0.6886	0.6986	0.7090
NlogitI	rf	AUC	0.6872	0.6960	0.6981
NlogitI	BKMR	AUC	0.6852	0.7046	0.6922
NlogitI	qgcomp	AUC	0.6608	0.6778	0.6806
NlogitI	gWQS	AUC	0.6770	0.6832	0.6849
NlogitI	SuperLearner	AUC	0.6875	0.6975	0.6999

Columns represent the equal training and test sample sizes used within each condition. All eight methods were evaluated at every level: six methods at 50 replicates and BKMR and gWQS at 15 replicates, so that no method is dropped from the generalizability assessment. Oracle MSE is reported for continuous outcomes and AUC for binary outcomes.

Table S15. Sensitivity to the exposure correlation structure

Scenario	Method	Metric	low	primary	high
LMI	glmnet_main	Oracle MSE	2.255	3.725	5.624
LMI	glmnet_int	Oracle MSE	1.493	1.918	1.973
LMI	hierNet	Oracle MSE	1.275	1.607	1.653
LMI	rf	Oracle MSE	2.413	3.384	4.646
LMI	BKMR	Oracle MSE	5.039	5.788	4.687
LMI	qgcomp	Oracle MSE	2.817	4.856	7.635
LMI	gWQS	Oracle MSE	2.546	4.298	6.838
LMI	SuperLearner	Oracle MSE	2.027	2.836	3.703
NMI	glmnet_main	Oracle MSE	3.847	6.434	9.456
NMI	glmnet_int	Oracle MSE	1.946	2.064	2.063
NMI	hierNet	Oracle MSE	1.900	2.056	2.051
NMI	rf	Oracle MSE	2.423	3.454	4.816
NMI	BKMR	Oracle MSE	4.085	6.440	4.818
NMI	qgcomp	Oracle MSE	4.122	7.030	10.768
NMI	gWQS	Oracle MSE	3.732	6.899	9.732
NMI	SuperLearner	Oracle MSE	2.475	3.420	4.362

Scenario	Method	Metric	low	primary	high
LogitI	glmnet_main	AUC	0.7314	0.7326	0.7284
LogitI	glmnet_int	AUC	0.7350	0.7325	0.7315
LogitI	hierNet	AUC	0.7344	0.7308	0.7326
LogitI	rf	AUC	0.7207	0.7141	0.7102
LogitI	BKMR	AUC	0.7282	0.7214	0.7357
LogitI	qgcomp	AUC	0.7229	0.7237	0.7198
LogitI	gWQS	AUC	0.7318	0.7266	0.7308
LogitI	SuperLearner	AUC	0.7315	0.7314	0.7257
NlogitI	glmnet_main	AUC	0.6859	0.6781	0.6773
NlogitI	glmnet_int	AUC	0.6980	0.7025	0.7127
NlogitI	hierNet	AUC	0.6973	0.7000	0.7122
NlogitI	rf	AUC	0.6975	0.6958	0.7005
NlogitI	BKMR	AUC	0.6890	0.6844	0.7109

NlogitI	qgcomp	AUC	0.6823	0.6740	0.6713
NlogitI	gWQS	AUC	0.6790	0.6774	0.6831
NlogitI	SuperLearner	AUC	0.6973	0.6964	0.7026

Correlation configurations are low (0.3 within block, 0.05 between), primary (0.6, 0.1) and high (0.8, 0.3). Replicate counts are as in Table S14.

Table S16. Sensitivity to signal strength (target R^2)

Scenario	Method	Metric	0.2	0.3	0.4
LMI	glmnet_main	Oracle MSE	3.803	3.526	3.327
LMI	glmnet_int	Oracle MSE	1.858	1.270	0.843
LMI	hierNet	Oracle MSE	1.407	1.006	0.730
LMI	rf	Oracle MSE	3.404	2.328	1.740
LMI	BKMR	Oracle MSE	5.144	2.301	1.082
LMI	qgcomp	Oracle MSE	5.030	4.445	4.216
LMI	gWQS	Oracle MSE	4.343	4.061	4.142
LMI	SuperLearner	Oracle MSE	2.698	2.163	1.744
NMI	glmnet_main	Oracle MSE	6.531	6.014	6.149
NMI	glmnet_int	Oracle MSE	1.945	1.388	1.186
NMI	hierNet	Oracle MSE	1.905	1.356	1.160
NMI	rf	Oracle MSE	3.320	2.328	2.019
NMI	BKMR	Oracle MSE	5.296	1.691	1.360
NMI	qgcomp	Oracle MSE	7.265	6.386	6.469
NMI	gWQS	Oracle MSE	6.552	6.295	6.666
NMI	SuperLearner	Oracle MSE	3.294	2.351	2.084

The target R^2 governs the continuous outcome only, since the binary data-generating process is calibrated through the latent-scale standard deviation of the linear predictor rather than through an error variance. The corresponding binary signal-strength analysis is reported in Table S2b, with method-specific AUC results in Table S16b.

Table S16b. Classification performance across the three binary signal-strength levels

Scenario	Method	Replicates	0.75	1.25	1.75
LogitI	glmnet_main	50	0.6508	0.7341	0.7917
LogitI	glmnet_int	50	0.6485	0.7340	0.7921
LogitI	hierNet	50	0.6520	0.7333	0.7927
LogitI	rf	50	0.6324	0.7163	0.7796
LogitI	BKMR	15	0.6209	0.7218	0.7889
LogitI	qgcomp	50	0.6416	0.7264	0.7855
LogitI	gWQS	15	0.6495	0.7362	0.7845
LogitI	SuperLearner	50	0.6494	0.7313	0.7920
NlogitI	glmnet_main	50	0.6270	0.6795	0.7350
NlogitI	glmnet_int	50	0.6352	0.7059	0.7674
NlogitI	hierNet	50	0.6365	0.7029	0.7664
NlogitI	rf	50	0.6242	0.6949	0.7600
NlogitI	BKMR	15	0.6087	0.6825	0.7766
NlogitI	qgcomp	50	0.6202	0.6737	0.7331
NlogitI	gWQS	15	0.6263	0.6747	0.7400
NlogitI	SuperLearner	50	0.6319	0.6943	0.7603

Mean AUC by method and target latent-scale standard deviation of the linear predictor. The corresponding oracle Tjur and McFadden values for each level are given in Table S2b, which places the three levels on an interpretable signal scale. Six methods were evaluated over 50 replicates per setting and BKMR and gWQS over 15. Discrimination improves substantially with signal strength for every method, and the interaction-capable methods separate from the additive-only ones most clearly in the nonlinear scenario at the highest signal level.

Table S17. Pure-product interaction variant: detection

Scenario	Outcome	Method	Replicates	Sensitivity	MCSE	Specificity	FDR (pooled)	FPR	Pairs selected
LMI_pure	continuous	glmnet_int	50	0.6133	0.0373	0.8324	0.7928	0.1676	8.88
LMI_pure	continuous	hierNet	50	0.6200	0.0447	0.8143	0.8075	0.1857	9.66
NMI_pure	continuous	glmnet_int	50	0.7267	0.0339	0.8157	0.7802	0.1843	9.92
NMI_pure	continuous	hierNet	50	0.7133	0.0369	0.8200	0.7794	0.1800	9.70
LogitI_pure	binary	hierNet	50	0.4933	0.0478	0.8895	0.7582	0.1105	6.12
LogitI_pure	binary	glmnet_int	50	0.5667	0.0384	0.8662	0.7678	0.1338	7.32
NlogitI_pure	binary	hierNet	50	0.6667	0.0381	0.8071	0.8020	0.1929	10.10
NlogitI_pure	binary	glmnet_int	50	0.7467	0.0351	0.8200	0.7714	0.1800	9.80

The interaction function is replaced by the pure multiplicative form g(Xk, Xl) = 0.6·Xk·Xl, removing the quadratic threshold term and leaving a data-generating process in which every interaction is a clean product. Sensitivity and the overall comparability of the two enumerating methods are preserved, although the pooled false discovery ordering shifts in two of the four scenarios: hierNet holds the lower pooled rate in LMI but glmnet_int does in LMI_pure, and the reverse occurs between LogitI and LogitI_pure. The primary conclusion that neither enumerating method is uniformly superior therefore does not depend on the composite interaction form. Fifty replicates per scenario.

Table S17b. Prediction under the pure-product variant

Scenario	Method	MSE	Oracle MSE	MCSE	r (oracle)
LMI_pure	glmnet_main	43.851	2.765	0.0778	0.8717
LMI_pure	glmnet_int	42.458	1.476	0.0852	0.9383
LMI_pure	hierNet	42.222	1.346	0.1108	0.9439
LMI_pure	rf	43.705	2.762	0.0623	0.8733
LMI_pure	BKMR	45.806	4.977	0.5759	0.7514
LMI_pure	qgcomp	44.599	3.587	0.1019	0.8276
LMI_pure	gWQS	44.282	3.268	0.0872	0.8440
LMI_pure	SuperLearner	43.221	2.182	0.0637	0.9010
NMI_pure	glmnet_main	41.865	5.058	0.1329	0.6962
NMI_pure	glmnet_int	38.562	1.566	0.0732	0.9260
NMI_pure	hierNet	38.559	1.546	0.0748	0.9253
NMI_pure	rf	39.733	2.773	0.0753	0.8526
NMI_pure	BKMR	41.230	4.170	0.5204	0.7752
NMI_pure	qgcomp	42.260	5.450	0.1368	0.6711
NMI_pure	gWQS	41.919	5.112	0.1232	0.6925
NMI_pure	SuperLearner	39.672	2.725	0.0927	0.8517

Scenario	Method	AUC	MCSE	Oracle AUC
LogitI_pure	glmnet_main	0.7389	0.0027	0.7540
LogitI_pure	glmnet_int	0.7401	0.0029	0.7540
LogitI_pure	hierNet	0.7403	0.0029	0.7540
LogitI_pure	rf	0.7213	0.0026	0.7540
LogitI_pure	BKMR	0.7299	0.0039	0.7540
LogitI_pure	qgcomp	0.7304	0.0025	0.7540
LogitI_pure	gWQS	0.7358	0.0026	0.7540
LogitI_pure	SuperLearner	0.7378	0.0026	0.7540
NlogitI_pure	glmnet_main	0.6868	0.0040	0.7393
NlogitI_pure	glmnet_int	0.7127	0.0037	0.7393
NlogitI_pure	hierNet	0.7096	0.0039	0.7393
NlogitI_pure	rf	0.7042	0.0038	0.7393
NlogitI_pure	BKMR	0.7107	0.0042	0.7393

NlogitI_pure	qgcomp	0.6827	0.0039	0.7393
NlogitI_pure	gWQS	0.6890	0.0038	0.7393
NlogitI_pure	SuperLearner	0.7034	0.0038	0.7393

Fifty replicates per scenario, all eight methods.

Table S18. Computational cost of the study, measured from the completed runs

Analysis stage	Model fits	Measured core-hours	Wall-clock hours on 64 workers
Primary benchmark (8 scenarios x 500 replicates x 8 methods)	32000	899.3	14.05
BKMR sampling-budget experiment (2,000 vs 25,000 iterations)	800	1,159.2	18.11
Sample-size sensitivity	3960	31.8	0.50
Exposure-correlation sensitivity	3960	26.9	0.42
Continuous signal-strength sensitivity	1980	8.1	0.13
Binary signal-strength sensitivity	1980	16.7	0.26
Total (instrumented stages)	44680	2,142.1	33.47

Core-hours are the summed wall-clock fitting time actually recorded for every model fit in each stage, not a prior estimate. The wall-clock column divides by the 64 workers used, and is therefore a lower bound on elapsed time because it excludes data generation, scoring and input/output. Three further stages did not record per-fit timing and are excluded from the total: the multi-chain convergence diagnostics (80 BKMR fits, half at 25,000 iterations), the interaction-ranking benchmark (200 replicates with BKMR at 25,000 iterations plus 45 bivariate contrasts each), and the hierNet tolerance and pure-product analyses. The BKMR sampling-budget experiment accounts for more than half the instrumented cost from only 800 fits, which quantifies the price of the 25,000-iteration budget. All analyses ran on a single 64-core node with one computational thread per worker to prevent nested parallelism. Because each replicate seeds itself, serial and parallel execution return identical results; this was verified before the production run.
